# Tuning Fatty Acid Profile and Yield in *Pichia pastoris*

**DOI:** 10.3390/bioengineering10121412

**Published:** 2023-12-12

**Authors:** Simon Kobalter, Alena Voit, Myria Bekerle-Bogner, Haris Rudalija, Anne Haas, Tamara Wriessnegger, Harald Pichler

**Affiliations:** 1 Austrian Centre of Industrial Biotechnology (acib GmbH), Petersgasse 14, 8010 Graz, Austria; simonkobalter@acib.at (S.K.);; 2 Institute of Molecular Biotechnology, Graz University of Technology, NAWI Graz, BioTechMed Graz, Petersgasse 14, 8010 Graz, Austria

**Keywords:** yeast, free fatty acids, metabolic engineering, palmitoleic acid, *Pichia pastoris*, Komagataella phaffii

## Abstract

Fatty acids have been supplied for diverse non-food, industrial applications from plant oils and animal fats for many decades. Due to the massively increasing world population demanding a nutritious diet and the thrive to provide feedstocks for industrial production lines in a sustainable way, i.e., independent from food supply chains, alternative fatty acid sources have massively gained in importance. Carbohydrate-rich side-streams of agricultural production, e.g., molasses, lignocellulosic waste, glycerol from biodiesel production, and even CO_2_, are considered and employed as carbon sources for the fermentative accumulation of fatty acids in selected microbial hosts. While certain fatty acid species are readily accumulated in native microbial metabolic routes, other fatty acid species are scarce, and host strains need to be metabolically engineered for their high-level production. We report the metabolic engineering of *Pichia pastoris* to produce palmitoleic acid from glucose and discuss the beneficial and detrimental engineering steps in detail. Fatty acid secretion was achieved through the deletion of fatty acyl-CoA synthetases and overexpression of the truncated *E. coli* thioesterase ‘*TesA*. The best strains secreted >1 g/L free fatty acids into the culture medium. Additionally, the introduction of C16-specific ∆9-desaturases and fatty acid synthases, coupled with improved cultivation conditions, increased the palmitoleic acid content from 5.5% to 22%.

## 1. Introduction

Fatty acids (FAs) are fundamental constituents of the metabolic processes in all living organisms, serving as pivotal precursors for phospholipids essential in cell membrane synthesis. Additionally, FAs play a vital role in the formation of carbon- and energy-storage molecules, such as triacylglycerols (TAGs) and polyhydroxyalkanoates (PHAs) [1,2,3,4]. In addition to their crucial cellular significance, FAs and their derivatives (e.g., alka(e)nes, fatty alcohols, fatty acid esters, hydroxy fatty acids) exhibit significant industrial importance as versatile building blocks for a wide array of commercially relevant compounds, including detergents, soaps, lubricants, biofuels, cosmetics, and pharmaceutical products, among others [5,6]. Traditionally, the sourcing of FAs and their derivatives has heavily relied on animal fats and plant oils. However, the escalating reliance on edible oils, particularly palm oil, as feedstock for chemical and biofuel production has raised substantial environmental concerns.

Addressing these sustainability issues, research has embarked on investigating microbial free fatty acid (FFA) production, especially from non-edible carbon sources (e.g., lignocellulosic biomass, waste streams, glycerol), leading to promising eco-friendly production routes. Microorganisms offer numerous advantages over higher eukaryotic organisms for FFA production, including a simpler metabolism, being more amenable to genetic engineering, and demonstrating faster growth rates and safe production, making them well-suited for large-scale industrial applications. Furthermore, the enhanced accessibility of reaction/pathway and omics databases, genetic engineering tools, and high-throughput screening methods has facilitated the development of synthetic microbial cell factories proficient in the synthesis of diverse industrially relevant compounds with remarkable efficiency [7,8]. Nonetheless, the efficient microbial production of FFAs remains a major challenge due to the tight control and regulation of FA metabolism, as most native microorganisms do not support the biosynthesis of excess FAs beyond their metabolic demands through a set of regulatory mechanisms. Hence, in order to produce FFAs and derivatives thereof for commercial exploitation, non-oleaginous and oleaginous organisms have to be extensively engineered in their metabolic pathways.

*Escherichia coli*, *Saccharomyces cerevisiae*, and oleaginous yeasts like *Yarrowia lipolytica* and *Rhodosporidium toruloides* are commonly utilized microbial chassis in engineering approaches for optimized FA production [5,9,10]. *E. coli* has been intensively investigated in regard to its fatty acid biosynthesis and regulation [11,12]. Beyond the abundant genetic information, the benefits of its fast growth and easy genetic manipulation by numerous available genetic tools make *E. coli* a frequently used host, engineered for the elevated production of FFAs and derivatives [5,6,13,14,15,16,17,18,19]. However, when considering products intended for food and pharmaceutical applications, it is important to use microorganisms that are classified as “generally recognized as safe” (GRAS). This approach helps to address any potential safety concerns raised by the public. *S. cerevisiae* is one prime example of well-studied GRAS strains. It has been extensively employed in research studies focused on enhancing FA production through metabolic engineering strategies. Several combinatorial, multi-pathway attempts yielded considerable amounts of extracellular and intracellular lipids in *S. cerevisiae*, as recently reviewed in [20].

Oleaginous yeasts, such as *Yarrowia lipolytica*, *Rhodosporidium toruloides*, or *Lipomyces starkeyi*, are known for their natural ability to accumulate lipids up to 70% of their CDW under nutrient-limiting conditions, and most of the lipids are stored in the form of triacylglycerols (TAGs) [21]. These yeasts, which also have the GRAS status, harbor robust lipid synthesis pathways and have been further engineered for enhanced FA and oleochemical production [22,23,24] or for tailoring the chain-length of produced FAs and their derivatives [25,26].

The properties and nutritional value of natural fats, oils, and their derived oleochemicals depend primarily on the carbon chain-length and degree of saturation of the fatty acids they contain [27,28,29]. Recently, the biotechnological production of tailored chain-length fatty acids has gained importance, as not all fatty acid species are highly abundant in natural sources. Oleic acid (C18:1, OA) is abundantly found in various natural sources, especially plants and fruits (e.g., olive (55–83%), canola (62–64%), or avocado (59–62%)) [30]. Oleic acid’s presence contributes to the fluidity and stability of cell membranes, affecting cell signaling and physiological functions [31]. In contrast, the content of palmitoleic acid (C16:1, POA) in main vegetable oils is very low (<2%) [32], and alternative sourcing of POA has gained significant interest from industries, as several potential health benefits have been ascribed to POA, such as the attenuation of inflammation and the prevention of cardiovascular diseases or diabetes [33,34,35,36]. Furthermore, POA exhibits promise for utilization as a cleansing agent in skincare and medical products, owing to its specific bactericidal activity against *Staphylococcus aureus* [37]. Currently, its primary sources involve marine organisms and selected plants, particularly *Macadamia integrifolia* (15–22% POA in macadamia oil) and sea buckthorn (25–35% POA), as highlighted by Hu et al. [34] and Solà Marsiñach and Cuenca [38]. The cultivation and commercialization of plants rich in POA on a larger scale is hindered by low yield, small seed size, and limited geographic distribution; hence, there is a need to establish a sustainable and cost-effective production method as an alternative to the current POA extraction procedures.

The yeast *Pichia pastoris* (syn. *Komagataella phaffii*) has gained significant attention in biotechnological production processes due to its efficient protein expression system, especially when using its strong alcohol oxidase (AOX1) promoter. Products made by *P. pastoris* have been classified as GRAS. Thus, this methylotrophic yeast has become a preferred host organism for the production of recombinant proteins, enzymes, and other biomolecules [39,40,41,42]. In contrast to *S. cerevisiae*, *P. pastoris* is a Crabtree-negative yeast [43], which facilitates the application of this yeast for high-cell-density fermentation in industrial processes. Here, we aim to develop a *P. pastoris* strain for high-level fatty acid production from sustainable carbon sources, such as glucose or glycerol [39,44]. Our objectives encompass two linked approaches: firstly, the enhancement of overall FFA production, and secondly, the specific synthesis of POA at high titers. Thus, we have manipulated the metabolic pathway of the yeast *P. pastoris* towards a high-POA phenotype and have evaluated different strategies for FFA over-production in *P. pastoris*.

## 2. Materials and Methods

### 2.1. Chemicals, Media, and Cultivation Conditions

All the media components and laboratory reagents were sourced from Carl Roth GmbH & Co. KG (Karlsruhe, Germany), unless stated otherwise. Bacto^TM^ yeast extract was purchased from BD (Becton Dickinson, Heidelberg, Germany) while the yeast nitrogen base without amino acids and without ammonium sulfate was obtained from ForMedium^TM^ (Swaffham, UK). Antifoam 204 was obtained from Sigma-Aldrich, Vienna, Austria. Zeocin^®^ was purchased from InvivoGen (Toulouse, France). For plasmid assembly and propagation, *E. coli* TOP10 F′ obtained from Life Technologies (Vienna, Austria) was utilized. Phusion^TM^ High-Fidelity DNA polymerase, restriction enzymes, CloneJet PCR cloning Kit, Phire^TM^ Plant Direct PCR Master Mix, Gel Extraction Kit, and GeneJET Plasmid Miniprep Kit were purchased from Thermo Fisher Scientific^TM^ (Waltham, MA, USA). Additionally, primers and short synthetic DNA fragments up to 500 bp were synthesized by Integrated DNA Technologies (IDT, Leuven, Belgium) and codon-optimized genes were obtained from Twist Bioscience (South San Francisco, CA, USA). Gibson Assembly^®^ Master mix kits were purchased from New England Biolabs Inc. (Frankfurt am Main, Germany). Detailed lists of the synthetic genes and primers are provided as Supplementary Information (Supplementary Tables S1 and S2).

*E. coli* was cultivated in LB medium (Lennox) purchased from Carl Roth GmbH & Co. KG (Karlsruhe, Germany).

*Pichia pastoris* pre-cultures were cultivated in YPD medium containing 20 g/L peptone, 10 g/L yeast extract, and 20 g/L glucose. *P. pastoris* cultivations for fatty acid production were performed in 96-deep well plates (DWPs) containing 0.5 mL nitrogen-limited, buffered minimal dextrose medium (BMD11) containing 1.9 g/L ammonium chloride, 3.8 g/L yeast nitrogen base without amino acids, and without ammonium sulfate; 0.1 M potassium phosphate buffer, pH 6; 30 g/L glucose; 0.4 mg/L biotin; and 40 mg/L L-histidine, if required. The DWPs were covered with gas-permeable AeraSeal™ films, purchased from Sigma-Aldrich (Vienna, Austria). The DWPs were inoculated to an OD_600_ of 0.2 from precultures grown for 48 h to a stationary phase in YPD and were shaken at 320 rpm and 28 °C for 72–96 h. For strain selection, the YPD or LB agar plates were supplemented with the respective antibiotics (Zeocin^®^: 50 µg/mL for LB and 100 µg/mL for YPD or ampicillin: 100 µg/mL). The media for the plates were solidified by the addition of agar to 1.5%.

### 2.2. Strains and Synthetic Genes

*E. coli* TOP 10F′ (F′[*lac*Iq, *Tn*10(TetR)] *mcr*A Δ(*mrr*-*hsd*RMS-*mcr*BC) φ80*lac*ZΔM15 Δ*lac*X74 *rec*A1 *ara*D139 Δ(*ara-leu*)7697 *gal*U *gal*K *rps*L(StrR) *end*A1 *nup*G), purchased from Thermo Fisher Scientific (Waltham, MA, USA), was used for all the cloning experiments and propagation of expression vectors. The *P. pastoris* (*Komagataella phaffii*) wild-type strain CBS7435 (NRRL Y-11430 [45,46,47]) was used as the host strain for all the further strain constructions (Table 1).

For heterologous gene expression in *P. pastoris*, codon-optimized variants of the following genes were designed by applying the *P. pastoris* codon usage: ‘*TesA* (*E. coli* thioesterase I without N-terminal signal-peptide, AAC73596.1); *Mt*FatA (acyl-ACP thioesterase from *Macadamia tetraphylla*, EU383030.1); *At*FAT-B (Thioesterase from *Arabidopsis thaliana,* Z36911.1). All the thioesterases were expressed as truncated proteins without their native signal-peptides: *Mm*SCD3 (stearoyl-CoA desaturase from *Mus musculus,* AF272037.1); *Ce*FAT-5 (palmitoyl-CoA desaturase from *Caenorhabditis elegans*, AF260242.1); *Rt*ACL (ATP-citrate lyase from *R. toruloides,* CDR44680.1); *Mm*ME (malic enzyme from *M. musculus* NP_032641.2); *Ca*GDH (NADP-dependent glyceraldehyde-3-phosphate dehydrogenase from *Clostridium acetobutylicum* WP_010966919.1); *Ck*PA (phosphate acetyltransferase from *Clostridium kluyveri* WP_012101779.1); *Bb*PKA (D-fructose 6-phosphate phosphoketolase from *Bifidobacterium breve* ERI86329.1); *Rt*IDH1 (mitochondrial NAD+-specific isocitrate dehydrogenase subunit 1 from *R. toruloides,* AEK32870.1); *Rt*IDH2 (mitochondrial NAD+-specific isocitrate dehydrogenase subunit 2 from *R. toruloides,* AEK32871.1); *h*FAS (fatty acid synthase from *Homo sapiens* AAH63242.1); *Ec*AcpS (holo-acyl-carrier-protein synthase from *E. coli,* WP_219378987.1); *Ba*PPT1 (holo-acyl-carrier-protein synthase from *Corynebacterium* (*Brevibacterium*) *ammoniagenes*, EFG81440.1); *Ba*FAS (fatty-acid synthase from *Corynebacterium ammoniagenes,* CAA46024.1). The sequences are listed in Supplementary Table S1.

### 2.3. Strain Design and Genetic Manipulations

Genomic modifications of *P. pastoris* were generated by CRISPR/Cas9-induced integration of knockout or expression cassettes as previously described [48]. Modifications were introduced iteratively by co-transforming Cas9-gRNA plasmids (pPpT4_pHTX1_hsCa) and linearized donor cassettes, followed by screening for successful integration. The donor cassettes were flanked by approximately 1 kb up- and downstream DNA stretches of the target genes to facilitate genomic integration through double crossover events. The donor cassettes and Cas9 plasmids with novel gRNAs were constructed as follows: individual building blocks for expression cassettes, such as promoters, structural genes, transcription terminators, and homologous regions, were amplified from genomic DNA (*P. pastoris* CBS7435 wild-type or *S. cerevisiae* CEN.PK2–1C) or from synthetic DNA fragments, equipped with 30 bp overhangs between adjacent segments to facilitate in vitro assembly. Then, the PCR fragments were assembled with the linearized pJET1.2 vector of the CloneJet PCR cloning kit in one-pot Gibson assembly^®^ reactions to form plasmids for propagation in *E. coli*. For gene assembly reactions, the Gibson assembly^®^ Master Mix was applied according to the supplier’s protocol. Integration sites for the expression cassettes were selected based on the work of Cai et al. and Liu et al. [49,50] with some modifications. For seamless gene knockouts, donor cassettes were constructed from 1 kb up- and downstream DNA stretches of the target genes, which had also been amplified from the *P. pastoris* genome. These fragments were directly fused in an assembly with the linear pJET1.2 vector. Specific genes (*FAD15*, *PXA1*, *LRO1*, *ARE2*, *ELO100*) were knocked out through Cas9-induced frameshift mutations (indels) without supplying knockout cassettes [48]. Additional information on the assembly of certain constructs is given in Supplementary Method S3.

The pPpT4_pHTX1_hsCas vector, kindly provided by the group of Prof. Anton Glieder (Institute of Molecular Biotechnology, Graz University of Technology, Austria), was modified to target new genetic loci. The N20 sequence and ribozyme loop region were exchanged by newly assembling pPpT4_pHTX1_hsCas from two PCR fragments with compatible overhangs. PCR fragments 1 and 2 were amplified from the original pPpT4_pHTX1_hsCas with the primers Fw_T4pHTX1 and Gibson_Rv_insert_HH, Fw_Gibson_insert20N and Rv_T4pHTX1, respectively, with each PCR fragment representing one half of the vector. Primers Gibson_Rv_insert_HH and Fw_Gibson_insert20N introduced novel HH and N20 sequences in fragment 1 and 2 adjacent to one of the two overlapping regions, and subsequent Gibson assembly resulted in the reconstitution of the vector. A detailed vector map and primer sequences are provided in the supplementary file (Supplementary Figure S1, Table S2). Further details on vector function are given in the work of Weninger et al. [48]. Typically, 2–3 gRNAs per target site were designed using the online tool CRISPOR http://crispor.gi.ucsc.edu/ (accessed on 1 September 2023). The CRISPR target sites (N20 and PAM sequences) are listed in Supplementary Table S3. The donor cassette and pPpT4_pHTX1 assemblies were used to transform electro-competent *E. coli* Top10F′ cells and were selected on LB agar plates supplemented with 100 µg/mL ampicillin and 50 µg/mL Zeocin^®^, respectively.

Electrocompetent *P. pastoris* cells were prepared according to the method described by Lin-Cereghino (2005) [51]. Prior to the transformation of *P. pastoris* cells, the donor cassette DNA fragments were excised from the backbone via either *SmiI*, *Sma*I, or *EcoR*V restriction sites previously introduced by PCR primers, followed by gel purification. The competent cells were co-transformed with 300–500 ng of purified donor cassette DNA and 100 ng of Cas9-gRNA DNA fragments. The transformants were plated on YPD agar supplemented with 100 µg/mL Zeocin^®^ to select for the presence of Cas9-gRNA plasmids. Genomic DNA was isolated from transformants using a rapid protocol [52], and PCRs and Sanger sequencing were performed to confirm successful cassette integration. The Cas9-gRNA plasmids were subsequently eliminated through repeated propagation on non-selective medium to enable further engineering cycles.

### 2.4. Bioreactor Cultivations

Fed-batch fermentations were carried out in Sartorius Biostat CT+ 5 L bioreactors with an initial volume of 3 L. Overnight cultures were grown at 130 rpm and 28 °C for 48 h in 100 mL baffled shake flasks with 10 mL YPD medium. These overnight cultures were then used to inoculate fermentation seed cultures with 300 mL YPD medium in 2 L baffled shake flasks to an OD_600_ of 0.1. The seed cultures were cultivated at 130 rpm and 28 °C to an OD_600_ of 30. Bioreactors with 2.7 L of basal salt medium (BSM) containing 0.17 g/L CaSO_4_ × 2 H_2_O, 2.86 g/L K_2_SO_4_, 0.64 g/L KOH, 2.32 g/L MgSO_4_ × 7 H_2_O, 4.25 g/L H_3_PO_4_, 0.22 g/L NaCl, 33 g/L glucose monohydrate, 10 g/L NH_4_Cl, 4.35 mL/L *Pichia* trace metal solution (PTM1), 80 mg/L L-histidine, and 0.1 g/L antifoam 204 (Sigma-Aldrich, Vienna, Austria) were subsequently inoculated to an OD_600_ of 3. The trace metal solution consisted of 5.0 mL/L H_2_SO_4_ (69%), 5.99 g/L CuSO_4_ × 5 H_2_O, 1.18 g/L KI, 3 g/L MnSO_4_ × H_2_O, 0.2 g NaMoO_4_ × 2 H_2_O, 0.02 g/L H_3_BO_3_, 0.92 g/L CoCl_2_ × 6H_2_O, 42.18 g/L ZnSO_4_ × 7 H_2_O, 65 g/L FeSO_4_ × 7 H_2_O, and 0.2 g/L biotin. The fermentation temperature was set to 28 °C throughout the entire process and the dissolved oxygen (dO_2_) was maintained above 40% saturation with initial agitation and aeration rates of 500 rpm and 1 vvm, respectively; the agitation rate was steadily increased to 800 rpm as oxygen consumption increased. In later stages of the fermentation, the agitation was increased to 1200–1350 rpm to break the foam, which spiked the dO_2_ to ~80%. The pH was measured with an autoclavable pH-electrode (EasyFerm Plus PHI K8 120, Hamilton, Bonaduz, Switzerland) and was maintained at pH 5.6 through the automatic addition of 6 M NaOH. Dissolved oxygen was monitored with a dO_2_ sensor (InPro6850i/12/120, Mettler Toledo, Vienna, Austria). Foaming was controlled by the addition of 10% antifoam 204 dissolved in 20% ethanol in water (*w*/*v*) when needed. All the sensors were calibrated before fermentation, and the addition of solutions (base, antifoam, feeding solutions) and the aeration rate were controlled by a Biostat CT+ controlling unit.

### 2.5. Fed-Batch Feeding

After an initial batch phase of 14 h, the bioreactors were fed with solution 1 (500 g/L glucose, 12 mL/L PTM1, and 80 mg/L L-Histidine) and solution 2 (250 g/L NH_4_Cl) at rates of 2–8 g/L/h and 1–4 g/L/h, respectively, maintaining glucose levels between 1 and 10 g/L. As a result, 183 g/L (203 g/L total) glucose and 26 g/L (36 g/L total) ammonium chloride were fed until the end of the fermentation (134 h feeding period, 148 h total batch and fed batch). The cell wet weight (CWW) was measured by centrifuging one mL of the cell suspension for 1 min in pre-weighed microcentrifuge tubes at 4500× *g*, followed by removal of the supernatant and determination of the wet cell weight. Aliquots of the supernatant and pellet fractions were subjected to fatty acid extraction and subsequently analyzed by GC-MS or GC-FID.

### 2.6. Fatty Acid Methyl Ester (FAME) Analysis—Sample Preparation

Free fatty acids were methylated through acid-catalyzed transesterification with methanol and hydrochloric acid, followed by quantification of FAMEs using either GC-MS or GC-FID. For sample preparation, 0.2 mL of vigorously mixed suspension from bioreactor or deep-well plate cultivations was centrifuged for 1 min at 4500× *g*. The resulting supernatant and pellet (dissolved in 0.2 mL pure H_2_O) were transferred into 10 mL Pyrex glass tubes with Teflon-lined plastic caps. These samples were then frozen at −80 °C and lyophilized overnight using an Alpha 1–4 LDplus freeze dryer (Martin Christ, Osterode am Harz, Germany).

To produce the FAMEs, 0.5 mL of (5%) hydrochloric methanol containing 0.1 mg/mL pentadecanoic acid as an internal standard were added to each tube. After vortexing briefly, the tubes were incubated at 85 °C for 1.5 h. Next, 0.5 mL of a 0.8% KCl solution and 1 mL of hexane were added, and the FAMEs were extracted into the hexane layer by shaking at 1500 rpm on a Vibrax^®^ mixer (Staunfen, Germany). Following a brief centrifugation step, 200 µL of the upper hexane layer were transferred to GC vials with glass inlets for subsequent GC analysis.

### 2.7. GC Analysis

The FAMEs were quantified using either a Shimadzu GC-2010 Plus device with a Shimadzu GCMS-QP2010 SE detector (GC-MS) or with a flame ionization detector FID-2010 Plus (GC-FID). Both devices were equipped with a ZB-5MSi column (5%-phenyl 95%-dimethylpolysiloxane phase 30 m × 0.25 mm × 0.25 µm) and operated with helium and nitrogen as carrier gases, respectively. For the GC-MS analysis, the temperature program started with an initial hold at 100 °C for 1 min, followed by a ramp to 300 °C with a linear increase of 15 °C/min and a final hold of 1 min. The injection, ion source, and interface temperatures for GC-MS were set to 240 °C, 250 °C, and 300 °C, respectively. For the GC-FID analysis, the temperature program began with an initial 1 min hold at 150 °C, followed by a ramp to 340 °C with a linear increase of 15 °C/min and a final hold of 1 min. Injection and FID temperatures of 240 °C and 320 °C were used for the GC-FID measurements. The flow rates for GC-MS and GC-FID were set to 0.92 mL/min (linear velocity 35.7 cm/s) and 0.81 mL/min (linear velocity 19.6 cm/s), respectively. Both devices utilized an injection volume of 1 µL with split ratios of 30 (GC-MS) and 20 (GC-FID). The described GC methods facilitated rapid sampling but did not enable clear separation of C18:1, C18:2, and C18:3 fatty acids. Consequently, the values for these fatty acids (C18:1, C18:2, and C18:3) were combined into a single peak for most of the analyzed strains. Selected strains were analyzed with an extended GC method that allowed for clear separation of all the peaks (Method S1).

### 2.8. Glucose and Ethanol Concentration Analysis

The glucose and ethanol concentrations were monitored using HPLC measurements. Fermentation samples were centrifuged for 2 min at 16,000× *g*, filtered through 0.45 µm syringe filters, and diluted if necessary. An analysis was performed on a Merck-Hitachi LaChrome HPLC System (Merck, Darmstadt, Germany) with a Bio-Rad Aminex HPX-87H column (Bio-Rad, Hercules, CA, USA) (precolumn: Micro-Guard Cation H-Cartridge 125-0129) equipped with a Merck LaChrome L-7490 refractive index detector. The samples were eluted with 5 mM sulfuric acid at a flow rate of 0.6 mL/min and a constant temperature of 65 °C.

## 3. Results and Discussion

**Figure 1:**
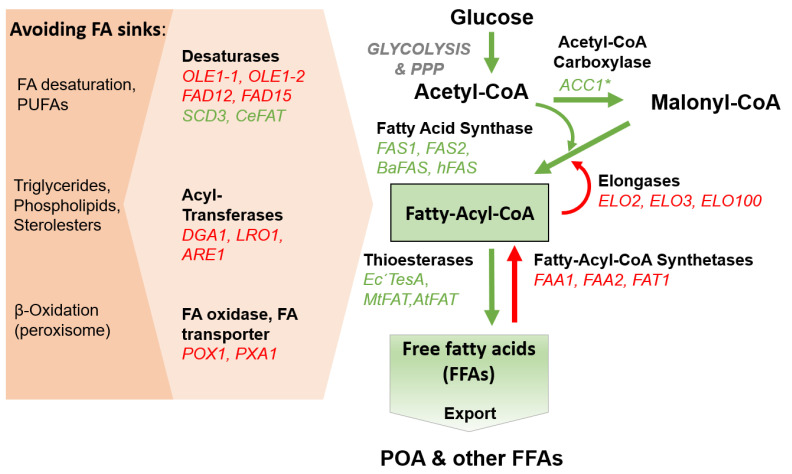
Simplified fatty acid metabolism in *P. pastoris* and strategies for FFA overproduction. In red: enzymes in *P. pastoris* to be knocked out to facilitate fatty acid production. In green: heterologous or endogenous genes overexpressed or modified in *P. pastoris*. Red arrows: enzymatic reactions that are unfavorable for FFA production. Green arrows: enzymatic reactions that facilitate FFA production. *OLE1-1/OLE1-2*, ∆9-desaturase; *FAD12*, ∆12-desaturase; *FAD15*, ∆15-desaturase; *SCD3*, stearoyl-CoA desaturase from *M. musculus*; *DGA1*, diacylglycerol-acyltransferase; *LRO1*, phospholipid:diacylglycerol-acyltransferase; *ARE1*, acyl-CoA:sterol-acyltransferase; *POX1*, fatty-acyl coenzyme A oxidase; *PXA1*, peroxisomal long-chain FA importer; *FAS1*, fatty acid synthase subunit β; *FAS2*, fatty acid synthase subunit α; *BaFAS*, fatty acid synthase from *C. ammoniagenes*; *Ec*‘*TesA*, thioesterase A from *E. coli* without leader peptide sequence; *MtFAT*, thioesterase from *M. tetraphylla*; *AtFAT*, thioesterase from *A. thaliana*; *ELO*, elongases; ACC1*, acetyl-CoA carboxylase S1151A mutant; FAA1, FAA2, fatty acyl-CoA synthetases; *FAT1*, very long fatty acyl-CoA synthetases and fatty acid importer.

Our primary objective was to develop a *P. pastoris* strain capable of efficiently producing and secreting free fatty acids, especially palmitoleic acid (POA), using glucose as the carbon source. The efficient secretion of fatty acids is of particular significance, as it streamlines downstream processing, which often constitutes a substantial portion of the production costs in biotechnological applications [53]. To achieve fatty acid secretion, we implemented the following engineering steps: (i) deletion of acyl-CoA synthetases, (ii) overexpression of heterologous thioesterases, (iii) enhancement of precursor supply, and (iv) alleviation of competing pathways in accordance with the push–pull block theorem commonly employed in metabolic engineering [54]. Additionally, we aimed to shift the fatty acid profile towards POA to achieve higher product titers in the supernatant by expressing heterologous fatty acid desaturases and fatty acid synthases in combination with engineering of the endogenous fatty acid elongation system. Figure 1 summarizes the general fatty acid metabolism in *P. pastoris* and highlights our engineering targets.

### 3.1. Establishing Free Fatty Acid Secretion in P. pastoris

**Figure 2:**
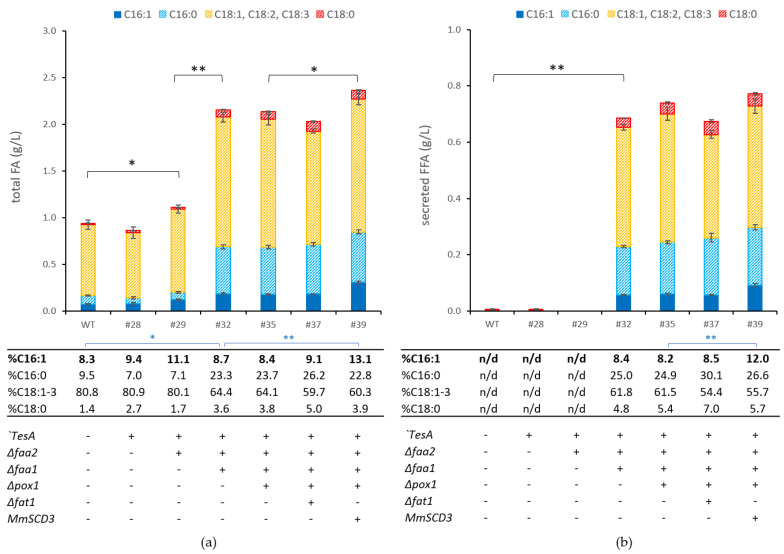
Fatty acid analysis of engineered *P. pastoris* strains: establishing a platform strain for fatty acid secretion. FFAs were extracted from *P. pastoris* cell culture or supernatant after cultivation in DWPs with BMD11 medium for 96 h at 320 rpm and 28 °C. (**a**) total FAs (g/L cell culture; obtained from supernatant and pellet); (**b**) amount of FFAs (g/L cell culture) secreted in the medium. The table presents the percentage of individual fatty acid species produced by the engineered strains. The numbers given (#) refer to the *P. pastoris* strains listed in Table 1. WT, *P. pastoris* wild-type strain. All data are presented as mean ± SD of biological triplicates. Statistical analysis was performed using two-sample two-tailed Student’s *t*-test (* *p* value < 0.05, ** *p* value < 0.01; black asterisks indicate analysis of total or secreted fatty acids; blue asterisks indicate analysis of C16:1 content). Minor fatty acid species (C17:1, C17:0, and very long chain fatty acids, sum amounting to ≤3% of total fatty acids) were not included in this analysis.

Fungal fatty acid synthesis de novo is catalyzed by a cytosolic type I FAS complex, typically composed of two polypeptides: FAS1 and FAS2. Acyl-chain elongation occurs iteratively, adding two carbon atoms at a time until the final chain length of 16 to 18 carbon atoms is reached [55]. Each cycle consumes one molecule of malonyl-CoA and two molecules of NADPH. Malonyl-CoA, the primary building block in FA synthesis, is produced through the reaction catalyzed by the cytosolic acetyl-CoA carboxylase (ACC1) from acetyl-CoA [55]. The key activities in these processes are modulated by feedback inhibition from long-chain fatty-acyl-CoAs [56], the major products of the FA synthase complex. The release of FFAs from the acyl-CoA pool, i.e., via heterologous cytosolic thioesterase expression, partially alleviates this feedback inhibition [57]. However acyl-CoA synthetases, encoded by *FAA* genes, readily counteract this reaction by reactivating released FFAs, thereby preventing overflow metabolism [58]. In *FAA* null mutants, acyl-CoA pools are continuously depleted through cellular processes, leading to a relief of feedback inhibition on FA synthesis, resulting in an overproduction phenotype that secretes FFAs [59]. To enable this effect in *P. pastoris*, we performed sequential deletions of the acyl-CoA synthetase genes *FAA2* and *FAA1*, the two *FAA* variants present in *P. pastoris*, while simultaneously overexpressing a leaderless *E. coli* thioesterase ‘*TesA* [60] from the strong constitutive P_GAP_ promoter. It was previously shown that a similar engineering approach implemented in *S. cerevisiae* enabled free fatty acid overproduction, reaching FFA titers of up to 0.67 g/L in shake flask cultivation media [58,61]. In our study, the overexpression of ‘*TesA* in the ∆*faa2* strain background (strain *Pp*#29) resulted in a slight increase in the intracellular FA content; however, no considerable amounts of FFAs were secreted into the medium (Figure 2). The FFA secretion phenotype was achieved by additionally deleting the *FAA1* gene in strain *Pp*#29 (Δ*faa2* ‘*TesA*), leading to a 2.3-fold increase in the total cellular FA content compared to the wild-type strain (Figure 2). The resulting strain *Pp*#32 secreted 0.69 g/L of FFAs in the DWP cultivations. These findings are consistent with a recent study performed with *P. pastoris* GS115 [62], where the ∆*faa1* knockout yielded the most significant improvement, while the implementation of ∆*faa2* caused a minor but still noticeable increase in the FA content. Notably, strain *Pp*#32 displayed reduced biomass yield compared to the wild-type strain (CWWs are listed in Supplementary Table S4), which may be attributed to the metabolic burden induced by FFA production. To further enhance FA production, we deleted the fatty acyl-CoA oxidase 1 gene (*POX1*), encoding the first step in β-oxidation [63], to prevent the degradation of newly synthesized fatty acids. This strategy was successfully implemented in an FFA-producing *E. coli* strain *(*deletion of the *POX1* homolog FadE), which led to 4-fold increase in productivity [57]. We additionally targeted the fatty acid importer Fat1p, which is responsible for FA uptake and exerts minor acyl-CoA synthetase activity [59,64]. The deletion of *FAT1* in an *S. cerevisiae FAA* quadruple mutant was shown to promote elevated internal FFA accumulation [59]. Surprisingly, both sequential deletions (Strains *Pp*#35 and *Pp*#37) did not result in a significant increase in productivity compared to the background strain *Pp*#32 (Figure 2). The negligible change in productivity facilitated by the *POX1* deletion is consistent with findings reported in prior research [62]. Nevertheless, we retained the deletion of *POX1* in subsequent strains, as it may hold potential for synergistic interactions with other modifications.

### 3.2. Engineering the Fatty Acid Desaturase System in P. pastoris

The ∆9-desaturase Ole1p in *S. cerevisiae* plays a crucial role in converting unsaturated FAs to their monounsaturated counterparts, including the synthesis of palmitoleic acid [65]. In contrast to *S. cerevisiae*, *P. pastoris* CBS7435 possesses two distinct ∆9-desaturase isoforms, Ole1-1p and Ole1-2p [47].

Initial experiments indicated that Ole1-1p exhibits major ∆9-desaturase activity and that ∆*ole1-1* mutants (wild-type background) were unable to grow without supplementation of exogenous fatty acids. Subsequent attempts to exchange *OLE1-1* for a C16-specific ∆9-desaturease from *C. elegans* (*CeFAT*) [66] were unsuccessful. In the case of *S. cerevisiae*, ∆*ole1* mutants also require FA supplementation. However, the replacement of *OLE1* with a rat ∆9-desaturase yielded viable cells [65], which differs from our observations in *P. pastoris*. Interestingly, unlike the ∆*ole1-1 P. pastoris* strains, ∆*ole1-2* mutants can thrive without the need for FA supplementation and exhibit modestly increased C16:1 content. Consequently, we retained this modification in all the subsequent strains.

Having established FFA secretion in strain *Pp*#35 (Figure 2), we set out to assess whether the overexpression of additional ∆9-desaturases could increase the POA content or overall FFA productivity.

**Figure 3:**
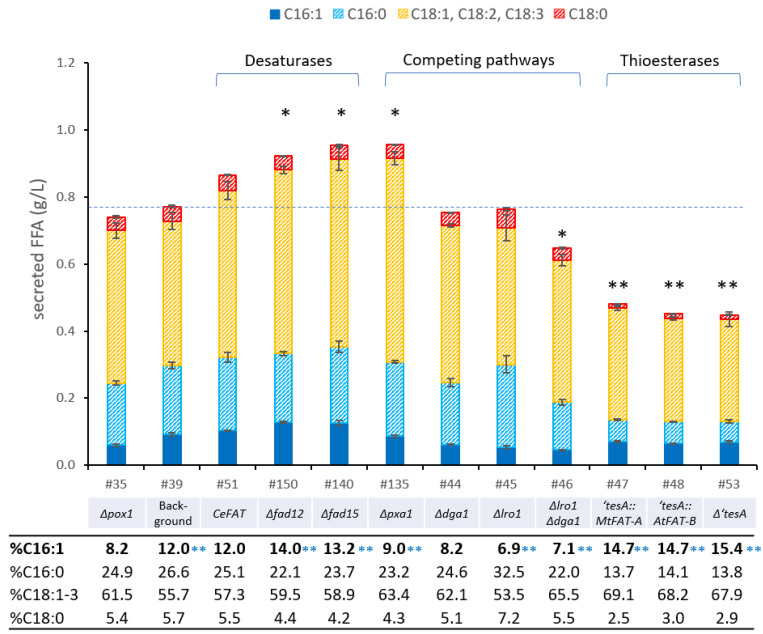
Fatty acid analysis of engineered *P. pastoris* strains: engineering fatty acid desaturase system, alleviating competing pathways, and expression of heterologous thioesterases. FFAs were extracted from cell culture supernatant after cultivation in DWPs with BMD11 medium for 96 h at 320 rpm and 28 °C. The numbers given refer to the numbered *P. pastoris* strains listed in Table 1 with respective modifications compared to the background strains *Pp*#35 (background for strains *Pp*#44–46) and *Pp#*39 (background for all other strains) listed below. The table presents the percentage of individual fatty acid species produced by the different engineered strains. All data are presented as mean ± SD of biological triplicates. Statistical analysis was performed using two-sample two-tailed Student’s *t* test (comparison to respective background strain; * *p* value < 0.05, ** *p* value < 0.01; black asterisks indicate analysis of total secreted fatty acids; blue asterisks indicate analysis of C16:1 content). Minor fatty acid species (C17:1, C17:0, and very long chain fatty acids, sum amounting to ≤3% of total fatty acids) were not included in this analysis.

Previous studies have demonstrated that overexpression of *OLE1* in engineered *S. cerevisiae* leads to increased fatty alcohol production [54]. Furthermore, increased ∆9-desaturase activity has been associated with lipid accumulation phenotypes in *Y. lipolytica* and mammalian tissue [67]. This effect is a result of reduced feedback inhibition of the FA biosynthesis machinery by unsaturated fatty-acyl-CoAs [67]. Given that *P. pastoris* exhibits low palmitoleic acid (C16:1) content and high levels of oleic acid (C18:1) compared to other yeast species (Table 2), we devised a strategy to overexpress a heterologous ∆9-desaturase, *SCD3* from *M. musculus*, known for its high specificity for palmitoyl-CoA [68]. The strong constitutive promoter P_TEF1_ (translation elongation factor 1) was used to foster *MmSCD3* expression (strain *Pp*#39). The heterologous expression of *MmSCD3* did not enhance the overall fatty acid secretion significantly but it did raise the POA content from 8.3% to 13.1% (Figure 2). Consequently, we selected *Pp*#39 as the basis strain for further engineering efforts, like the introduction of *CeFAT,* another palmitoyl-CoA-specific ∆9-desaturase. In contrast to the expression of *CeFAT* in the basic Δ*ole1-2* background (*Pp*#16), which exhibited minor changes in the FA content or profile, the overexpression of *CeFAT* from the glycolytic *PGK1* promoter in the *Pp*#39 strain increased the titer of secreted fatty acids from 0.77 g/L to 0.86 g/L but could not enhance the POA content (Figure 3). Further attempts to overexpress a third heterologous desaturase from *S. cerevisiae* in later experiments did not result in any improvements in the productivity or POA content (data not shown).

Yeast ∆9-desaturases contain a self-sufficient cytochrome b5 domain that provides electrons for the desaturase complex, transferring electrons from NADH directly to the desaturase via a cytochrome b5 reductase, which facilitates efficient coupling of the cofactor supply and double bond formation [65]. However, this domain is not present in higher eukaryotic counterparts, so we hypothesized that electron transfer might be limiting in our engineered strains. To this end, we introduced additional copies of cytochrome b5 and NADH-dependent cytochrome b5 reductase genes, either as endogenous variants (*Pp*#127) or codon-optimized genes from *M. musculus* (*Pp*#129), linked to the Mm*SCD3* coding strand via T2A sequences [69]. Unlike a previous study with *S. cerevisiae* overexpressing FA desaturases from *Kluyveromyces lactis* [70], the *CYB5* overexpression proved to be unsuccessful in *P. pastoris* (*Pp*#39) overexpressing *Mm*SCD3 (data not shown).

Aside from ∆9-desaturases, *P. pastoris* possesses ∆12- and ∆15-desaturase activities, as confirmed by a previous analysis of the wild-type fatty acid profile (Table 2), which revealed the presence of linoleic acid (C18:2) and linolenic acid (C18:3). The responsible desaturases, encoded by *FAD12* and *FAD15 (FAD-3)*, were previously identified in *P. pastoris* GS115 and characterized through heterologous expression in *S. cerevisiae* [71,72]. Similarly, we confirmed Fad12p as the sole ∆12-desaturase in the *P. pastoris* CBS7435 wild-type strain [71], as evidenced by the absence of C18:2 in a *FAD12* null mutant (Supplementary Figure S2). To explore the impact of ∆12- and ∆15-desaturases on the FA profile and secretion, we individually deleted *FAD12 (*strain *Pp*#150) and *FAD15* (strain *Pp*#140), in our platform strain *Pp*#39. Surprisingly, these deletions increased the FA secretion by 19% and 24%, respectively, and led to modest increases in the POA content (Figure 3). Notably, the biomass yield for strain *Pp*#150 was compromised (Supplementary Table S4). We hypothesize that the increased productivity induced by the deletions of *FAD12* or *FAD15* is caused by a compensation effect, where a lack of membrane fluidity arising from a deficiency or reduction in PUFA is bolstered by enhanced monounsaturated acid synthesis. This should lead to a reduction in saturated acyl-CoAs, which otherwise inhibit FA synthesis. Indeed, we observed a marginal reduction in saturated fatty acids in these strains (Figure 3).

### 3.3. Analysis of Growth Conditions and Nutrient Manipulation

In addition to genetic factors, the fatty acid composition and secretory capacity of various yeast species are influenced by nutrient availability. Nutrient deficiencies, such as nitrogen or phosphate limitations, coupled with high carbon abundance have been shown to trigger lipid accumulation in oleaginous yeast and certain conventional yeast species [73,74]. Furthermore, oxidative stress can induce changes in the unsaturated fatty acid content as a protective response against ROS damage [75]. Although several biotechnologically relevant yeast species have been investigated for POA synthesis under various cultivation conditions, *P. pastoris* was not among the organisms studied [74]. Consequently, we conducted experiments to assess the FA production capacity and fatty acid composition of strain *Pp*#39 under different carbon-to-nitrogen ratios and nitrogen sources, using glucose as the carbon source, in batch cultivation format (96-DWPs; media compositions are given in Supplementary Table S5). We found that among the different N-sources (ammonium chloride, ammonium sulfate, yeast extract, and peptone), peptone yielded the highest FA secretory capacity (up to 2-fold higher than most other N-sources) and that the peptone or yeast extract led to the highest POA content of up to 16–18%, whereas other N-sources yielded 12–14% POA (Supplementary Figure S3). Notably, the use of peptone or yeast extract in the large-scale production of comparably low-priced compounds is not feasible. Hence, we chose ammonium chloride as the nitrogen source for further experiments. When considering nitrogen limitation, CN ratios ranging from 5 to 50 led to an FA secretory capacity that was similar to the value obtained for BMD11, whereas CN ratios above 50 resulted in compromised FA secretion, probably due to the reduced biomass yields as a consequence of low nitrogen availability. Interestingly, the increased CN ratios with ammonium sulfate and peptone as a nitrogen source led to a relatively increased C16:1 content, yet the overall FA titers were low. The minor impact of the CN ratio on lipid accumulation in *P. pastoris* may be attributed to the fact that *P. pastoris* does not possess ATP-citrate lyase [76] or cytosolic malic enzymes, which catalyze key reactions required for lipid accumulation in oleaginous yeasts [77]. We decided to set the CN ratio for subsequent experiments to ~25 to balance the biomass yield and fatty acid production. Lastly, it is important to note that these findings simulate batch cultivations and that optimal parameters for fed batch and continuous formats in bioreactors may differ from these conditions, as constant CN rations can only be consistently maintained in such controlled feeding setups.

### 3.4. Down-Regulation of Competing Pathways

Further strain construction efforts involved the elimination of pathways in strain *Pp#*39 (or *Pp*#35 for the interference of neutral lipid storage) that consume fatty acyl-CoAs (see Figure 1). In previous studies, deleting the peroxisomal long-chain fatty acid importer *PXA1* in an *S. cerevisiae* ∆*pox1* ∆*faa1* strain resulted in increased FFA production by completely abolishing β-oxidation [78]. Pursuing the same strategy, strain *Pp*#135 improved FA secretion by 24% but, astonishingly, displayed slightly reduced POA content (Figure 3). The exact reason for this reduction remains unclear, as FA transport across membranes is still—at least partially—obscure [79].

The reduction of FA flux to neutral lipids was achieved by deleting the major genes involved in neutral lipid synthesis, *DGA1* and *LRO1* (diacylglycerol acyl-transferases), as well as *ARE2*, the sterol acyl-transferase, which are responsible for catalyzing acyl transfer to diacylglycerides and sterols, respectively. Individual deletions of *LRO1* and *DGA1*, respectively, led to negligible changes in FFA secretion (increase from 0.74 g/L to 0.75 g/L and 0.76 g/L secreted FFA, respectively), but were accompanied by a reduction in stored neutral lipids (Supplementary Figure S4). The combinatorial strain *Pp*#51a (∆*dga1* ∆*lro1*∆*are2*) showed impaired growth and a significant reduction in productivity, suggesting a crucial role of neutral lipid storage for strain fitness. Thin layer chromatography conducted with lipid extracts from strains *Pp*#35, *Pp*#44, *Pp*#46, and *Pp*#51a confirmed the reduction or absence of triglycerides and sterol esters in the respective strains (Supplementary Figure S5). In later experiments, we pursued an alternative strategy that involved overexpression of *DGA1* and triacylglycerol lipases 3 and 4 (*TGL3*, *TGL4*) to divert FA flux through lipid bodies (see Section 3.6).

### 3.5. Expression of Heterologous Thioesterases

As previously mentioned, thioesterases play a crucial role in FFA biosynthesis, governing, among other reactions, the profile of released FFAs. Substrate specificities vary among thioesterases, with certain variants accepting a broad range of long chain acyl-CoAs, while others only accept a few or are highly specific towards single activated FA species [80]. Plenty of length-specific enzymes have been identified in plants, where thioesterases terminate plastidal fatty acid synthesis, rendering the fatty acid composition of plant kernel fats [81,82].

To increase the POA content in platform strain *Pp*#39, we overexpressed two plant thioesterases, namely, *MtFAT-A* from *M. tetraphylla* and *AtFAT-B* from *A. thaliana*, which have been shown to exhibit high specificity for C16:1 and C16:0 [81,82]. The *FAT* genes were integrated into the *HIS4* locus, replacing ‘*TesA* to verify the individual thioesterase activity. The N-terminal leader peptides for translocation to plastids in their original hosts were omitted in the *P. pastoris* expression constructs to allow for cytosolic expression. Surprisingly, the substitution of ‘*TesA* with *MtFAT-A* or *AtFAT-B* resulted in a reduction in the FA titers by approximately 42% and led to alterations in the fatty acid profile (Figure 3). The resulting strains mirrored the FA composition and productivity of strain *Pp#*53 with a ‘*TesA* knockout in the same background. This indicates that the plant thioesterases were probably not active in this context and the changes in FA content were solely due to the ‘*TesA* removal. The poor activity of the plant thioesterases may be attributed to insufficient protein processing or low substrate acceptance. The latter may be particularly true since plastidal thioesterases hydrolyze acyl-ACP thioesters in their original host, while fatty acyl chains in yeast are attached to CoA. However, previous studies have shown that thioesterases from different origins can accept both substrate types [83]. Despite the absence of heterologous thioesterase activity in strain *Pp*#53, it still secreted 0.45 g/L of FFAs, with marginally elevated POA content and reduced C16:0 levels, potentially due to endogenous TE or lipase activities (Figure 3). A sequence similarity search [84] (BLAST) for thioesterases in the genome of *P. pastoris* CBS7435 only identified a homolog of the *S. cerevisiae* peroxisomal thioesterase, which is likely involved in β-oxidation [85]. Consequently, the release of FFAs in this strain is presumably a result of lipase activity.

### 3.6. Enhancing the Availablity of Fatty Acid Precursor Molecules

**Figure 4:**
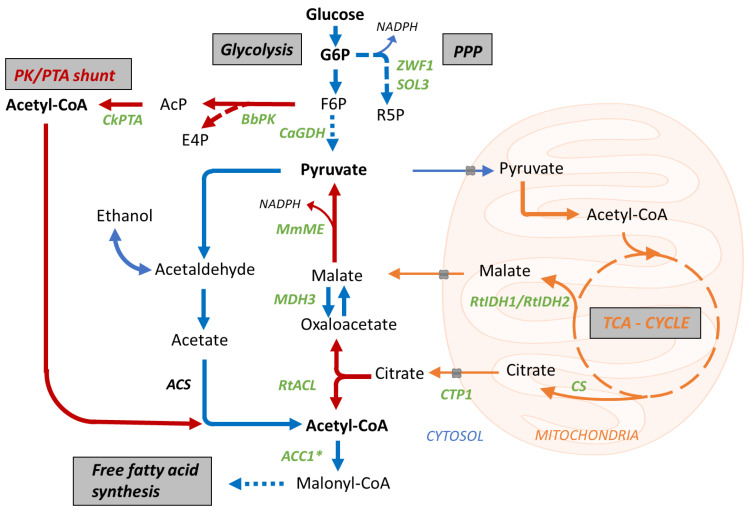
Engineering of fatty acid precursor synthesis (from glucose to malonyl-CoA). Red arrows indicate heterologous reactions; blue arrows indicate endogenous reactions; green: genes to be overexpressed to enhance precursor abundance. Red arrows indicate heterologous enzymatic reactions that enhance precursor synthesis. Blue arrows and orange arrows indicate endogenous cytosolic and mitochondrial reactions, respectively, required for fatty acid precursor synthesis. *ZWF1*, glucose-6-phosphate dehydrogenase; *SOL3*, 6-phosphogluconolactonase; *CaGDH*, N*AD*P+-dependent glyceraldehyde-3-phosphate dehydrogenase from *C. acetobutylicum*; *BbPK*, phosphoketolase of *B. breve*; *CkPTA*, phosphotransacetylase of *C. kluyveri*; *MmME*, malic enzyme of *M. musculus*; *MDH3*, malate dehydrogenase; *RtACL*, ATP citrate lyase of *R. toruloides*; ACC1*, acetyl-carboxylase 1 variant S1151A; *CTP1*, mitochondrial citrate exporter; *CS*, citrate synthase; *RtIDH1/IDH2*, mitochondrial NAD+-specific isocitrate dehydrogenase subunits 1 and 2 from *R. toruloides*.

**Figure 5:**
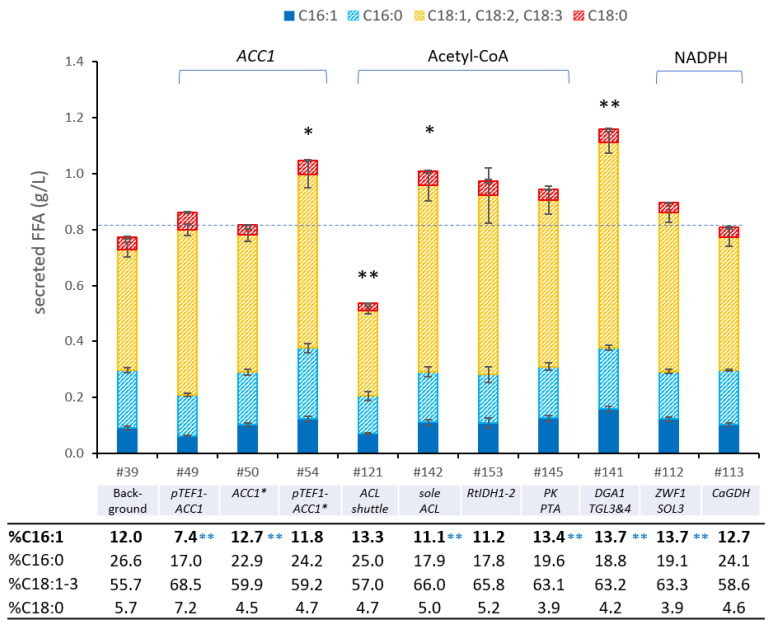
Fatty acid analysis of engineered *P. pastoris* strains: enhancing supply of precursors malonyl-CoA, acetyl-CoA, and redox cofactor NADPH. FFAs were extracted from cell culture supernatant after cultivation in DWPs with BMD11 medium for 96 h at 320 rpm and 28 °C. The numbers given (#) refer to the *P. pastoris* strains listed in Table 1 with modifications compared to their respective background strains: *Pp*#39 (background for *Pp*#49–54), *Pp*#50 (background for *Pp*#112–145), and *Pp*#142 (background for *Pp*#153) listed below. ACC1* denotes *ACC1*^S1151A^. The table presents the percentage of individual fatty acid species produced by the different engineered strains. All data are presented as mean ± SD of biological triplicates. Statistical analysis was performed using two-sample two-tailed Student’s *t* test (comparison to respective background strain; * *p* value < 0.05, ** *p* value < 0.01; black asterisks indicate analysis of total secreted fatty acids; blue asterisks indicate analysis of C16:1 content). Minor fatty acid species (i.e., C17:1, C17:0, and very long chain fatty acids, sum amounting to ≤3% of total fatty acids) were not included in this analysis.

To push the flux towards FA production, we aimed to increase the supply of the FA precursors acetyl-CoA and malonyl-CoA (Figure 4). It is commonly reported that the carboxylation of acetyl-CoA to malonyl-CoA, catalyzed by the acetyl-CoA carboxylase (Acc1p), poses a major bottleneck for FA synthesis in yeast [54,86]. Acc1p activity is regulated at the transcriptional and post-translational levels. Previous studies of *S. cerevisiae* demonstrated that the overexpression of *ACC1*, along with mutagenesis of *SNF1* regulation sites, led to a 3-fold increase in Acc1p activity [86]. Snf1p phosphorylation sites, identified as Serine 659 and Serine 1157 in Acc1p of *S. cerevisiae*, were conserved in the *P. pastoris* variant as Serine 693 (LRTPSPGKL) and Serine 1151 (MDRAVSVSDL). To boost the Acc1p activity in *Pp*#39, we introduced the S1151A substitution within the genomic *ACC1* gene and replaced its native promoter region with the constitutive P*_TEF1_* promoter. Individual modifications, like promoter exchange *Pp*P*_ACC1_*::P*_TEF_*_-*ACC1*_ (strain *Pp*#49) or deletion of the phosphorylation site *PpACC1*::*ACC1*^S1151A^ (ACC1*; strain *Pp*#50), improved productivity by 11% and 6%, respectively, whereas the combination of both strategies increased the titers by 36% (Figure 5). Interestingly, strain *Pp*#50 (ACC1^S1151A^) displayed slightly elevated POA content, whilst strain *Pp*#49 (*Pp*P*_ACC1_*::P_TEF-*ACC1*_) produced fewer C16 fatty acids. Early studies on fungal fatty acid synthases revealed that increased Acc1p activity promotes C18 synthesis by the FAS complex, as higher malonyl-CoA concentrations facilitate efficient malonyl loading rather than chain termination [87]. This pattern aligns with the results observed for strain *Pp*#49 but does not explain the C16:1 increase in strain *Pp*#50.

After enhancing the Acc1p activity, we aimed to increase the cytosolic acetyl-CoA abundance. In most Saccharomycotina, including *S. cerevisiae* and *P. pastoris*, cytosolic acetyl-CoA is primarily derived from pyruvate, which undergoes conversions to acetaldehyde and acetate before forming acetyl-CoA [76]. In contrast, oleaginous yeasts adopt a different approach for cytosolic acetyl-CoA synthesis. They redirect the carbon flux through the ATP citrate lyase (*ACL*) pathway by exporting citrate from the mitochondria and converting it to acetyl-CoA and oxaloacetate. This metabolic rearrangement efficiently diverts the carbon flux from the TCA cycle to FA synthesis in oleaginous yeasts [88]. *P. pastoris* lacks endogenous genes for *ACL* and cytosolic malic enzyme (*ME*; present only in mitochondria). To overcome this limitation, we integrated a heterologous *ACL* shuttle construct in strain *Pp*#50 composed of *R. toruloides ACL*, *M. musculus* malic enzyme (*ME*), endogenous malate dehydrogenase (*MDH3*), and a mitochondrial citrate exporter (*CTP1*). While this strategy was previously successful in an FA-producing *S. cerevisiae* strain [61], in strain *Pp*#50, it resulted in reduced FFA titers (from 0.82 g/L to 0.53 g/L) and lower biomass yields (Figure 5), suggesting a metabolic imbalance likely caused by the withdrawal of citrate from the TCA cycle or an imbalance at the pyruvate node. However, interestingly, recent research has demonstrated that overexpression of a single *ACL* gene (without *ME*) could enhance FFA production from methanol by 23% in an engineered *P. pastoris* strain [62]. Building on this knowledge, we introduced the *R. toruloides ACL* (*RtACL*) into the *Pp*#50 background, which raised the secreted FFA titers from 0.82 g/L to 1 g/L (Figure 5).

In oleaginous yeasts, nitrogen depletion triggers the redirection of the citrate flux from the TCA cycle to the *ACL* shunt, caused by the assimilation of adenosine monophosphate and the subsequent inactivation of isocitrate dehydrogenase (Idhp) [77]. To mimic this effect in *P. pastoris*, we aimed to replace the endogenous *IDH1* and *IDH2* genes with the variants from *R. toruloides*. Notably, the gene replacements were not successful, and we identified that the heterologous *IDH* genes were randomly integrated in the genome, suggesting an essential role of the endogenous *IDH* variants. The resulting strain displayed reduced fatty acid titers (Figure 5, *Pp*#153), presumably due to the perturbation of the TCA cycle.

We then explored another strategy for cytosolic acetyl-CoA synthesis, involving the expression of a phosphoketolase (*PK*) and phosphotransacetylase (*PTA*) shunt. This pathway splits fructose-6-phosphate into erythrose-4-phosphate and acetyl-phosphate, followed by the synthesis of acetyl-CoA [89] and was successfully utilized to raise the FFA production from 0.6 g/L to 0.7 g/L in a modified *P. pastoris* strain [62]. The co-expression of *B. breve PK* and *C. kluyveri PTA* in *P. pastoris* strain *Pp*#50 increased the FA production by 15%, yielding 0.94 g/L FFAs in *Pp*#145 (Figure 5).

Next, we aimed to divert the FA flux through the lipid bodies by overexpressing endogenous diacylglycerol acyltransferase *DGA1* and triacylglycerol lipases *TGL3* and *TGL4* to increase the neutral lipid synthesis, leading to continuous fatty acid release from stored TAGs. Indeed, channeling the fatty acids through the lipid bodies increased the secreted fatty acid titers from 0.82 g/L (*Pp*#50) to 1.16 g/L (*Pp*#141), representing a 42% increase.

Subsequently, we focused on increasing NADPH availability for fatty acid synthesis. In yeast, NADPH is generated in the pentose phosphate pathway (PPP) [90] during the oxidation of acetaldehyde to acetate via an NADP+-dependent aldehyde dehydrogenase [54] or, if present, in the reaction of a cytosolic malic enzyme [88]. Since incorporating the latter reaction in an *ACL* shuttle reduced productivity in our engineered strains, we attempted to increase the flux through the PPP. Glucose-6-phosphate dehydrogenase (Zwf1p) and 6-phosphogluconolactonase (Sol3p), two enzymes that were previously shown to limit PPP flux in *P. pastoris* [91], were overexpressed from the constitutive bidirectional histone promoter P*_HHX1_* [92]. These modifications raised the fatty acid titers from 0.82 g/L in *Pp*#50 to 0.89 g/L in *Pp*#112 (Figure 5). Since yeast inherently exhibits high flux through glycolysis, we also tested the expression of an NADP+-dependent glyceraldehyde-3-phosphate dehydrogenase (*CaGDH*), aiming to reduce glycolytic NADH synthesis and instead produce NADPH, which had been successfully conducted in *Y. lipolytica* [90]. However, pursuing this approach in strain *Pp*#50 did not result in noticeable changes in FFA productivity (see strain *Pp*#113, Figure 5).

### 3.7. Expression of Heterologous Fatty Acid Synthases

Our previous engineering efforts increased the POA content from 8.3% to 12.0% and the total C16 content in the culture supernatant from 17.8% to 38.6% in strain *Pp*#39. However, these levels were still comparably low, particularly considering that other yeast species exhibit POA contents of 30–70% (Table 2). Aside from the thioesterase and desaturase activities, the fatty acid synthase (FAS) itself has a considerable impact on the fatty acid composition. In particular, the ketoacyl synthase, malonyl/palmitoyl-transferase (or malonyl acetyl transferase) domains, and attached thioesterase domains in mammalian FAS govern the final acyl-chain length [93]; thus, we anticipated that the endogenous *P. pastoris* FAS contributed to the high C18 content in our strains.

**Figure 6:**
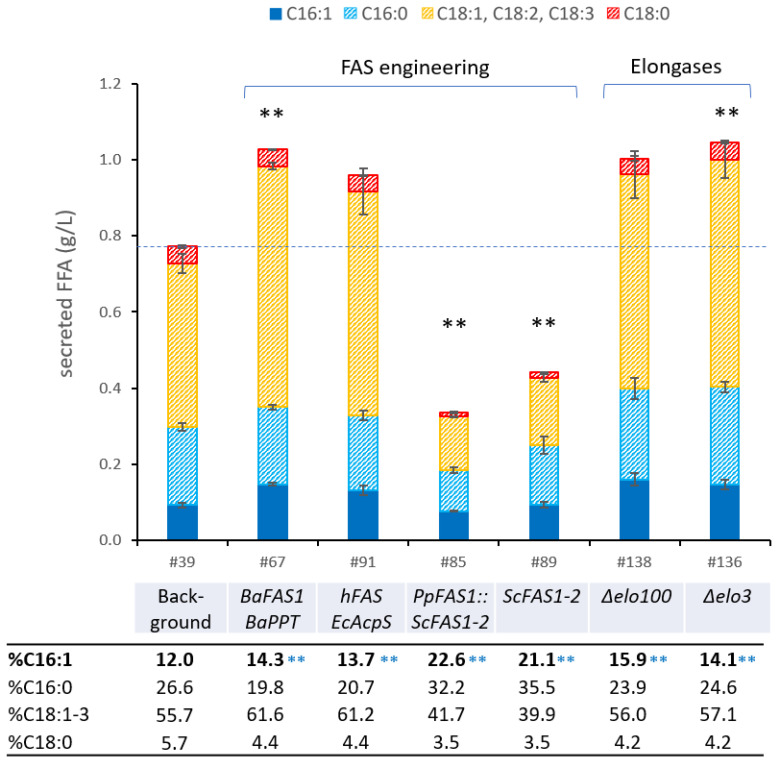
Fatty acid analysis of *P. pastoris* strains: modification of fatty acid profile by expressing heterologous fatty acid synthases and deleting FA elongases. FFAs were extracted from cell culture supernatant after cultivation in DWPs with BMD11 medium for 96 h at 320 rpm and 28 °C. The numbers given (#) refer to the *P. pastoris* strains listed in Table 1 with respective modifications compared to the background strain *Pp*#39 listed below. The table presents the percentage of individual FA species produced by the different engineered strains. All data are presented as mean ± SD of biological triplicates. Statistical analysis was performed using two-sample two-tailed Student’s *t* test (comparison to respective background strain; ** *p* value < 0.01; black asterisks indicate comparison of total secreted FAs; blue asterisks indicate comparison of C16:1 content). Minor fatty acid species (C17:1, C17:0, C14:0, and very long chain fatty acids, sum amounting to ≤3% of total fatty acids) were not included in this analysis.

It has previously been shown that the co-expression of an Actinomyces type I FAS from *B. ammoniagenes* with a wax ester synthase in *S. cerevisiae* increased fatty acid ethyl ester production by 6.3-fold [94]. In the same study, bacterial type I FAS expression could rescue growth defects in a *S. cerevisiae fas1* mutant and, astonishingly, the resulting strain displayed 2.7-fold higher palmitoleic acid titers as compared to the *S. cerevisiae* FAS wild-type. In a separate study, the introduction of human FAS was also shown to remedy the growth defect of a *S. cerevisiae fas2* mutant—knockout of either *FAS1* or *FAS2* abolishes fatty acid synthesis [95]. Mammalian FAS offer the advantage of a relatively open structure that allows efficient access of thioesterases to the growing acyl-chain. Additionally, the human FAS possesses an attached thioesterase domain with high specificity for C16 fatty acyl ACPs [95]. Since strain *Pp*#39 predominantly produced C18 fatty acids, we attempted to replace the endogenous FAS complex with the *FAS* genes from *B. ammoniagenes* (*BaFAS*) or *H. sapiens* (*hFAS*), respectively. The required phosphopantetheine transferase proteins *BaPPT* and *EcAcpS* were co-expressed. However, attempting a complete replacement of *P. pastoris FAS1* in our background resulted in lethality. This suggests that the metabolic burden induced by producing FFAs, combined with a substantial alteration in the FA profile, impeded cell growth. Interestingly, co-expression of individual *BaFAS* or *hFAS* and respective *PPT* (strains *Pp*#67 and *Pp*#91) with the intact endogenous *PpFAS* system increased the FFA titers by 33% and 24% (Figure 6), respectively, although the POA contents in the secreted fraction were only elevated by 2.3% and 1.7%, respectively. These findings align with results from Eriksen et al. [94], where alterations in the FA profile by heterologous *FAS* expression were not as evident when the endogenous *FAS* was active. The results furthermore suggest that the endogenous FAS or fatty acid elongases efficiently elongate C16 fatty acids provided by the heterologous FAS variants, which was indicated by the appearance of vaccenic acid mass fragments in GC-MS.

Fungal FAS belong to the cytosolic type I FAS complex and are typically composed of two polypeptides, Fas1p and Fas2p. The structural organization of the *P. pastoris* FAS complex has been elucidated recently, and several distinctions to the *S. cerevisiae* FAS in both the structural and enzymatic domains were identified [96].

Consequently, we explored whether the expression of the heterologous fungal FAS from *S. cerevisiae*, which primarily produces C16 and C18 acyl-CoAs [87], could replace endogenous FAS activity and how this approach influences the FA profile of *P. pastoris,* as previous reports have indicated that *S. cerevisiae* exhibits higher C16 and POA contents than *P. pastoris* (see Table 2). The seamless replacement of *PpFAS1* with an *ScFAS1–ScFAS2* expression cassette driven by the bidirectional promoter P*_HTX1_* in strain *Pp*#39 proved to be successful, generating strain *Pp*#85, which showed elevated POA and total C16 contents of 23% and 55%, respectively (Figure 6). The subsequent replacement of *PpFAS2* by a *HIS4* expression cassette in strain *Pp*#85 did not lead to further changes in the FA titer or POA content (strain *Pp*#115). Notably, strain *Pp*#85 exhibited reduced growth and compromised productivity, releasing only 0.35 g/L of FFAs into the supernatant, representing a 56% reduction compared to the parental strain. Chromosomal integration of the *ScFAS1–ScFAS2* expression cassette in the *TEFup* locus with the native *PpFAS* intact (*Pp*#39 background) yielded strain *Pp*#89, which exhibited an identical fatty acid composition as strain *Pp*#85 but showed modestly increased FFA titers of 0.44 g/L, which is still 43% lower compared to the parental strain *Pp*#39. Importantly, *Pp*#89 displayed compromised growth, signifying cellular stress induced by alterations in the FA profile, particularly due to the expression of *ScFAS*.

### 3.8. Engineering the Endogenous Fatty Acid Elongase System

Following the substitution of the native *P. pastoris* FAS, the POA content still remained below 25%, and we hypothesized that the endogenous elongase system was likely responsible for extending the POA to form vaccenic acid (C18:1-Z11), resulting in diminished POA content and elevated C18 titers. Differentiating between vaccenic acid and oleic acid proved to be challenging due to their high structural similarities, and the presence of vaccenic acid could only be tentatively confirmed through mixed mass spectra. To unambiguously validate our hypothesis, we undertook the deletion of the FA elongases responsible for the extension of POA.

In *S. cerevisiae*, FA elongation is mediated by elongases Elo1p, Elo2p, and Elo3p [55]. While Elo1p is responsible for elongation of medium-to-long-chain fatty acids like C16:1 [97,98], the other elongases (Elo2p and Elo3p) are involved in extending fatty acids from C16 and C18 up to C22 and C26 for sphingolipid biosynthesis [99]. Consequently, all three isoforms presented viable deletion targets. A homology search in *P. pastoris* CBS7435 strain revealed three putative elongases: Elo2p (CAH2449582.1; 66% identity to *Sc*Elo2p, 50% identity *Sc*Elo1p, 48% identity to *Sc*Elo3p), Elo3p (CAH2449986.1; 58% identity to *Sc*Elo3p, 53% identity to *Sc*Elo2p, 47% identity to *Sc*Elo1p), and Elo100p (CAH2449019.1; 13–14% identity and 20–23% similarity to all *S. cerevisiae* elongase isoforms), which were previously annotated by Sturmberger et al. [46]. Since we could not unambiguously allocate the different isoforms based on homology, we decided to delete all three putative elongase genes in parallel in strain *Pp*#39. The deletions of *ELO3* and *ELO100* resulted in a modest increase in the POA content but, interestingly, the resulting strains displayed increased FA secretion (improvement of 35% and 30%, respectively; Figure 6: strains *Pp*#138 and *Pp*#136). Unfortunately, our attempts to delete *ELO2*, the isoform with the highest homology to *Sc*Elo2p, were unsuccessful, and subsequent efforts to obtain the deletion through supplementation with very long-chain fatty acids (VLCFA) to compensate for a possible lack in VLCFA also proved fruitless. It is important to note that the engineered strains exhibited low acyl-CoA synthase activity due to the deletions of the *FAA1* and *FAA2* genes, which hinders the incorporation of FAs into the metabolism. However, some degree of VLFA activation to CoA thioesters should be facilitated by Fat1p, the very long-chain fatty acid transport protein [59,100], which was still present in strain *Pp*#39.

### 3.9. Evaluation of FA Production of Strain Pp#85 in Bioreactor Cultivations

**Figure 7:**
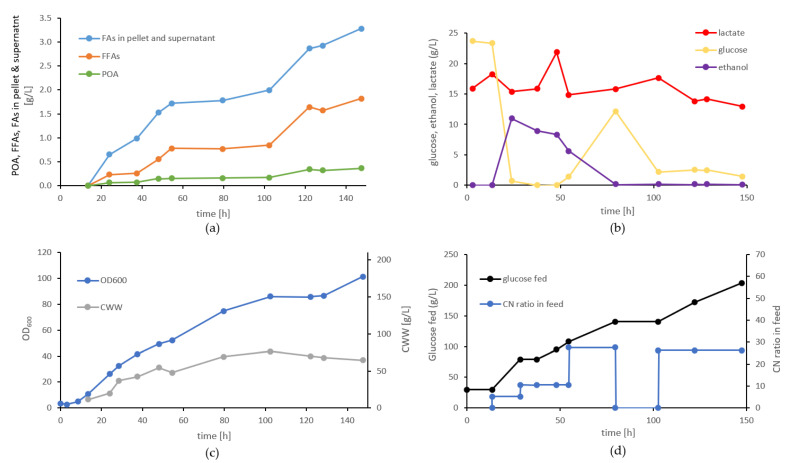
Nitrogen-limited fed-batch bioreactor cultivation of strain *Pp*#85. (**a**) Product titers of secreted FFAs, secreted POA, and total FAs (intracellular and secreted FAs); (**b**) amounts of determined fermentation by-products (lactate and ethanol) and residual glucose concentrations; (**c**) optical cell density (OD_600_ values) and cell wet weight (CWW); (**d**) glucose concentration and CN ratio of feeding solution.

After implementing various strategies to enhance the FA secretion and increase the POA content, we conducted an in-depth analysis of the strain *Pp*#85 exhibiting the highest relative POA content in a glucose-fed batch fermentation. Throughout the fermentation process, we adopted different CN ratios increasing from CN5 to CN28 (Figure 7d). This strategy was designed to promote cell growth in the earlier stages of fermentation and enhance FFA production towards the later stages. After 148 h of cultivation, we obtained 0.37 g/L of free POA (~19% of total fatty acids) in the supernatant (0.61 g/L in total—pellet and supernatant), while 210 g/L of glucose was consumed. The total FFA titer reached 1.8 g/L (Figure 7a,b), and the cell wet weight reached 70 g/L, representing an approximately 5-fold increase in productivity (4.9-fold for POA) and biomass compared to the DWP cultivations. Specific productivity was not improved in the process. As the fermentation progressed, we observed a reduction in the total C16 content from 50% at 24 h to 40% at 148 h, while the C18:1 content increased over time. This observation aligns with previous studies that demonstrated that extended cultivations of engineered *S. cerevisiae* strains promote C18:1 synthesis, potentially due to the upregulation of FA elongases [61]. HPLC measurements of the fermentation supernatant revealed the excretion of lactate and ethanol during the initial growth phase (Figure 7c). These results suggest a potential imbalance in NAD+ cofactor recycling, especially during the initial growth phase. The excessive NADH generated in glycolysis is consumed by lactate synthesis to maintain physiological NAD+ concentrations. This imbalance could compromise the fatty acid yield, as lactate synthesis consumes pyruvate, an essential precursor for cytosolic acetyl-CoA synthesis.

### 3.10. Evaluation of Metabolic Engineering Strategies for POA Production and Secretion

**Figure 8:**
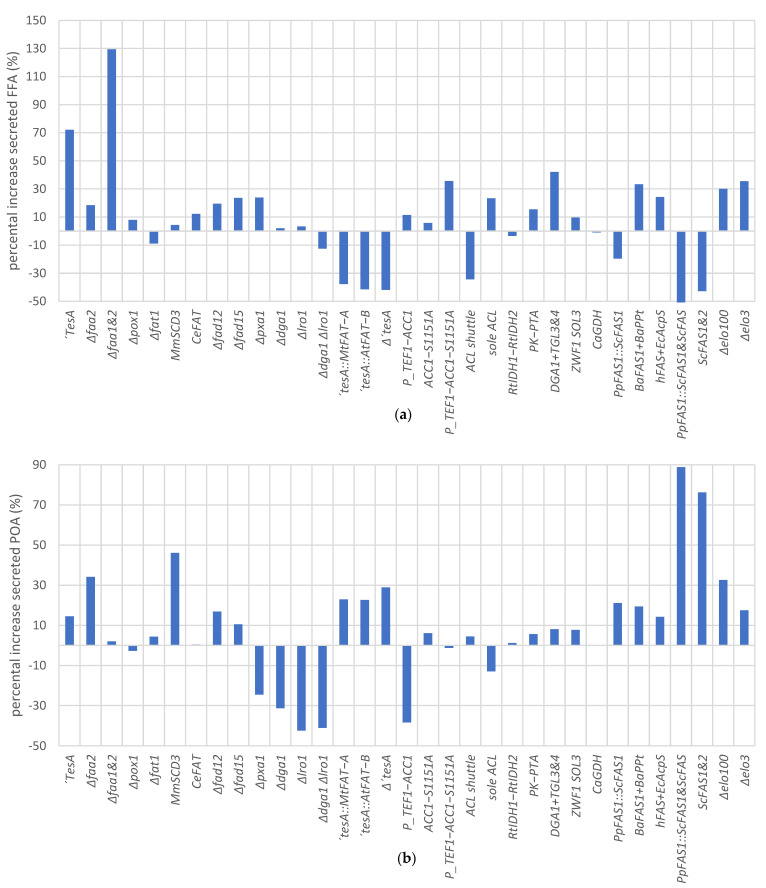
Engineering targets and their impact on total FA production and POA production in *P. pastoris*. (**a**) Percental increase in secreted FAs over the background strain enabled by listed modifications. Note: the improvement facilitated by ‘*TesA* is calculated from strain *Pp*#39 versus *Pp*#53, and the increased productivity for strain *Pp*#32 is calculated from total FA content (intracellular + supernatant; comparison of strain *Pp*#32 versus wt; see Supplementary Figure S4). (**b**) Percental increase of POA secretion over the background strain enabled by listed modifications.

Metabolic engineering studies have been focusing on either FA secretion, producing a wider FA spectrum [62,101,102], or the intrinsic production of POA [103,104], yet there are no studies specifically aiming at increasing POA secretion in recombinant microbes. To fill this knowledge gap, we have undertaken a comprehensive metabolic engineering approach to harness the potential of *P. pastoris* as a platform for the secretory production of POA. The summarized results (Figure 8) provide a valuable overview of the high-impact targets that promote FA secretion and profile alterations in this yeast.

Relevant improvements in FA secretion were facilitated through increased expression of ∆9-desaturases, enhancing precursor-supplying reactions, and manipulating the “lipid body bypass” pathway, among others. Plenty of these findings align with commonly employed engineering targets in the literature [61,102]. Regarding the engineering of *P. pastoris*’ FA profile, the expression of ∆-9 desaturases, human or bacterial type I FA synthases, and the deletion of ∆12- and ∆15-desaturases, as well as FA elongases, contributed to the improved POA content.

Among the various strategies employed, three approaches stood out in enhancing the overall FA secretion or elevating the POA content: the deletions of fatty acyl-CoA synthetases, the overexpression of the leaderless *E. coli* thioesterase, and the expression of *S. cerevisiae* FA synthase. The former strategies increased the FA synthesis by 130% and 70%, respectively, while the latter elevated the POA content from 12% to 22% of the total FA. There is a consensus in the literature that deletions of fatty acyl-CoA synthetases and expression of thioesterases are among the most effective strategies to increase FA synthesis [61,62,102].

Our observations, however, indicate that especially these high-impact modifications, which led to increased FA secretion and profound alterations in the intrinsic FA profile, coincided with reductions in the growth rate and final cell wet weight (CWW). This is underscored by the fed-batch bioreactor cultivation of strain *Pp*#85, heavily modified in the FA profile, which yielded only 1.8 g/L FFA and a final biomass of 70 g/L CWW. This is not surprising, as the FA composition has considerable impact on membrane fluidity, homeostasis, and various other cellular processes. We recognize that beyond a certain threshold, extensive modifications to the FA profile can rapidly lead to diminished cellular fitness.

To achieve significant changes in the product profile while maintaining cell viability, the focus should therefore shift to enhancing the specificity of the terminal reactions, i.e., thioesterases or lipases [59,80], which release FAs from acyl-CoA pools or neutral lipids. In this context, exploring a wide array of different thioesterases and lipases may be beneficial, allowing for identification of ideal gene candidates that can selectively release specific FAs. In this way, the intrinsic FA composition may largely remain intact. Thus, the secretion of FAs into the growth medium not only simplifies downstream processing but also holds immense potential for tailoring FA profiles.

**Table 2:** FA composition of wild-type and engineered yeasts producing elevated levels of palmitoleic acid according to the literature. Total FA titers are usually given as g/L FFAs. Notably, in most studies, internally bound FAs (e.g., in triglycerides or phospholipids) are included in the free FA titer according to the extraction method applied; however, downstream processing of internally stored FAs requires additional unit operations. We also list examples where POA is exclusively produced intracellularly. SF, shake flask; FB, fed-batch reactor cultivation; N-lim., nitrogen-limited cultivation; MM, minimal medium; POA, palmitoleic acid; PA, palmitic acid; αLEA, alpha linolenic acid; LA, linoleic acid; OA, oleic acid (or vaccenic acid for *P. pastoris* strains); SA, stearic acid; other FA, other FAs (C17:1 and C17:0 for our strains); *, alpha linolenic acid content included in linoleic acid content (due to lack of separation); **, g POA/g dry weight; (s), FFAs in supernatant; (t), total FAs (this includes FFAs in supernatant and covalently linked FAs in the pellet fraction; (i), intracellular production.

Strains (Wild-Type)	Conditions	Fatty Acids (%)							POA (g/L)	FA (g/L)	Ref.
		C16:1 POA	C16:0 PA	C18:3 αLEA	C18:2 LA	C18:1 OA	C18:0 SA	other FA			
*P. pastoris* CBS7435	YPD, SF, (72 h)	5.5	5.1	10.9	29.5	45.7	0.8	2.5	0.05	1 (i)	this study
*Kluyveromyces polysporus* DBM 2171	N-lim. MM, SF (n/d~48–96 h est.)	74.5	7.2	0	0.9	17.1	0.3	0	0.16 g/g DW **	n/d	[74]
*S. cerevisiae* DBM 2115	N-lim. MM, SF (n/d~48–96 h est.)	59.8	11.5	0	2	24	2.7	0	0.08 g/g DW **	n/d	[74]
**Strains (engineered)**	**Conditions**	**C16:1 POA**	**C16:0 PA**	**C18:3 αLEA**	**C18:2 LA**	**C18:1 OA**	**C18:0 SA**	**other** **FA**	**POA (g/L)**	**FA (g/L)**	**Ref.**
*P. pastoris Pp*#85	N-lim. MM, FB (150 h)	18.7	27.9	*	6.3	40.7	3.4	3.0	0.37(s) 0.61 (t)	1.8 (s) 3.3 (t)	this study
*P. pastoris* *PC124H*	N-lim. MM, FB (220 h)	9	42	n/d	27	17	4	n/d	2.1	23.4 (t)	[62]
*S. cerevisiae MK*	N-lim. MM, FB (144 h)	57.5	9.4	0	0	23	2	8.1	6.56	11.4 (i)	[103]
*S. cerevisiae* Y & Z055E (CEN.PK113-5D)	N-lim. MM, FB (~250 h)	36.5	32.9	0.0	0.0	18.7	6.3	5.4	9.1	25 (t)	[101]
*Scheffersomyces segobiensis* SS-12	MM, FB (~185 h)	24.9	9.6	0.0	4.0	58.1	0.0	3.4	7.3	29.6 (i)	[104]
*E. coli* SBF50	N-lim. MM, FB (~55 h)	30.3	14.0	0.0	0.0	16.1	0.0	39.5	10	33.6 (s)	[18]

Even though the total FFA secretion and relative POA content in our engineered strain could not match the highest reported values in the literature, amounting to 10–30 g/L FFA with POA contents of 20–60% (Table 2), we were still able to raise the POA content from 5.5% in the wild-type strain to above 20%, which is a 4-fold improvement. Moreover, we established FA secretion in *P. pastoris*, commonly generating FFA titers of 1 g/L in shake flask cultivation media. While it appears that host systems like *S. cerevisiae*, *Kluyveromyces polysporus*, and *Scheffersomyces segobiensis* should be favored for POA production due to their inherently high POA content or lipid accumulating phenotype, *P. pastoris* possesses unique advantages over these host systems. These advantages include *P. pastoris*’ ability to attain high cell densities in fermentations, the efficient expression of recombinant proteins, and the absence of the Crabtree effect [39,43]. These characteristics enable the generation of considerable numbers of whole-cell biocatalysts and facilitate rapid flux through heterologous pathways, while minimizing carbon loss and supporting the efficient expression of terminal enzymes for various biotechnological applications.

## 4. Conclusions

While our study primarily focused on enhancing POA secretion, the principles and targets identified herein can be readily adapted for the production of other long-chain FAs, i.e., oleic acid or PUFAs or tailored FA profiles, such as those found in palm oil, palm kernel oil, or cocoa butter. This research opens new avenues for the microbial production of valuable FAs with the potential to enable the synthesis of novel FA-derived products while reducing the environmental footprint associated with traditional production methods. As the demand for sustainable alternatives continues to grow, the development of efficient FA secretion systems represents a significant step forward in the field of metabolic engineering.

## Figures and Tables

**Table 1:** List of *P. pastoris* strains used/generated in this study. All strains are based on the *P. pastoris* CBS7435 wild-type strain. All numbered *P. pastoris* strains were generated in this study (except for the wild-type strain). Gene abbreviations: *DAS1*, dihydroxyacetone synthase 1; *FDH1*, formate dehydrogenase 1; *FBP1*, fructose-bisphosphatase; *ARG4*, argininosuccinate lyase; *AOX1*, alcohol oxidase 1. Abbreviations for integration sites: Chr1–Chr4, chromosomes 1 to 4; NS1-10 neutral site (integration site) on respective chromosome (i.e., Chr1_NS10: neutral site 10 on chromosome 1); *TEF*up, integration site upstream of transcription elongation factor 1 locus; *FLD*up, integration site upstream of S-(hydroxymethyl)glutathione dehydrogenase.

Strain (*Pp*#)	Genetic Background	Further Modifications
**Wild-Type (WT)**	**CBS7435**	
12	WT	Δ*fad12*
14	WT	Δ*ole1-1*
16	WT	Δ*ole1-2*
28	*Pp*#16	*his4::*P*_GAP_-*‘*TesA-*TT*_ARG4_*
29	*Pp*#28	Δ*faa2*
32	*Pp*#29	Δ*faa1*
35	*Pp*#32	Δ*pox1*
37	*Pp*#35	Δ*fat1*
39	*Pp*#35	*FLDup::*P*_TEF1_-MmSCD3-*TT*_ARG4_*
44	*Pp*#35	Δ*dga1*
45	*Pp*#35	Δ*lro1*
46	*Pp*#35	Δ*dga1 Δlro1*
47	*Pp*#39	*his4::*P*_GAP_-MtFAT-A-*TT*_ARG4_*
48	*Pp*#39	*his4::*P*_GAP_-AtFAT-B-*TT*_ARG4_*
49	*Pp*#39	*acc1::*P*_TEF1_-ACC1-*TT*_ACC1_*
50	*Pp*#39	*acc1::Acc1^S1151A^-*TT*_ACC1_*
51*	*Pp*#35	Δ*dga1* Δ*lro1 Δare2*
51	*Pp*#39	*TEFup::*P*_PGK1_-CeFAT-*TT*_ARG4_*
53	*Pp*#39	*his4::*P*_GAP_-Δ*‘*tesA-*TT*_ARG4_*
54	*Pp*#39	*acc1::*P*_TEF1_-ACC1^S1151A^-*TT*_ACC1_*
67	*Pp*#39	*TEFup::*P*_HTX1_-BaFAS1-*TT*_ARG4_-BaPPT-*TT*_AOX1_*
85	*Pp*#39	*PpFAS1::*P*_HTX1_-ScFAS2-*TT*_PpFAS1_-ScFAS1-*TT*_PpFAS1_*
89	*Pp*#39	*TEFup::*P*_HTX1_-ScFAS2-*TT*_ARG4_-ScFAS1-*TT*_AOX1_*
91	*Pp*#39	*TEFup::*P*_HTX1_-hFAS-*TT*_ARG4_-EcACPS-*TT*_AOX1_*
112	*Pp*#50	*Chr1_NS6::P_HHX1_-ZWF1-*TT*_ARG4_-SOL3 -*TT*_TEF1_*
113	*Pp*#50	*faa2::*P*_GAP_-RAD52-*TT*_AOX1_ Chr1_NS10_P_GAP_-CaGDH-*TT*_AOX1_*
114	*Pp*#50	*faa2::*P*_GAP_-RAD52-*TT*_AOX1_*
115	*Pp#85*	*PpFAS2::*P*_HIS4_-HIS4-*TT*_PpFAS2_*
121	*Pp*#50	*faa2::*P*_GAP_-RAD52 Chr2_NS3::*P*_TPI_-RtACL-*TT*_DAS1_-*P*_GAP_-MmME-*TT*_HTB_-*P*_RP_-PpCTP1-*TT*_PGK1_-PpMDH3-*TT*_TEF1_*
127	*Pp*#35	*FLDup::*P*_TEF1_-MmSCD3-T2A1-PpCypb5-T2A2-PpCyb5R-*TT*_ARG4_*
129	*Pp*#35	*FLDup::*P*_TEF1_-MmSCD3-T2A1-MmCypb5-T2A2-MmCyb5R-*TT*_ARG4_*
135	*Pp*#39	Δ*pxa1*
136	*Pp*#39	Δ*elo3*
138	*Pp*#39	Δ*elo100*
140	*Pp*#39	Δ*fad15*
141	*Pp*#50	*faa2::*P*_GAP_-RAD52 Chr4_NS7::*P*_CAT_-DGA1-*TT*_TEF1_-*P*_HHX2_-TGL3-*TT*_HTA_-TGL4-*TT*_GAP_*
142	*Pp*#50	*faa2::*P*_GAP_-RAD52-*TT*_AOX1_ Chr2_NS3::*P*_ENO1_-RtACL-*TT*_DAS1_*
145	*Pp*#50	*faa2::*P*_GAP_-RAD52 Chr3_NS7::*P*_HHX2_-BbPK--*TT*_FDH1_-CkPTA-*TT*_FBP1_*
148	*Pp*#85	*Chr4_NS7::*P*_GAP_-ScOLE1-*TT*_DAS1_*
150	*Pp*#39	Δ*fad12*
153	*Pp*#50	*faa2::*P*_GAP_-RAD52-*TT*_AOX1_ Chr2_NS3::*P*_ENO1_-RtACL* P*_PpIDH1_-RtIDH1-*TT*_PpIDH1_*P*_PpIDH2_-RtIDH2-*TT*_PpIDH2_*

## Data Availability

All relevant data are within the paper and its Supplementary Material files.

## Supplementary Materials

The following supporting information can be downloaded at: https://www.mdpi.com/article/10.3390/bioengineering10121412/s1, Figure S1: Cas9 gRNA vector map (pPpT4_pHTX1_hsCa); Figure S2: Chromatograms for fatty acid methyl-ester analysis of the *P. pastoris* CBS7435 wild type strain (a), and strain *Pp*#12 with the deletion of *FAD12* (b); Figure S3: Relative FFA titers in the supernatant of strain *Pp*#39 cultivated in different media with four nitrogen sources (N-source) and varying carbon to nitrogen rations; Figure S4: Fatty acid analysis of all *P. pastoris* strains generated in this study and wild type strain; Figure S5: Thin layer chromatography of lipid extracts from *P. pastoris* strains engineered in neutral lipid storage; Table S1:Synthesized codon optimized genes and DNA fragments used in this study; Table S2: Primer list for construction of knock-out cassettes, expression cassettes and CRISPR plasmids; Table S3: CRISPR target sites used in this study; Table S4: Cell wet weights from strains cultivated in 96-deep well plates; Table S5: Medium compositions tested for free fatty acid production with strain *Pp*#39; Supplementary method S1: Extended GC method for separation of C18:1, C18:2 & C18:3; Supplementary method S2: Lipid extraction from *P. pastoris* strain and lipid analysis with thin layer chromatography; Additional information S1: expression cassette assembly. References [105,106,107] are cited in the supplementary materials.
