# Supplementary material for: Tuning Fatty Acid Profile and Yield in Pichia pastoris
Source: Bioengineering (Basel). 2023 Dec 12;10(12):1412. doi: 10.3390/bioengineering10121412 (PMC10741089; doi:10.3390/bioengineering10121412)
Supplement: Supplementary file 1 [file bioengineering-10-01412-s001.zip › bioengineering-2725637-supplementary.pdf]

Supplementary files

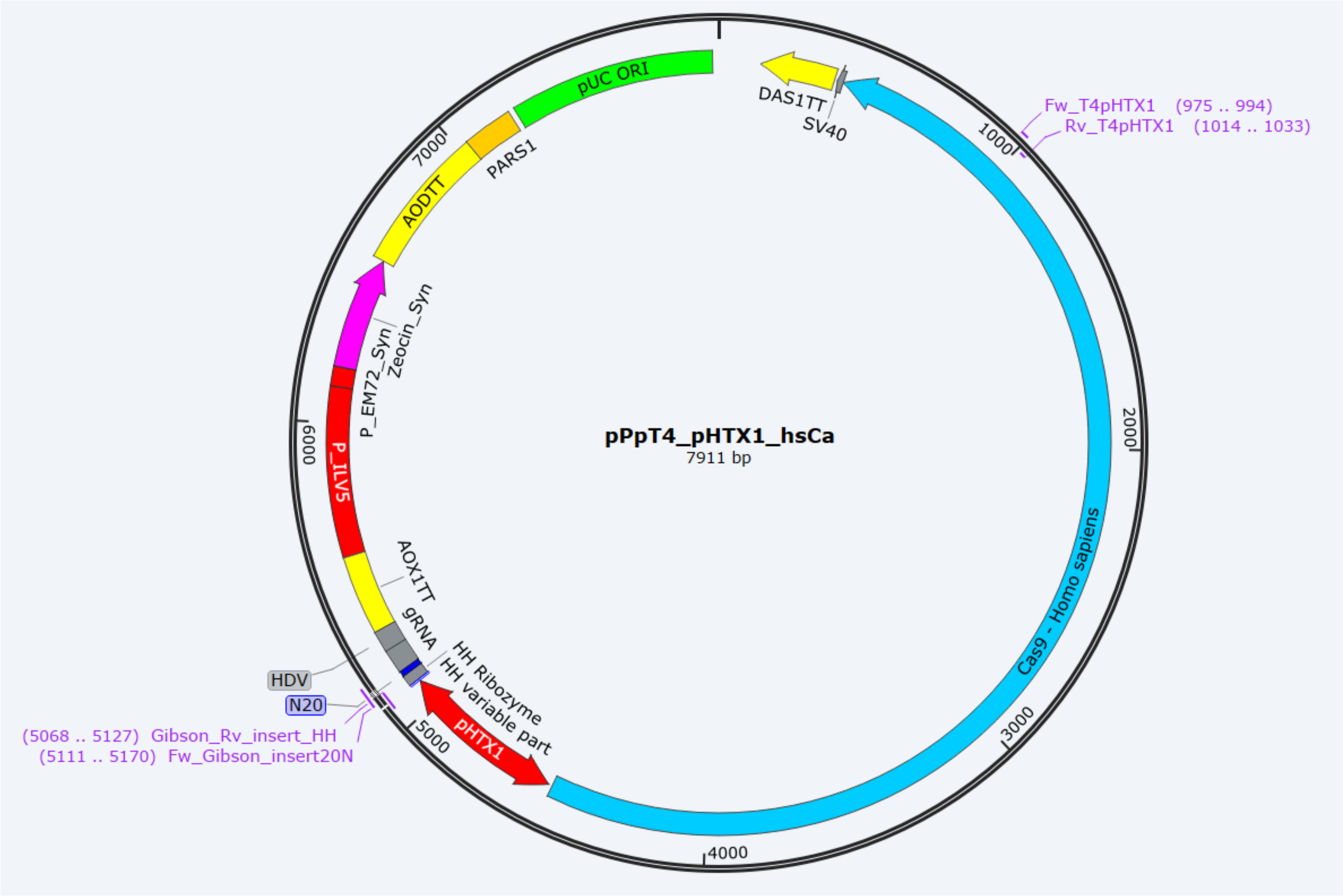

Figure S1. Cas9 gRNA vector map (pPpT4_pHTX1_hsCa): Primers for insertion of variable regions (N20 sequence and hammer head variable region) are indicated on the bottom left. Respective partner primers are indicated on the top right. Cas9 gRNA plasmids targeting new loci are assembled from two PCR products amplified with Fw_Gibson_insert20N & Rv_T4pHTX1 and Gibson_Rv_insert_HH & Fw_T4pHTX1. Cas9 and gRNA expression are driven by the bidirection P*HTX1* promoter. Vector maintainence is conferred by a Zeocin resistance cassette and a PARS1 sequence. Further details on vector function are given in the work of Weninger *et al.*.

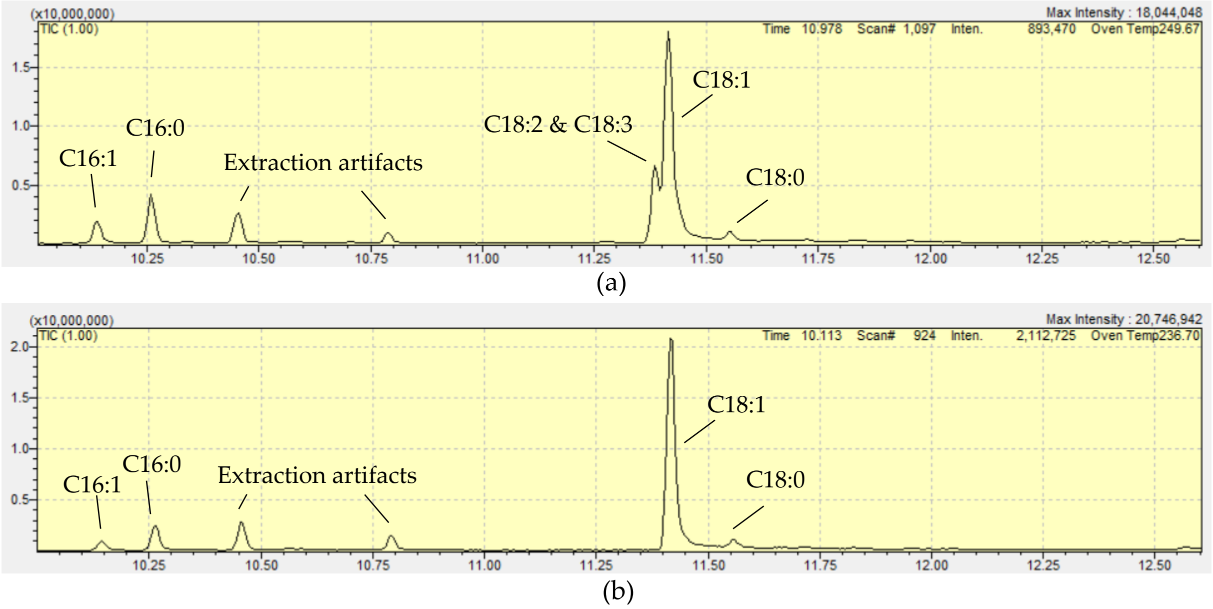

Figure S2. chromatograms for fatty acid methyl-ester analysis of the *P. pastoris* CBS7435 wild type strain (a), and strain *Pp*#12 with the deletion of *FAD12* (b). *Pp*#12 lacks polyunsaturated fatty acids (C18:2 and C18:3 common peak at ~11.37 min). Note: C18:2 and C18:3 elute simultaneously with this method. Clear separation was achieved with a different analysis method (Supplementary method S1).

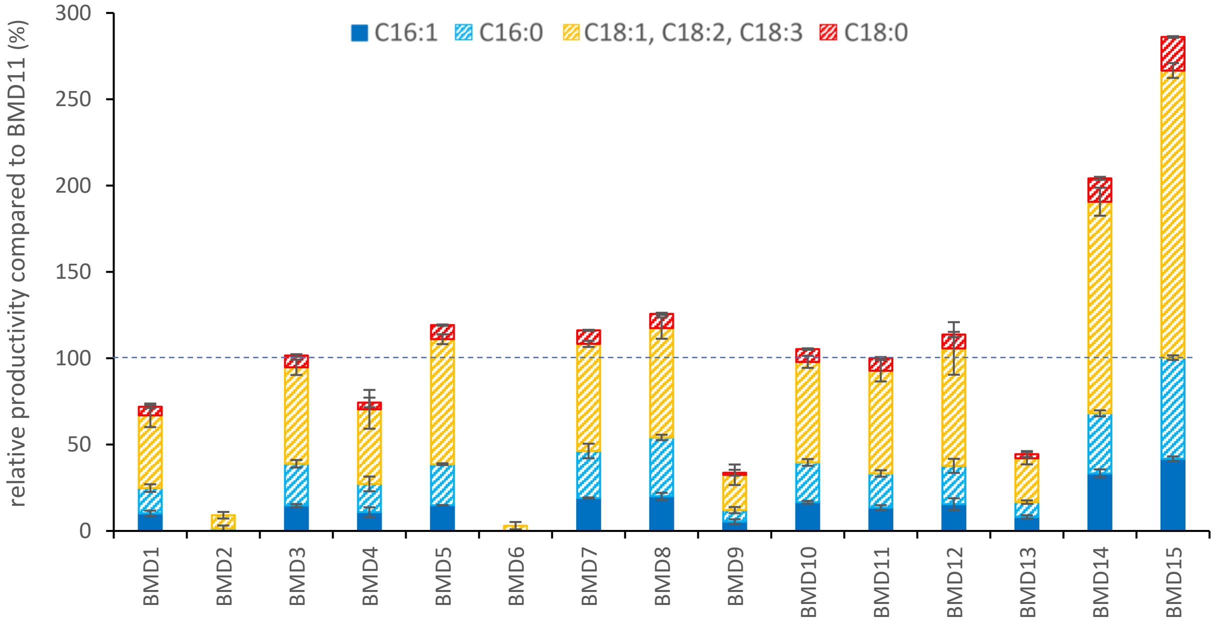

%C16:1	14.0	n/d	14.5	14.6	12.5	n/d	16.4	16.0	16.1	15.8	13.5	13.6	18.2	16.3	14.6
%C16:0	20.5	n/d	23.8	22.0	19.9	n/d	23.4	27.1	19.7	21.9	19.8	19.5	19.3	17.0	20.5
%C18:1-3	58.6	n/d	55.0	58.1	60.7	n/d	53.4	50.4	60.7	55.3	59.5	59.8	57.0	60.0	58.1
%C18:0	6.9	n/d	6.8	5.3	6.9	n/d	6.7	6.6	3.5	7.0	7.2	7.1	5.5	6.6	6.8
N-source	AS	AS	AS	AS	AS	YE	YE	YE	AC	AC	AC	AC	P	P	P
N-source-conc. [g/L]	10.0	0.5	1.0	1.9	3.5	2.0	4.0	6.0	0.5	1.0	1.9	3.5	2.0	4.0	6.5
CN ratio	6	113	57	30	16	51	25	17	92	46	24	13	43	23	16

Figure S3. Relative FFA titers in the supernatant of strain *Pp*#39 cultivated in different media with four nitrogen sources (N-source) and varying carbon to nitrogen rations (compared to FFA titers obtained by cultivation in medium BMD11; media compositions are given in Supplementary Table 5). The table lists fatty acid contents, nitrogen sources (AS, ammonium sulfate; AC, ammonium chloride; YE, yeast extract; P, peptone), nitrogen source concentration and carbon to nitrogen ratios applied in the respective media. Cells were cultivated for 96 h at 28 °C and 320 rpm in 96-deep well plates (0.5 mL medium). The bars represent means ± SD of biological triplicates.

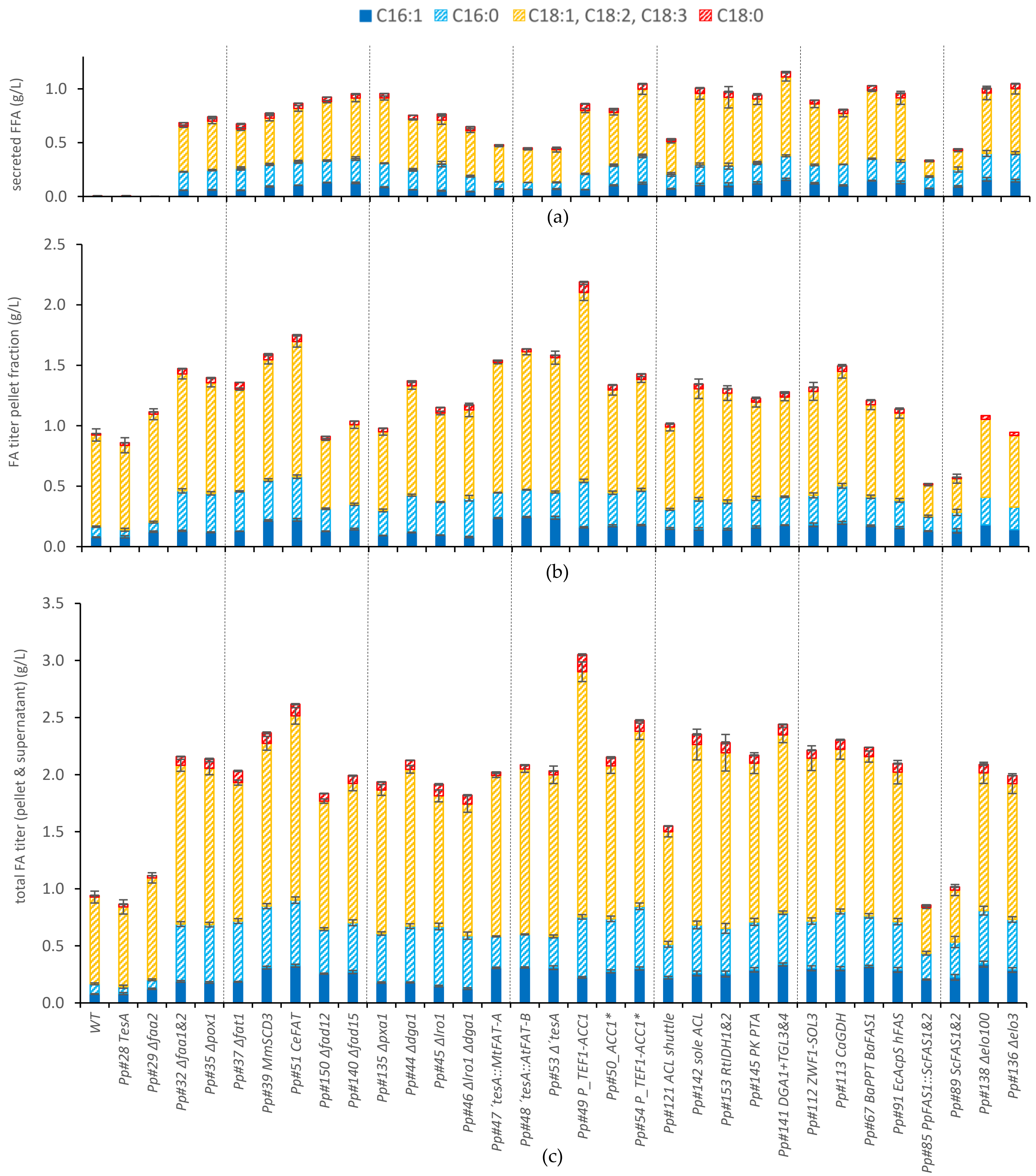

Figure S4: Fatty acid analysis of all *P. pastoris* strains (strain numbers and unique genetic features are given) generated in this study and wild type strain: (a) FFA in the supernatant, (b) fatty acids in the pellet fraction (sum of free fatty acids and bound fatty acids), (c) total fatty acids in the supernatant and the pellet

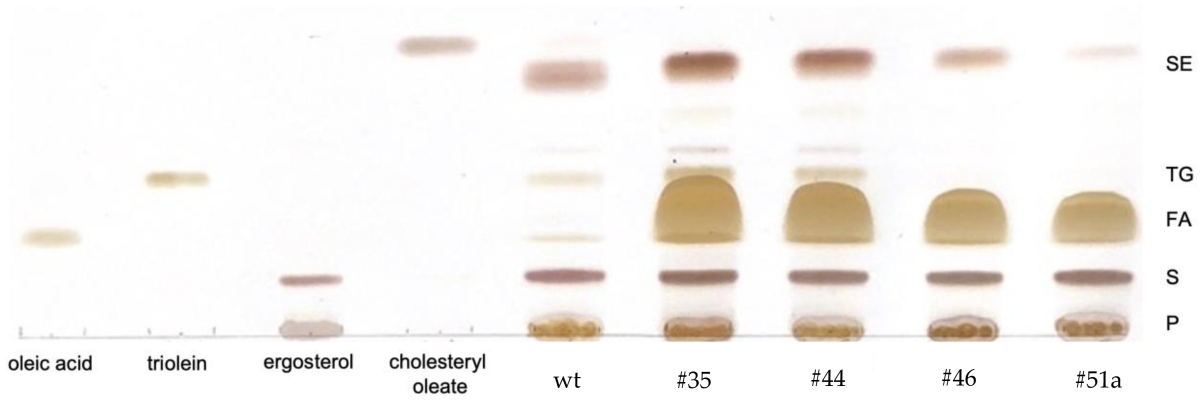

Figure S5. Thin layer chromatography of lipid extracts from *P. pastoris* strains engineered in neutral lipid storage *Pp*#44 (*Pp*#35 *Δdga1*), *Pp*#46 (*Pp*#35 *Δdga1 Δlro1*), *Pp*#51* (*Pp*#35 *Δdga1 Δlro1 Δare2)* and background strain *Pp*#35 (*Δole1-2 his4::pGAP-'TesA Δfaa2 Δpox1 Δfaa1*). Total intracellular lipids were extracted from 60 OD units of washed cells using chloroform and methanol. Lipid extracts were separated using a mobile phase composed of petroleum, diethyl ether and acetic acid (70:30:2; per vol.) on silica coated aluminum plates (detailed method description: Supplementary method 1). Oleic acid, triolein, ergosterol and cholesteryl oleate were applied on the left side as standards. Band labels (right side) – abbreviations: FA, free fatty acids; TG, triacylglycerides; S, sterols ; SE, sterol esters; P, phospholipids. Strains *Pp*#46 and *Pp*#51* with abolished acyl-acylglycerol transferase activity show no TG band. Strain *Pp*#51* displays reduced sterol ester synthesis, due to the deletion of *ARE2* (Acyl-CoA:sterol acyltransferase). Strains with deletions of acyl-CoA synthetases (all strains, except wild type) exhibit increased levels of FFA.

Table S1. Synthesized codon optimized genes and DNA fragments used in this study

Genes/T2A	Sequence 5′-3′
'TesA	ATGGACACCTTGTTGATTTTGGGTGACTCTTTGTCCGCCGGTTACAGAATGTCTGCTTCTGCTGCTTGGCCAGCTTTG<br>TTGAACGATAAGTGGCAATCCAAGACCTCCGTTGTTAACGCTTCTATCTCTGGTGACACTTCCCAGCAAGGTTTGGC<br>TAGATTGCCAGCTCTTTTGAAGCAACACCAGCCAAGATGGGTTCTTGTTGAGCTTGGTGGTAACGACGGTTTGAGA<br>GGTTTCCAACCACAACAGACTGAGCAGACCTTGAGACAAATCTTGCAGGACGTTAAGGCTGCTAACGCTGAGCCT<br>TTGTTGATGCAGATTAGACTGCCAGCCAACTACGGTAGAAGATACAACGAGGCTTTCTCCGCTATCTACCCAAAGT<br>TGGCTAAAGAGTTCGACGTTCCACTGCTGCCATTCTTCATGGAAGAGGTCTACTTGAAGCCACAGTGGATGCAAGA<br>TGACGGTATTCACCCAAACAGAGATGCCCAGCCATTCATTGCTGATTGGATGGCTAAGCAGTTGCAGCCATTGGTC<br>AACCACGACTCTTAA
AtFAT-B	ATGTTGCCAGACTGGTCTATGTTGTTGGCTGCTATCACCACCATTTTCTTGGCTGCTGAGAAGCAGTGGATGATGTT<br>GGATTGGAAGCCTAGAAGATCCGACATGCTGGTTGACCCATTCGGTATCGGTAGAATCGTTCAGGACGGTCTGGTT<br>TTCAGACAGAACTTCTCCATCAGATCCTACGAGATTGGTGCTGACAGATCCGCTTCCATCGAGACTGTTATGAACC<br>ACTTGCAAGAGACTGCCCTGAACCACGTTAAGACTGCTGGTTTGCTTGGTGACGGTTTCGGTTCTACTCCAGAGATG<br>TTCAAGAAGAACCTGATCTGGGTCGTCACCAGAATGCAGGTTGTTGTTGACAAGTACCCAACCTGGGGTGACGTTG<br>TTGAAGTTGACACTTGGGTTTCCCAGTCCGGTAAGAACGGTATGAGAAGAGACTGGTTGGTCAGAGACTGTAACAC<br>CGGTGAGACTTTGACTAGAGCTTCCTCCGTTTGGGTCATGATGAACAAGCTGACCAGAAGGCTGTCCAAGATTCCT<br>GAAGAGGTTAGAGGTGAGATCGAGCCATACTTCGTTAACTCTGACCCAGTTTTGGCTGAGGACTCCAGAAAGTTGA<br>CTAAGATCGACGATAAGACCGCCGACTACGTTAGATCCGGTTTGACTCCAAGATGGTCCGACTTGGATGTTAACCA<br>GCACGTGAACAACGTGAAGTACATCGGTTGGATTTTGGAGTCCGCTCCAGTCGGTATTATGGAAAGACAAAAGCT<br>GAAGTCCATGACCTTGGAGTACAGACGTGAATGTGGTAGAGACTCCGTCTTGCAATCCTTGACTGCTGTTACTGGTT<br>GCGACATCGGTAACTTGGCTACTGCTGGTGATGTTGAGTGTCAGCACTTGTTGAGATTGCAGGACGGTGCTGAAGT<br>TGTCAGAGGTAGAACTGAATGGTCCTCCAAGACTCCAACTACTACTTGGGGTACTGCTCCATAA
MtFAT-A	ATGGTGTCCGAGAGAACTAATGGTTCCGGTGGTGTTGTTTCTAACCCATCTTCTAACGGTTCCGCCTCCTTGTTGGA<br>AGAGTTGAGATTGGGTTCTTTGGCCGAGGACGGTTTGTCCTACAAAGAGAAGTTCATCGTCAGATCCTACGAGGTC<br>GGTATCAACAAGACTGCTACTATGGAAGCTATCGCCAACTTGTTGCAAGAGGCTGCTTGTAACCAGGTTCAGACTT<br>TGGGTTACTCCACTGACGGTATGGGTACTTCCTACACCATGAGACAGTTGCACTTGATCTGGGTTACTGCCAGAATG<br>CACATCGAGGTCTACAAGTATCCAGCTTGGGGTGACATGATCGAGATCGAGACTTGGTTTGAGGCTGGTAGAGTCG<br>GTACTAGAAGAGACTTCGTTGTTAAGGACGCCACCGGTACTGTTATCGGTAGAGCTACTTCTAAGTGGGTCGCTAT<br>GAACCAGGACACCAGAAGATTGCAGAAGGTCAACGACAAGATCCTGGAAGAGATTATGTCCCACGCTCCAAGAA<br>CTCCAAGATTGGCTTTCCCAGAAGAGGACAACTGCTCCTTGAAGAAGATCCCAAAGTTGGAGGAACCAGCTCAGT<br>ACTCCAAGTTGGGTTTGAAGGCTAGAAGGGCTGACTTGGACATGAACCAGCACGTTAACAACGTCACCTACATCG<br>GTTGGTTGTTGGAGTCTTTGCCACAAGAGATCATCGACACCCACGAGTTGAGAAAGGTCACTTTGGACTACAGACG<br>TGAGTGTCAACACGACGATACTGTTGACTCCCTGACCTCTATGGAATTGATGGACAACGCTGAGGCTATCCCAGAG<br>TTGAACGGTACTAATGGATCTGCCACTGAGAGAAAGCACGAAGAGGACTGTTGTAGATTCCTGCACTTCGTCAGAT<br>TCTCTGGTGACGGTCCAGAAGTCAACAGAGGTAGAACTGAATGGCGTAGAAAGTCCACCAGATAA
CeFAT	ATGACCCAGATCAAGGTTGACGCTATCATCTCCAAGCAGTTCTTGGCTGCTGACCTGAACGAGATCAGACAGATGC<br>AAGAAGAGTCCAAGAAACAGGTCATCAAGATGGAAATCGTCTGGAAGAACGTCGCCTTGTTTGTTGCCTTGCACA<br>TCGGTGCTTTGGTTGGTCTTTACCAGTTGGTTTTCCAGGCTAAGTGGGCTACTGTTGGTTGGGTTTTCTTGTTGCACAC<br>CTTGGGTTCTATGGGTGTTACTGGTGGTGCTCATAGATTGTGGGCTCACAGAGCTTACAAGGCTACCTTGTCTTGGA<br>GAGTGTTCCTGATGCTGATCAACTCCATTGCCTTCCAGAACGACATTATCGACTGGGCCAGAGATCATAGATGTCA<br>CCACAAGTGGACTGACACTGACGCTGATCCACATTCCACTAACAGAGGTATGTTCTTCGCTCACATGGGTTGGTTG<br>TTGGTCAAGAAGCACGACCAGTTGAAGATCCAAGGTGGTAAGTTGGACTTGTCCGACTTGTACGAAGATCCCGTCT<br>TGATGTTCCAGAGAAAGAACTACTTGCCCCTGGTCGGTATCTTCTGTTTCGCTTTGCCAACTTTCATCCCAGTCGTTT<br>TGTGGGGTGAGTCCGCTTTCATTGCTTTCTACACTGCTGCCCTGTTCAGATACTGTTTCACCTTGCATGCTACCTGGT<br>GCATCAACTCTGTTTCCCATTGGGTTGGATGGCAACCATACGATCATCAAGCCTCTTCCGTTGACAACCTGTGGACT<br>TCTATTGCTGCTGTTGGTGAAGGTGGTCACAACTACCATCACACTTTCCCTCAGGACTACCGTACTTCTGAACACGC<br>TGAGTTCTTGAACTGGACCAGAGTCTTGATTGACTTCGGTGCTTCCATCGGTATGGTCTACGACAGAAAGACTACCC<br>CAGAAGAGGTTATCCAGAGACAGTGCAAAAAGTTCGGTTGCGAGACTGAGCGTGAGAAGATGTTGCATAAGTTGG<br>GTTAA
MmSCD3	ATGCCAGGTCACTTGCTGCAAGAAGAAATGACTCCATCCTACACTACTACCACCACTATCACTGCTCCACCATCTG<br>GTTCCTTGCAAAACGGTAGAGAGAAGGTTAAGACCGTTCCACTGTACCTGGAAGAGGACATTAGACCAGAGATGA<br>AGGAGGACATCTACGACCCAACTTACCAAGACGAAGAAGGTCCACCACCAAAGTTGGAGTACGTTTGGAGAAAC<br>ATCATCCTGATGGCCTTGTTGCATGTCGGTGCCTTGTACGGTATTACTTTGGTTCCATCCTGCAAGCTGTACACCTGT<br>TTGTTCGCCTTCGTCTACTACGTCATCTCCATCGAAGGTATCGGTGCTGGTGTTCATAGATTGTGGTCCCACAGAACT<br>TACAAGGCCAGATTGCCTCTGAGAATCTTCCTGATTATCGCCAACACTATGGCCTTCCAGAACGACGTTTACGAGT<br>GGGCTAGAGATCATAGAGCCCACCACAAGTTCTCTGAAACTCATGCTGATCCACACAACTCCAGAAGAGGTTTCTT<br>CTTCTCTCACGTCGGTTGGCTGTTGGTTAGAAAGCACCCAGCCGTCAAAGAAAAAGGTGGTAAGCTGGACATGTCT<br>GACTTGAAGGCTGAGAAGTTGGTCATGTTCCAGAGAAGGTACTACAAGCCAGGTATCCTGCTGATGTGCTTCATCT<br>TGCCAACTCTTGTTCCTTGGTACTGCTGGGGTGAGACTTTCTTGAACTCCTTCTACGTTGCCACCTTGTTGAGATACG<br>CCGTTGTTTTGAACGCCACCTGGTTGGTTAATTCCGCTGCTCACTTGTACGGATACAGACCATACGACAAGAACATC<br>GACCCAAGACAGAACGCCTTGGTTTCCTTGGGTTCTATGGGTGAAGGTTTCCACAACTACCATCACGCTTTCCCTTA<br>CGACTACTCTGCTTCTGAGTACAGATGGCACATCAACTTCACCACTTTCTTCATCGACTGCATGGCTGCTTTGGGTCT<br>TGCTTACGACAGAAAGAGAGTTTCCAAGGCCACTGTCTTGGCCAGAATCAAGAGAACTGGTGACGGTTCTCACAA<br>GTCCGGTTAA

RtIDH1	ATGTTTCCAAAACCACTGGCTTTAAGAAACCAGCTTATAGGTCAGGCTAGGTCCGCCACAACTCTGGGTGTTGGAA<br>TCTCAAGACCCTCCGCTATTCAGCCTACCAAGTACGGAGGTGTTTATTCTGTAACTTTGATCCCAGGAGACGGAGT<br>GGGTAAAGAAATTACTCAATCCGTTGAGGAAATTTTTGAACACGCAAACGTCCCCGTAGAGTTTGAGAAGTTTAAT<br>GTCTCTGGTGGTACTTCCGAAGATGCTGCTTTGTTCAAAAGGTCAATGGATTCTCTGAGGAGAAACAAGGTAGGTT<br>TGAAGGGTATCTTATACACCCCCGTCGAGAGATCAGGACACACAAGTTGGAACGTTGCTATGAGACAGCAATTAG<br>ATATTTATGCCAGTGTGGTGCTGTGCAAGTCTGTCCCCGGTGTTCCTACTAGACACAAGGATGTAGATTTTGCTATC<br>ATTCGTGAAAATACTGAGGGTGAATACTCCGGTCTGGAACACCAATCTTCACCTGGTGTAGTGGAATCCCTTAAAA<br>TTATGACTCGTCACAAGACTGAGAGGATTGCCAGATTTGCTTTCGACTACGCTATCAAAAACGGTCGTAAACACGT<br>AACCGCTATTCACAAGGCAAACATCATGAAGTTGGGAGATGGTCTGTTTCTGAACACTTGCAGACGTGTAGCCGAG<br>GAGTATAAAGATTCCGGAATAACCTTTTCTGACATGATAGTCGATAACACCAGTATGCAACTTGTTAATAGACCCC<br>AACAATTCGATGTTATGGTTATGCCTAATCTGTATGGATCTATCATATCTAATATAGGAGCAGCTCTGGTGGGAGGT<br>CCTGGAATTGTGCCAGGAGCCGACATCGGTCGTGAGTTCGCATTGTTCGAACCCGGATGTAGACATGTCGCTAAAG<br>ATATTCAAGGTCAGGATTCCGCAAACCCAGCCGCAATGATTTTGAGTGCAACTATGCTTTTACGTTACCTTGGTTGT<br>GACCACCACGCAAATGCAATTGCTTCTAGTGTTTACAAAATTTTGGAAGAGGGAAAGATCCGTACCCCTGACTTGG<br>GAGGAACATCTCATACCACCGATTTCACCCATGCAGTTATTAAAGGATTGCAATAA
RtIDH2	ATGTTTACTAGGACTTCTTTGAGAACAGCATCTAGAGCATTTTCCACATCAGCTCCCAGGGCTTACGCCTCTCAGGG<br>TGCCGCTCCTAGTGGATATGTTCCCAATACTAATGCCGATGGTACTTATAATGTTACTTTAATTCCAGGTGACGGTA<br>TAGGACCAGAGATCTCCAGATCCGTAAAAGAGATTTATGACGCCGCAAAGGTTCCTATCAAATGGGAGGAGGTAA<br>GTGTAACACCAATCTTAGTAGACGGTGTTTCAACTATTCCAGCTGACGCTATCGCTTCTATCAAAAAGAACACTATT<br>GCCTTGAAAGGACCTTTGGCCACACCAATCGGAAAAGGTCATGTTTCCTTGAACTTAACCTTAAGGAGAACTTTCA<br>ATTTGTATGCAAACGTAAGACCATGCCGTTCAATTGAAGGTTACAAAACTGCTTACGATAATGTCGATACTGTTTTG<br>ATTAGGGAAAACACCGAAGGAGAGTACTCTGGTATCGAACATGAGGTGGTTGATGGTGTGGTACAATCAATTAAA<br>TTGATTACCCACAACGCTTCATCTAGAGTCGCAAGATACGCTTTCGAGTACGCTAGGGCAAACAAGAGACCTCAC<br>GTAACTGCTGTCCATAAAGCCCCTATCATGAAGATGTCCGATGGAATGTTTCTGAGAGCATGCCGTGAATTGGCTC<br>CAGAATATCCAGATATCAAATACGATGAGGATTTACTGGACAGGGTATGCCTGAGAGTTGTACAAAATCCAGCTC<br>CTTACTCAGACAGAGTGATGGTTATGCCTAATTTATATGGTGATATTTGTCTGATATGTGTGCAGGACTGATCGGA<br>GGTTTAGGTCTTACTCCATCTGGTAACATAGGAAAAGATGCTTCTATATTCGAGGCTGTTCACGGAAGTGCTCCTGA<br>TATTGAAGGAAAGAACTTGGCCAACCCCACTGCTCTTTTGCTGAGTTCCTTGATGATGTTACGTCATATGAAGTTGA<br>CCCAGAAAGCTGACCTGATCGAGAAGGCAGTGCTTTCCACCATCGCTGAAGGAACAAGAACTAGAGACCTTGGAG<br>GTAAAGCTTCTACTCAACAATTTACTGACGAAATCCTGAAGAAATTATAA
CkPA	ATGAAACTTATGGAGAACATATTCGGTTTGGCCAAGGCTGACAAAAAAAAGATAGTCCTTGCTGAAGGTGAGGAG<br>GAGAGGAATATTAGAGCTAGTGAAGAGATTATTCGTGATGGTATTGCAGATATAATCTTGGTCGGTTCCGAGTCTG<br>TAATTAAGGAGAATGCTGCAAAGTTTGGAGTAAATTTAGCCGGCGTGGAGATCGTGGATCCTGAAACATCATCTAA<br>AACAGCTGGATACGCTAACGCATTTTACGAAATCAGAAAGAACAAGGGAGTTACTCTGGAAAAGGCAGATAAGA<br>TAGTCCGTGATCCTATCTACTTTGCTACTATGATGGTAAAACTGGGTGATGCTGATGGTCTTGTGTCTGGAGCCATC<br>CACACAACTGGTGACTTGTTGAGGCCAGGTCTGCAAATTGTGAAGACAGTACCAGGTGCTAGTGTGGTGTCTTCTG<br>TTTTTTTGATGTCCGTACCAGACTGCGAATACGGTGAGGATGGTTTCCTGTTATTTGCTGATTGCGCTGTGAACGTAT<br>GTCCAACTGCAGAAGAGCTGTCATCTATTGCAATAACAACTGCTGAAACCGCCAAGAATCTTTGCAAGATTGAAC<br>CAAGAGTTGCCATGTTGAGTTTTTCTACAATGGGTTCTGCCAGTCACGAGTTAGTTGATAAGGTCACGAAAGCAAC<br>AAAGTTGGCTAAGGAAGCAAGACCAGATTTGGATATTGATGGCGAGTTGCAACTGGACGCCTCTCTTGTTAAAAA<br>GGTTGCTGATCTTAAAGCACCAGGTTCTAAAGTGGCTGGAAAAGCTAACGTTTTAATCTTTCCAGACATTCAGGCA<br>GGTAACATCGGTTACAAGTTGGTTCAGAGGTTCGCAAAAGCCGAAGCTATTGGTCCTATCTGCCAAGGATTTGCTA<br>AGCCAATTAACGACTTGTCTAGAGGCTGTTCTGTTGACGACATTGTTAAAGTTGTGGCTGTTACTGCTGTTCAAGCT<br>CAAGCTCAGGGATAA
BbPKA	ATGACCAGTCCTGTAATCGGCACTCCATGGAAAAAACTGAATGCTCCCGTTTCTGAGGAATCTTTGGAAGGAGTAG<br>ATAAGTATTGGAGGGTTGCCAACTACTTGTCCATTGGACAAATCTACCTTAGATCCAATCCACTAATGAAGGCTCC<br>CTTTACCAGAGAAGACGTGAAGCATAGGTTGGTTGGTCATTGGGGAACCACCCCAGGTTTAAATTTCCTTATCGGA<br>CATATTAACAGATTCATCGCTGACCACGGTCAGAACACCGTTATTATTATGGGACCAGGACACGGTGGCCCAGCCG<br>GTACGTCACAGAGTTATCTAGATGGAACTTACACGGAAACTTTTCCAAAGATTACTAAAGATGAAGCTGGTTTACA<br>AAAGTTTTTTAGGCAGTTTTCATACCCAGGTGGAATTCCATCTCATTTTGCTCCAGAAACCCCAGGAAGTATACACG<br>AGGGTGGTGAATTGGGCTACGCATTGAGTCATGCCTATGGTGCTATCATGGACAATCCCTCACTATTCGTGCCAGC<br>CATTGTTGGAGATGGTGAAGCTGAAACCGGACCCTTGGCTACTGGTTGGCAATCAAACAAGTTAGTCAATCCTAGA<br>ACAGATGGTATCGTACTTCCTATTTTGCATTTGAATGGTTACAAAATTGCTAACCCAACTATCCTGAGTCGTATATC<br>AGATGAGGAACTACATGAATTTTTCCACGGAATGGGATACGAGCCTTATGAATTTGTCGCTGGTTTTGACGATGAA<br>GACCATATGTCCATTCACAGAAGGTTCGCTGAACTATGGGAAACGATATGGGATGAGATATGCGACATAAAAGCA<br>GCCGCACAAACCGATAATGTCCACAGACCATTTTACCCAATGCTAATTTTTAGGACTCCTAAGGGATGGACATGTC<br>CTAAATACATTGACGGTAAAAAAACAGAGGGATCCTGGAGAGCTCACCAAGTGCCATTAGCTAGTGCAAGAGAT<br>ACAGAGGCTCACTTCGAAGTCTTGAAAAATTGGCTAGAATCATACAAACCTGAAGAACTTTTCGATGCTAACGGA<br>GCTGTCAAGGACGACGTTTTAGCTTTTATGCCCAAGGGCGAACTACGTATTGGTGCCAATCCTAATGCTAACGGTG<br>GTGTTATTAGGGATGATTTGAAGCTGCCTAATCTTGAAGACTATGAAGTTAAGGAAGTCGCTGAATACGGACATGG<br>ATGGGGACAGTTGGAAGCTACAAGAACTCTGGGAGCCTATACCCGTGACATAATTCGTAACAATCCAAGAGATTT<br>CAGGATTTTTGGACCTGATGAGACCGCTAGTAACCGTTTACAGGCTAGTTACGAAGTTACTAATAAACAGTGGGAT<br>GCTGGCTATATTTCAGATGAAGTGGACGAGCATATGCACGTCTCTGGACAAGTTGTGGAACAACTGTCAGAGCATC<br>AAATGGAGGGTTTCTTGGAAGCTTATCTTTTAACTGGAAGGCATGGTATTTGGTCTTCTTACGAGTCTTTTGTTCATG<br>TTATTGACTCCATGCTTAACCAGCACGCTAAATGGCTTGAGGCTACTGTTAGAGAAATTCCATGGAGGAAGCCAAT<br>TGCCTCCATGAACCTTCTAGTGTCATCCCACGTTTGGAGACAGGACCACAACGGTTTTTCACACCAAGATCCAGGT

	GTTACTTCCGTTTTACTGAATAAGTGTTTCCATAATGATCACGTAATTGGAATTTACTTTGCCACTGATGCTAATATG<br>CTATTAGCCATCGCTGAAAAGTGTTACAAGTCTACTAATAAGATAAACGCTATTATTGCTGGAAAGCAACCAGCTG<br>CTACCTGGCTTACCTTAGATGAGGCCAGAGCTGAATTGGCCAAAGGTGCAGCTGCCTGGGATTGGGCATCCACTGC<br>AAAGAACAATGATGAAGCAGAGGTCGTGTTGGCTGCAGCTGGAGATGTTCCCACCCAAGAAATTATGGCAGCTTC<br>CGATAAGCTAAAAGAACTGGGCGTCAAGTTCAAAGTGGTTAATGTAGCCGATTTATTGAGTTTGCAGTCTGCAAAA<br>GAGAATGATGAAGCTCTGTCCGACGAAGAATTCGCTGATATATTTACCGCTGACAAACCTGTTCTGTTCGCATACC<br>ATTCATATGCTCACGATGTTAGAGGTCTTATCTATGATCGTCCAAATCACGATAATTTCAATGTGCACGGCTATGAG<br>GAAGAAGGATCAACAACGACTCCTTATGATATGGTTAGAGTCAACCGTATCGACAGGTATGAACTAACCGCTGAA<br>GCTTTGCGTATGATCGACGCTGATAAATACGCCGATAAAATAGATGAGTTGGAAAAATTTAGAGATGAAGCTTTCC<br>AATTCGCCGTCGATAAAGGCTACGATCATCCTGACTACACAGATTGGGTGTATTCTGGTGTTAATACCGACAAAAA<br>AGGCGCTGTGACCGCTACAGCTGCTACTGCAGGTGACAACGAATAA
RtACL	ATGTCCGCTAAGCCCATTAGAGAGTACGACGCCAAGTTGTTGTTGGCTTACCATTTGGCTAGAGCCCCAACTGCTG<br>GTTCTAAGGCTGTTGCTAGAGATGGTTTCCAGTCTCCAGAGGTTAAGGTTGCTCAAGTTTCTTGGGACCCAGAGACT<br>AACCAGGTTACTCCAGATGCTGCTTTGCCACACTGGGTTTTCACTGAGAAGTTGGTTGTTAAGCCCGACCAGCTGAT<br>CAAGAGAAGAGGTAAGGCTGGTTTGTTGGCCTTGAACAAGACTTGGGCTGAAGGTAAGCAATGGATCGCTGAAAG<br>AGCTGGTAAGCAGGTTCAGGTTGAAAAGACCACCGGTACTCTGAACAACTTCATCGTTGAGCCATTCTGCCCACAT<br>CCATCTGATGCTGAGTACTACATCTGCATCAACTCCGTTAGAGAGGGTGACGTCATCTTGTTCACTCACGAAGGTG<br>GTGTTGACGTTGGTGATGTTGATGCTAAGGCCTTGACCTTGTTGGTTCCAGTTGGTGGTGAATTGCCATCCAGGGAC<br>GAAATTAGATCCCAGTTGTTGAAGCACGTTACCGGTGCTGAAAGACAAGAGGCTTTGATCGACTACATCATCAGA<br>CTGTACTCCGTCTACGTCGACTTGCACTTTGCTTACTTGGAGATCAACCCATTGGTTGCCGTTGAGAACCCATCCAC<br>TGGTAAGACTGACATCTTCTACTTGGACATGGCTGCCAAGTTGGACCAGACTGCTGAATACGTTGTTGGTCCAAAG<br>TGGGCTATCGCTAGAGATCCATCCATTATCAACCCAGCTGCTGCTCCAATGTCCAACGGTAAGATTTCTGCTGATAA<br>GGGTCCACCAATGTTCTGGCCACCACCTTTTGGTAGAGACTTGACCAAAGAAGAGGCCTACATTGCTAAGCTGGAC<br>GGTTCTACTGGTGCCTCCTTGAAGTTGACTGTTTTGAACGCTGAGGGTAGAATCTGGACTATGGTTGCTGGTGGTGG<br>TGCTTCTGTTGTTTACTCTGATGCTATTGCTGCTCACGGTTTCGCTCACGAATTGGCTAACTACGGTGAATACTCTGG<br>TGCTCCAACTCAGACTCAGACTTACGAGTACGCTAAGACCATCTTGGACCTGATGACTAGAGGTACTCCAAATCCA<br>CAGGGTAAGCTGTTGTTCATCGGTGGTGGTATCGCTAACTTCACTAACGTTGCTGCTACCTTCAAGGGTATCATCAC<br>CGCCTTGAAAGAGTACCAGCACAGATTGCAAGAGCACAAGGTCAGAATCTTCGTCAGAAGAGGTGGTCCAAACTA<br>CCAAGAGGGTTTGAAGGCTATGAGACTGTTGGGTGAAACCTTGGGTGTTGAGATCCAAGTTTTCGGTCCAGAGACT<br>CACATCACCTCCATCGTTCCACTTGGTTTGGGTTTGATCAAGTCCGTTGACGACGCTTTGAAGGTTCCAGGTGCTAG<br>AGCTGCTGCAGATGCTACTGGTACTTTGACTCCAGTTCCAGGTTCTCCAAAGTCCAGAGCTGCTCAATTGCCAACTG<br>GTGCTTCCACTCCATCTAGACAACAACCACAGGACAACATCGTGAGCTTCTCTGACAAGGTTCATGCTCCAGACTC<br>TGGTAGACCATGGTACAGACCATTCGACGAGACTACCAGATCCATCGTTTACGGATTGCAGCCAAGAGCTATCCA<br>GGGTATGTTGGATTTCGACTTCGCTTGCGGTAGAGAGACTCCTTCTGTTGCTGCAATGGTTTACCCATTTGGTGGTCA<br>CCACGTCCAGAAGTTTTACTGGGGTACAAAAGAGACTCTGCTGCCAGTTTTCACTTCCATGAAGGAAGCTGTTGCC<br>AAGTGTCCAGACGCTGACGTTGTTGTTAACTTCGCTTCTTCCAGATCCGTCTACCAATCTACTTTGGAGGCTTTGGA<br>GTTCCCACAGATTAAGGCTATTGCCTTGATTGCCGAAGGTGTCCCAGAAAGACATGCCAGAGAAATCTTGCACCTG<br>GCCAAGAAGAAAGAGGTCATCATTATCGGTCCAGCCACTGTCGGTGGTATTAAGCCAGGTTGTTTCAGAATCGGTA<br>ACACCGGTGGTATGAACGAGAACATCTTGTCCTCCAAGTTGTACAGAGCCGGTTCCGTTGGTTACGTTTCTAAGTCT<br>GGTGGTATGTCTAACGAGCTGAACAACATCCTGTCCTTGACTACTGACGGTGCTTACGAGGGTATTGCTATTGGAG<br>GTGACAGATACCCAGGTACTACTTTCATCGACCACTTGTTGAGATACGAGGCTGACCCAAACTGCAAGATGTTGGT<br>TTTGCTGGGTGAAGTCGGAGGTGTCGAAGAGTACAGAGTTATCGAAGCTGTTAAGTCCGGTCAGATCAAGAAGCC<br>AATCGTTGCTTGGGCCATTGGTACTTGCGCTAAGATGTTCGCTACTGACGTTCAATTTGGTCACGCTGGTTCCATGG<br>CTAACTCTGACTTGGAAACTGCTGAGGCTAAGAACAACGCTATGAGAGCTGCCGGTTTTATCGTCCCACCAACTTT<br>CGAAGAATTGCCACAGGTTTTGGCCGAAACTTACCAGAAATTGGTCGGTGACGGTACTATCCAGCCAAAGCCAGA<br>AGTTCCACCACCACAAATTCCAATGGACTACAACTGGGCTCAGACCCTTGGTATGGTTAGAAAGCCTGCTGCTTTC<br>ATCTCCACTATCTCTGACGAGAGAGGTCAAGAGTTGTTGTACGCTGGTATGCCAATCTCCAAGGTTTTCGAAGAGG<br>ACATCGGTATTGGTGGTGTCGTTTCCTTGCTGTGGTTCAAGCGTAGATTGCCAGCTTACGCTACCAAGTTCTTGGAG<br>ATGGTTCTGATGTTGACTGCCGATCATGGTCCAGCTGTTTCTGGTGCTATGACTACTGTTATCACCACTAGAGCCGG<br>TAAGGACTTGGTTTCTTCCTTGGTCGCTGGTTTGCTGACTATCGGTGATAGATTTGGAGGTGCTTTGGACGGTGCTGC<br>TCAAGAATTCACTAGAGCTTTCGAAGCTGGTCTGACCCCAAGAGAATTCGTTGACTCTATGAGAAAGGCTAACAA<br>GCTGATCCCAGGTATCGGTCACAAGGTTAAGTCTAAGGCTAACCCAGACAAGAGAGTCGAGCTGGTTAAGAACTA<br>CGTGTTCAAGCACTTCCCATCCGCTAAGTTGTTGGAGTACGCTTTGGCCGTTGAGGACGTTACTTCTGCTAAGAAGG<br>ACACCTTGATCCTGAACGTTGATGGTGCTATCGCCGTTTCCTTCGTTGACCTGTTGAAGAACTCCGGTGCTTTCACTG<br>CTGAAGAGGCTGCTGAGTATATGAAGATCGGAACCTTGAACGGTCTGTTCGTCTTGGGTAGATCCATTGGTTTCATT<br>GCCCACCACCTGGACCAGAAGAGATTGAAGCAACCACTGTACAGACACCCAGCTGACGACATTTTCATCCAGCCA<br>TTCAACACCGACAGAATCTTGGTCCAGCAGAGACAGTAA
MmME	ATGGAACCTAGAGCACCAAGAAGAAGGCACACTCACCAAAGAGGTTACCTGTTGACTAGAGATCCACACCTGAA<br>CAAGGACTTGGCTTTCACCTTGGAAGAGAGACAGCAGTTGAACATCCACGGTTTGTTGCCACCATGCATCATCTCC<br>CAAGAGTTGCAGGTCCTGAGAATCATCAAGAACTTCGAGAGACTGAACTCCGACTTCGACAGATACTTGTTGTTGA<br>TGGACCTGCAGGACAGAAACGAGAAGTTGTTCTACTCCGTCTTGATGTCCGACGTCGAGAAGTTCATGCCAATCGT<br>TTACACTCCAACCGTTGGTTTGGCTTGTCAGCAATACTCTCTGGCCTTCAGAAAGCCTAGAGGTCTGTTCATTTCCAT<br>CCACGACAAGGGTCACATTGCCTCTGTTTTGAACGCTTGGCCAGAGGACGTTGTTAAGGCTATCGTTGTTACTGACG<br>GCGAGAGAATCCTTGGTTTGGGTGATTTGGGTTGCAACGGTATGGGTATTCCAGTTGGAAAGTTGGCCTTGTACACT<br>GCCTGTGGTGGTGTTAACCCACAGCAGTGTTTGCCAATCACTTTGGACGTTGGTACTGAGAACGAGGAATTGCTGA<br>AGGACCCACTGTACATCGGTCTGAGACACAGAAGAGTTAGAGGTCCAGAATACGACGCTTTCCTGGACGAGTTTA

	TGGAAGCTGCTTCTTCCAAGTACGGTATGAACTGCTTGATCCAGTTCGAGGACTTCGCTAACAGAAACGCCTTCAG<br>ACTGCTGAACAAGTACAGAAACAAGTACTGCACCTTCAACGACGATATCCAGGGTACTGCTTCTGTTGCTGTTGCT<br>GGTTTGTTGGCTGCTTTGAGGATCACCAAGAACAAGTTGTCCGACCAGACCGTCTTGTTTCAAGGTGCTGGTGAAG<br>CTGCATTGGGTATCGCTCATTTGGTTGTGATGGCCATGGAAAAAGAGGGTCTGTCCAAAGAGAACGCCAGAAAGA<br>AGATCTGGTTGGTCGACTCCAAGGGTTTGATCGTTAAGGGTAGAGCTTCCCTGACCGAAGAGAAAGAAGTTTTCGC<br>TCACGAGCACGAAGAGATGAAGAACTTGGAAGCCATCGTCCAGAAGATCAAGCCAACTGCTTTGATCGGTGTTGC<br>TGCTATTGGTGGTGCTTTCACTGAGCAGATCTTGAAGGATATGGCCGCTTTCAACGAGAGGCCAATCATCTTCGCTT<br>TGTCTAACCCAACTTCCAAGGCTGAGTGTTCCGCTGAGCAATGTTACAAGGTTACTAAGGGTCGTGCTATCTTCGCC<br>TCTGGTTCTCCATTTGACCCAGTTACTTTGCCAGACGGTAGAACTTTGTTCCCAGGTCAAGGTAACAACTCCTACGT<br>TTTCCCAGGTGTTGCCTTGGGTGTTGTTGCTTGTGGTTTGAGACACATTGACGACAAGGTGTTCTTGACTACCGCTGA<br>GGTTATTTCCCAACAGGTTTCCGACAAGCACTTGCAAGAAGGTAGACTGTACCCACCACTGAACACCATCAGAGG<br>TGTTTCTTTGAAGATCGCCGTCAAGATCGTTCAGGACGCCTACAAAGAAAAGATGGCCACTGTTTACCCAGAGCCA<br>CAGAACAAAGAAGAGTTCGTTTCTTCCCAGATGTACTCCACCAACTACGACCAAATCTTGCCAGACTGTTACCCAT<br>GGCCAGCTGAGGTTCAAAAGATCCAGACTAAGGTTAACCAG
CaGDH	ATGTTCGAGAACATCTCCTCCAACGGTGTCTACAAGAACCTGTTCGATGGAAAGTGGGTTGAGTCCAAGACCAAC<br>AAGACTATCGAAACTCACTCCCCATACGACGGTTCCTTGATTGGTAAGGTTCAGGCCTTGTCCAAAGAAGAGGTTG<br>ACGAGATTTTCAAGTCCTCCAGAACTGCCCAAAAGAAGTGGGGAGAAACTCCAATCAACGAGAGGGCCAGAATC<br>ATGAGAAAGGCTGCTGATATCTTGGACGACAACGCTGAGTACATTGCCAAGATTCTGTCCAACGAGATCGCCAAG<br>GATCTGAAGTCCTCTTTGTCCGAGGTTAAGAGAACTGCCGACTTCATCAGATTCACTGCCAACGAAGGTACTCACA<br>TGGAAGGTGAGGCTATCAACTCTGACAACTTCCCAGGTTCCAAGAAGGACAAGTTGTCCCTGGTTGAGAGAGTCCC<br>ATTGGGTATCGTTTTGGCTATCTCCCCATTCAACTACCCAGTTAACTTGTCCGGTTCCAAGGTTGCTCCAGCTTTGAT<br>TGCTGGTAACTCCGTTGTTTTGAAGCCATCCACTACTGGTGCTATTTCCGCCTTGCATTTGGCCGAAATTTTCAACGC<br>TGCTGGTTTGCCAGCTGGTGTTTTGAACACTGTTACTGGTAAGGGTTCCGAGATCGGTGACTACTTGATTACCCACG<br>AAGAGGTCAACTTCATCAACTTTACTGGTTCCTCCGCCGTCGGTAAGCACATTTCTAAGATCGCTGGTATGATCCCC<br>ATGGTGTTGGAACTTGGTGGTAAGGATGCTGCTATTGTCTTGGAGGACGCTAACTTGGAGACTACTGCTAAGTCTAT<br>CGTTTCCGGTGCTTACGGTTACTCCGGTCAAAGATGTACTGCCGTTAAGCGTGTTCTGGTCATGGATAAGGTTGCCG<br>ACGAATTGGTTGAGCTGGTCACCAAGAAGGTCAAAGAGTTGAAGGTCGGTAACCCATTCGACGACGTTACTATCA<br>CTCCACTGATCGACAACAAGGCTGCCGACTACGTTCAGACTTTGATTGACGACGCTATCGAGAAGGGTGCCACTTT<br>GATCGTTGGTAACAAGAGAAAAGAAAACCTGATGTACCCCACCTTGTTCGACAACGTCACTGCTGACATGAGAAT<br>CGCTTGGGAAGAACCATTCGGTCCAGTCTTGCCAATCATCAGAGTCAAGTCTATGGACGAGGCTATTGAGCTGGCT<br>AACAGATCTGAGTACGGTCTGCAATCCGCTGTTTTCACTGAGAACATGCACGACGCTTTCTACATTGCTAACAAGC<br>TGGACGTTGGTACTGTCCAGGTTAACAACAAGCCAGAAAGAGGTCCAGATCACTTCCCATTCTTGGGTACTAAGTC<br>CTCCGGTATGGGTACTCAGGGTATCAGATACTCCATCGAGGCTATGACTAGACACAAGTCCATCGTCTTGAACTTG<br>TAA
BaFAS	ATGACCATCGGTATCTCCAACCACAGACTGGTCAACAGATTGGAGTCTGAGAAGTTCGCCTTGTCCTTCTCAGGTC<br>AAGGTTTCTCTTGGCTGCCAACCTTGAGAACTGTTATCGCTGGTGGTAACGGTGCTCAAGTTGCTGGTGCTGTTGAT<br>GCTGCTGGTAAGTTGTTGGAACCAGTTGCTGACGAATTGGTTGCTGCTTTGCCACACGGTTTCGATCCAGTTGAATG<br>GGCTGGTAACGCTGAAGATCCAGCTTGGGATTTGTCTGACTCCTCCATTTCTACCCCAGGTATCGTTGTTGCTCAGT<br>ACGGTTTGATCGAGTCCTTGAAGGCTCAAGGTTTGGACCCAGCTGATGCTGTTGCTCATATTGGTCACTCTCAAGGT<br>GCTCTGGCTACTTACATTTCTTCCGGTAGAGCACAAGCTGCTGAGGTTATTGCTTTGGCTCAGTTGATCGGTGTTGCC<br>ATCTCTAAGACTGCTAGAGCCACTGGTTTGATCAGAATCGCTGCTGGTTCTCCAATGGTTTCCATTGCCGGTATTTCC<br>AGAGAGCAGTTGGCTAAGGTTTTGGCCAAGACTGGTGTTGACTCCGTTATCGGTCTGCAAAACAACCACAACACCT<br>TCGTTGTTACTGGTTCCCCAGAGGACAACAACAAGGTTATTGAGGCCATCGAGAAGTTGGCTGCTAAGGACGCTAA<br>GGCTATCGAGAACAAAGAAAGAGGTGGTAACCCATTCGCTCCAAGAATCAACGCTTTGCCAGTTCAATCCGGATA<br>CCACCATCCAAAGATGTCTGCTGCTGTTGAGATGGTTACTTCCTGGGCTGAGAGAATCGGTTTGGATAAGGATTTG<br>GCTCACGACGGTGCTGAGGCTGTTTTGACTGAAATCGTTAACTGGCCAGCTACTGTTCAGTCTGCCGTAGATGCTGG<br>TGTTACTTGGATTTTGGAATGCGGTCCAGAGAAAGGTATGGCTGCTTTGACTAAGGCTGTTGTTGCTGGTCAAGGTA<br>TCGGTGTTCAGGTTGTTTCTACTGACGAAGGTCAAGCTGCCTTGTTCGATGTTGGTCAAGCTCCAGAATTGCCAGTC<br>AACTTCGCTCAATTCGCCCCAAAGGTTTTCGACGACAAGGGTAAGACTAGACTGTCCACTAGATTCACCGAGTTGA<br>CCGGTTACTCCCCAGTTGTTTTGGCTGGTATGACTCCATCTACTGTTGACCCTGCTATCGTTGCTGCTGCTGCAAATG<br>CTGGTTTTTGGGCTGAATTGGCTGGTGGTGGTCAAGTTACTGACGCTATTTTGAACGACTCCTTGGAGAGATTGGAG<br>GACATGTTGAACCCAGGTATTAACGCTCAGTTCAACGCCATGTACTTGTCCCCAAAACAATGGCGTGCTCAGATCG<br>AGGGTAGAAGATTGATTCCAAGAGCCAGAGCTAACGGTGCCTCCATTAACGGTGTTATTTGTTCCGCTGGTATCCC<br>ACCACACGAAGAAGCTATCGCTTTGGTCAGACAACTGCAAGAGGATAACATCCCTTGGGTTGCTTTTAAGCCAGGT<br>GCCGTTAGACACGTTCACCAGGTTCTTGCTATTGCCGATGATTTGCCAGACACCACCGTTATCATGCAGGTTGAAGG<br>TGGTAAAGCTGGTGGTCATCACTCCTGGGAAGATTTGTCCTCTTTGCTGACTGAGACTTACGCCGACATTAGAGAG<br>AGAGACAACGTCGTTTTGATGGCTGCCGGTGGTATTGGTGCTCCAGAGAGAGGTGCTCAATACTTGACTGGTGAAT<br>GGTCCAAGGTCTACGGTTTGCCAGCTATGCCAGTTGACGCTATCATGATTGGTACTGCTGCTATGGCTACCAAAGA<br>GTCCACTGCTTCTGAATCCGTTAAGCAGGCTTTGGTTGCTACCCAAGGTCTTGAGGATATTCCAGGTGGTGGTTGGG<br>TTCCAGCCGGTGGTGCTAGAGATGGTATTGCTTCTGGTAGATCCCAGTTGGGTGCTGACATTCACGAGATCGATAA<br>CACTTTTGCTAAGGCCGGTAGATTGCTGGATGAAGTTGCCGGTGATGCAGATGCAGTTGCTGCTAGAAGAGAAGAG<br>ATTATCGCCGTGATTGCTGGTACTGCCAAGCCATACTTCGGTGACATTAACGCTATGACCTACGCTGAGTGGTTGGC<br>CAGATACGTTGAAGTTTCCTACATGGGTTCTTGGGTTGACTTCTCCTGGGCTAGAAGATTCGAGCAGATGATTGCTA<br>GAGCTGAGGCCAGATTGACTGAAGCTGATCACGGTGAGTTCACCCCATTGGTTACTGTCGATGCTGAACATCCAGA<br>GAGGGCTATTGCTGACGTTGTTGCAGCTTATCCTGCTGCTGAAAAGCACACTGTTATTGCCTCTGACGAAGCCTGGT<br>TCGTTGACTTGTTGAGAGGTAACGGTAAGCCACCAGCCTTCGTTCCAGTTATTGATTCCGACGTTAGAAGATGGTGG

CGTCAAGACTCTTTGTGGCAAGCTCATGACGAGAGATACACTGCTGAGCAGGTTGGTATTATCCCAGGTACTACTG
CCGTTGCTGGTATCACTAAGGCTAACGAACCTGTTGCAGAGTTGTTGGCTAGATTCGAAGCTGCTGCCGTTGAACA
AGCCTTGCAATCTGAAGATACTGAGAAGGCTGAACTGGCTAACTCCGCTTTGGAAAGAGTTTTGGCAGCTCCTTCT
GTTGAGTGGGCAGGTAGACAAAGACCAAATCCAGTTGCTCAGGTTTCCGAGTCTTGGACTGTTGACTCTCAAGCTA
AGACCGCTTCTACTGAAGGTGCTGAGTTGGTTACTATTGACGAGCACACCGTTGAGTTGTCCGTTGCTTTGACAGGT
TCTGTTCCAGGTGCTACCTTGCAACTGCAAATCACTGTTCCTGCTGACGCTCCATTGTCCGCTACTCCAATTATTACT
GCTCAGGCTGCCGAAGAATCTATGGCAGCTTTAGCAGCTGTAGCTGCTGGTGGAACTTTGCCAGAGGTTACTGATG
GAACTGCCGTTTGGAAAACTACTTTGGACTCCGCTGCTTTGTCCGACTATGCCGGTGTAACTTCTGGTTACTTGGCTT
CCGCTGACACCAACTCTGCTATTGCTCCAGATGTTATCGTTGGTAGAGCTTGGCCAGCTGTTTTCGCTGTCATTGCCA
ATTCCGTTATTCCTGGTTCCGACTCCGCATTGGTTGTTGAGGGTTTGTTGAACTTGGTCCACTTGGAACACGACGTGA
GATTGCATCAGGCTTTGCCAACAGAAGCTGTTGACTTGACTGTTACCGCTAGAGGTGGTGAGGTTAGAGATACTGC
TTTGGGTAGAGTTGTCGAGGTCGCTGTTGATATTGCTATCGCTGAAGCCGAGGATACCGTTTTGGCTACCTTGAAAG
AGAGATTCGCCATCGTCGGTAGAACTGGTGAGCAAGAATTGGAACCTGCTACTTCTACTGCCGAGATTACCAATAC
TCCAGCCTCCTTCAGACACCAGTTGAGAGTTACTGCTCCAGCTTCTATGCACCCTTTCGCTGTTGTTACCGGTGACA
GAAACCCAATTCACGTTAGTGCTGCCGCTGCTAGATTGGCAGGATTGGAAAATGGTGTTATCGTCCACGGTATGTG
GACTTCCGCTATTGGTCAATTGGCCGCTGGTTTCGATGGTTTGGAATTGGCCGAATGGTCCGCTAACATGTTGTCTC
CAGTTTTGCCAGGTGCTGAAGTCACCTTCATCGTTGAAAGAACCGGTGTCGATTCCAGACCAGGTAGAGGTGAAGT
TAGAACCGTTACTGCTTCCGTTGGTGATGACGTTGTGTTGCAAGCTACTGCTACAATGGCTGCCCCAAGAACTTTTT
ACGGTTTTCCAGGTCAGGGTATTCAGTCCCAAGGTATGGGTATGGAATCCTACGCTTCTTCATCTGCTGCCAGAGAA
GTTTGGGAAAGAGCTGACGCACACACTAGAGAAAAGCTGGGTTTCTCCATCTTGGAGATCGTCAGAAACAATCCA
CCACTGGTTGTTGTGGACGGTGAAGAGTTTACTCACCCAGACGGTACTTTGTTCTTGACCCAGTTTACCCAAGTTGC
TATGGCCACTTTGGGTTGTGCTCAAATTGCCGAGTTGGCTGAAGCTGGTGTCAAGTCTCAAGACACTTACTTCGCTG
GACACTCTGTCGGTGAGTATAACGCTTTGGCTGCTTACGCTCAAGTCTTGTCTTTGGAAGCCGTTGTCGAGATCGTTT
ACGCTAGAGGATTGACCATGCACAGATTGGTTGACAGAGATGCTGAGGGTAACTCCAACTACGGATTGGCTGCTGT
TAGACCAAACAAGATCGGTATTAGAGCCGAGGACGTTTTCGACTACGTTGCCCAAGTTTCTCAAACCAGAGGTGA
GTTCCTGGAAATCGTCAACTACAACATTGCTGGTGTCCAGTACGCTGTTGCTGGAACTAGAGCTGGTTTGGCTGCAT
TAGCTGCTGATGCAGAATCTAGAGCACCAGGTATGAGATCCTTCATCATGATCCCTGGTATCGACGTTCCATTCCAC
TCTTCTCATTTGCTTGGTGGTGTGGACAACTTCAGAACCCACTTGGATATGTTGATCCCAGACGACGTTGACCTGGA
CATTTTGAGAGGTCGTTACATCCCTAACTTGGTCGCTAGACCATTCGAGTTGACTCGTGAGTTCGTTGAAGCTATGG
CCGATGTTGTTGATTCCACCTACGTCAACGACATCTTGGCTGATTTCGACAAGGCTACTGATGATCCCGCTAAGATG
GGTAGAACTTTGTTGATCGAATTGCTGGCCTGGCAGTTCGCTTCTCCAGTTAGATGGATTGAGACTCAGGACTTGTT
GCTGTCTGCTCCATCTGTTTCTGCTGGTGCAGGTACAGCTGGATTGGGTGTTGAAAGATTCGTTGAGATTGGTGTCG
GTTCTTCCCCAACTTTGGCAAACATGTTGGGTCAGACCTTGAGATTGCCTCAATACGCTGGTAACCCTATCGAGGTT
TTGAACGTTGAGAGAGATAGAGCCACCGTTTTTGCTGAGGATGCCTTGGAAAGACCTGTTGAGGATTCTTCTGAGT
CCTCTACCGGTGTTGCTGAAGCAGCTACTGCAACAGCTGAAGAAGTTTCCCAAGCTAACGCAGCTCCAGCAGCTGC
ACCAGAGCCAGTTGTTGCACAACCAGCTACTCCCGCTCCTGCCGCCGGTGGAACTACTCCAGATGATAAGGCTTTT
TCTGCTGCCGACGCTACCGAGATGTTGATTGCTATTTGGACCAAGGTCAGACCAGACCAAATGGGTGCTGCTGACA
CTATTGAACTGTTGGTTGAGGGTGTCTCCTCCAGAAGAAATCAGTTGTTGTTGGACCTGGGTGTCGAGTTTGGTTTG
GGTGCTATTGAAGGTGCCCACGCTGAAATTTCTGCCCTGAAAGAACAAGTTTCCGGTATGGCCAAGGGTTACAAGG
CTTTTGGTCCTGTTTTGTCCGCTCAGGTTGCTGAATCCTTGAGAAGGTTGACTGGTCCAGCTGGTAAGAAGCCAGCT
TACATTGCTCAGAGAGTTGCCGATACTTGGGGTTTAGGTGCTGGATGGGTTGATAGAGTTACAGCTGAACTTGTCTT
GGGTGCAAGAGAAGGTGCTTCCTTACGTGGTGGTGATTTGGCTTTATTGGCTCCAGCTAACCCTGCTACTACAGCCG
AATTGGATGCTTTGATTGATGCCGCTGTAGACGCAGCTGGTGCTAAGGTTGGAATCGTTATTTCTAAGCCATCCGCT
TCCGGTGCTGCTCAAGGTGGTGTAGTTGATTCAGCTGCTTTGGACGCTTACTCCGAGAAGATGAACTCAGCTTTGGC
TAACACTGCCAGAACTTTGCTGTCTCAATTGGGTCAGCAAGCCCCAACTACTGAGTTTTCTGAGGCTGATGATAAC
GCCGCTGTGGTTGATTTGGTTACCTCTGAATTGGGAGCTGACTGGCCTAGATTGGTTTCCCCATCCTTTGATGCTGAC
AAGACCGTTCAATTGGACGACAGATGGGCTTCTGCTAGAGAGGACTTGGTTAGATTGTCCTTGGGTGAGATCGAGG
ATTTGGACATTACTGGTGCTGGTGAAGAGGCAGCTGCAATGGCAACTTACTTGGGTCTTGATGAGCAAGCTGCCCA
AGCTAGAGTTGCTGCATCCACTTTGGATCATGACGGTGAGGTTGCTGTTGTCACTGGTGGATCTCCAGGTTCTATTG
CTTCCGAGATTGTCGCCAACTTGCTTAGAGAGGGTGCTACCGTTATTGCTACCACTTCCAGATTGGGTCACGACAGG
TTGGAGTTCTACAAGAACTTGTACAGAACCTCCAGACGTGGTAACGCCCAATTGTGGATCGTTCCTGCTAACTTGA
ACTCCTTCGCTGATCTGGACGCTTTGATCGATTGGATCGGTACTGAACAGACCGTTACCGTTGGTGGTTCTTCCAAG
TTGGTTAAGCCAGCATTGGTCCCAACTCTGTTGTTTCCATTTGCTGCTCCAAGAGTCCAGGGTACTTTAGCTGACGCT
GGTGCTCCTGCTGAAGCCCAAATGAGATTGCTTTTGTGGTCCGTCGAAAAGTTGATCGCTGGTTTGTCTCAGCTTGG
TACTGGTACTCACGTTGGTCAGAGATTGCACATCGTTTTGCCTGGCTCTCCAAACAGAGGTAGATTCGGTGGTGATG
GTGCTTACGGTGAATCCAAGGCTGCTCTTGATGCTTTGGTTACAAGATGGCACGCTGAACCAGTTTGGGGTGCTAG
AACTTCTATCGTTCACGCTCATATCGGTTGGGTTAGAGGAACTGGATTGATGGGTGGAAACGACCCTTTGGTTGAC
GCTGTTGAGGCTAAGGGTGTTAGAACCTTCTCCACTGAAGAGATGGCTTCCCAATTGGTTGGTGAAGGTGCATCTA
CCTCCAAGCGTGATGCAGCAGCTCATGCTCCAGTTACATTGGATCTTACTGGTGGTTTGGGAGAAGCCGACATCAA
CTTGCCAGAATTGGCTAGGTCTGTTTCCGTTCAACCTGTGGCTGAAGATGTTGAAGAGGTTGCTTCCTTGAGAGCCC
TGCCAAACATCAGAGAAAGAATCGAGTGGACTACCCCAGATTTCGCCGGTGTTAATCAAAAGCTGGACGACATGG
TTGTCATCGTTGGTGCCGGTGAAATTGGACCAGTTGGTTCCTCTAGAACCAGATTCGAGGTTGAAATGACCGGTGA
TTTGACTGCCGCTGGTGTCGTTGAATTGGCATGGACTACAGGTTTGATTGCTTGGGATGACGCTTCTGGTGCTTGGTT
TGATGCCGAAGGTGAAGAGATTGCTGAAGAGGACATCTACGACAGATTCCACGACGAAGTCTTGGCTAACGTTGG
TGTGAGAAAGTACCACGATGACTACGGTCCTAAGATGCCAATGGTTGACAACTTGGCTCCTGAGCTGACCACTATC
TATTTGGAGAGGGACATGACTTTCGCCGTTGCCGACGAAGAAACTGCTAGAACATTCGTTGCTGATGTCGAGGGTG

	CCACTGCTCATTTTGATGGTGAAGAATGGCAGGTTACTAGACCAGCTGGCGCAGAAGTTAGAGTTCCTAGAAGAGT<br>TGCCATGACCAGATTTGTCGGTGGTCAAATTCCACAGGGTTTTGACCCAGGTGTTTACGGTATCCCAGCTGACATGA<br>TTGACAACCTGGACAGAGTTGCCTTGTGGAACCTTGTCTGTACCGTTGATGCATTTCTGGCAGCCGGTTTTTCACCA<br>GCTGAATTGCTAGCTCATGTTCACCCAGCCAGAGTCTCTTCCACTCAAGGTACTGGAATGGGTGGTATGCAATCCA<br>TGAGATCCCTGTACTTGGACAAGTTGTTGCGTCAGCCTAGACCTAACGACATTCTGCAAGAAGCCTTGCCAAACGT<br>TGTCGCTGCTCACGTTATGCAGTCTTACGTTGGTGGATACGGTCAGATGGTTCATCCAGTCGCTGCTTGTGCTACAG<br>CAGCTGTTTCTGTTGAAGAAGGTATGGACAAGCTGAGGTTGAGAAAGGCTGACTTTGTTGTTGCCGGCGGTATCGA<br>TGACTTGTCCATTGAAGGTATCACCGGTTTCGGTGATATGGCCGCTACTGCTGATTCTGGTGAAATGGAAGCTAAG<br>GGCATCGACCACAAGTACTTCTCTAGAGCCAACGACAGAAGAAGAGGCGGTTTCATTGAATCCGAAGGTGGTGGA<br>ACCATCTTGCTGGCTAGAGGTTCATTAGCAGCAGAATTGGGTCTGCCAGTTCTGGGTGTTGTTGGTTTCGCTGAATC<br>TTTTGCTGATGGTGCCCACACCTCTATTCCAGCTCCAGGTCTTGGAGCTTTGTCAGCTGCTAGAGGCGGAGCTGATT<br>CAAGATTGGCACAAGGATTGGCAGAACTGGGTGTTTCCGCTAACGAGATCGCTGTTATTTCCAAGCACGACACTTC<br>CACTAACGCCAACGATCCAAACGAATCTGACCTGCACGAAAGAATTGCTGCCTCTATGGGTAGAGATGCAGGTAA<br>CCCTTTGTTCATCATCTCCCAAAAGACCCTGACTGGTCACGCAAAAGGTGGTGCAGCCGCTTTTCAATTGGTCGGTT<br>TGACTCAGGTTCTGAGAAACGGTGTTTTGCCACCAAACAGATCCTTGGATTGTGTTGACCCAGCCTTGAGACAACA<br>CACTCACTTGACCTGGTTGAGAGAACAGTTGGACTTGAGAGCTACTCCACCAAAGGCAGGTTTGGTCACTTCTCTT<br>GGTTTCGGTCACGTTTCTGCCTTGGTTGCCGTTGTTCATCCTGGTGCATTTGTTGAAGCTCTGAGAGCTGAAAGAGGC<br>GCAGAGGCTGCTGCTCAGTGGCAACAATCTGCTAATGCTAGAGAAGCTGCCGGATTGCAGAGATTGACTTCTGCTA<br>TTTCTGGTGGTCCAGCCTTGTACGAACGTCCTGTTGATAGAAATCTTGGTGGCACTGGTGACGTCGTCAAAGAGAG<br>AGAAGCAGCCGTTTTGTTGGACGATGGTGCAGCAGCCAGATCAAGACCAAGAGCTGAACACTTCAGAAGGATCAG<br>AAAGGCCGCTGTTGTCTTGGACAACAGAGAAGCTATGACCGTCGGTGTTGATCTGGTTCACATTCCAGCTTTGCCAT<br>CCAACTGTAGAGCTTTGGTGAGACTGCTGTCCAAGTGTTTCAGACGTTGGAACGTTGTCATGTTGAAGCGTGCTGTT<br>ACCTTGCAGAGAATGTTGAGAATCAGAGCCCTGAGAGTTCACGGTTTGTCTACATGGTTAGGTGGTGGCAGACAAA<br>AGAAGAGATCCTCTCGTAGAGGTAGGAAGAGATCAACTGCTTCCCACCAATAA
hFAS	ATGGAAGAGGTTGTTATCGCTGGTATGTCCGGTAAGTTGCCAGAATCCGAAAACCTGCAAGAGTTCTGGGACAACT<br>TGATCGGTGGTGTTGACATGGTTACTGACGACGACAGAAGATGGAAGGCTGGTCTTTACGGTTTGCCAAGAAGATC<br>CGGAAAGTTGAAGGACCTGTCCAGATTCGACGCTTCCTTTTTCGGTGTTCACCCAAAGCAAGCTCACACTATGGAC<br>CCACAATTGAGATTGCTGTTGGAGGTTACTTACGAGGCCATCGTTGACGGTGGTATTAACCCAGATTCCTTGAGAG<br>GTACTCACACTGGTGTTTGGGTTGGTGTTTCTGGTTCTGAAACTTCCGAGGCCTTGTCCAGAGATCCAGAAACTTTG<br>GTTGGTTACTCCATGGTTGGTTGTCAGAGGGCTATGATGGCTAACAGACTGTCATTCTTCTTCGACTTCCGTGGTCCA<br>TCCATTGCTTTGGATACTGCTTGTTCCTCTTCCTTGATGGCCTTGCAAAACGCTTACCAGGCTATTCACTCTGGTCAA<br>TGTCCAGCTGCTATCGTCGGAGGTATCAACGTTTTGTTGAAGCCAAACACCTCCGTCCAGTTCTTGAGATTGGGTAT<br>GTTGTCTCCAGAGGGTACTTGTAAGGCTTTCGACACTGCTGGTAACGGTTACTGTAGATCCGAAGGTGTTGTTGCCG<br>TCTTGTTGACCAAGAAGTCCTTGGCCAGAAGAGTTTACGCCACTATCTTGAACGCTGGCACTAACACCGACGGTTT<br>CAAAGAACAGGGTGTCACTTTCCCATCCGGTGACATTCAAGAGCAGTTGATCAGATCCTTGTACCAGTCTGCTGGT<br>GTTGCTCCAGAATCCTTCGAGTACATTGAAGCTCACGGTACTGGTACTAAGGTCGGTGATCCACAAGAGTTGAACG<br>GTATCACTAGAGCTTTGTGCGCTACCAGACAAGAGCCTTTGTTGATCGGTTCCACCAAGTCTAACATGGGTCATCC<br>AGAACCAGCTTCCGGTTTGGCTGCTTTGGCTAAGGTTTTGTTGTCTTTGGAGCACGGTTTGTGGGCTCCAAACTTGC<br>ATTTCCACTCTCCAAATCCTGAGATCCCAGCTTTGTTGGACGGTAGATTGCAGGTTGTTGATCAGCCATTGCCAGTC<br>AGAGGTGGTAACGTTGGTATCAACTCTTTCGGTTTCGGTGGTTCCAACGTCCACATTATCTTGCGTCCAAACACTCA<br>ACCACCACCAGCTCCAGCTCCACATGCTACTTTACCAAGATTGCTGAGAGCCTCCGGTAGAACTCCAGAAGCTGTT<br>CAAAAGTTGTTGGAGCAGGGATTGAGACACTCTCAGGACTTGGCTTTCTTGTCCATGTTGAACGACATTGCTGCTGT<br>TCCAGCTACTGCCATGCCTTTTAGAGGTTACGCTGTTCTTGGTGGTGAAAGAGGTGGTCCAGAGGTTCAACAAGTTC<br>CAGCCGGTGAAAGACCATTGTGGTTCATTTGTTCCGGTATGGGTACTCAGTGGCGTGGTATGGGTTTGTCCTTGATG<br>AGACTGGACAGATTCAGAGACTCCATCTTGAGATCCGACGAGGCTGTTAAGCCATTCGGTTTGAAGGTTTCCCAGT<br>TGTTGTTGTCCACTGACGAGTCTACTTTCGACGACATCGTCCATTCTTTCGTTTCCCTGACTGCTATCCAGATCGGTTT<br>GATCGACTTGTTGTCCTGCATGGGTCTTAGACCAGATGGTATCGTTGGTCACTCCTTGGGTGAAGTTGCTTGTGGTTA<br>TGCTGACGGTTGTCTGTCTCAAGAAGAGGCTGTTTTGGCTGCCTATTGGAGAGGTCAGTGTATCAAAGAGGCTCAC<br>TTGCCACCAGGTGCTATGGCTGCTGTTGGTTTGTCTTGGGAAGAGTGTAAGCAGAGATGTCCACCTGGTGTCGTTCC<br>AGCTTGTCACAACTCTAAGGACACTGTTACTATCTCCGGTCCACAGGCTCCAGTTTTCGAGTTCGTTGAGCAACTGA<br>GAAAAGAGGGTGTTTTCGCCAAAGAGGTTAGAACAGGCGGTATGGCTTTTCACTCCTACTTCATGGAAGCTATCGC<br>CCCACCTTTGTTGCAAGAGCTGAAGAAGGTTATCAGAGAGCCAAAGCCAAGATCCGCTAGATGGTTGTCTACTTCC<br>ATTCCAGAGGCTCAATGGCATTCTTCCTTGGCTCGTACTTCTTCCGCTGAGTACAACGTCAACAACTTGGTTTCCCC<br>AGTCTTGTTCCAAGAGGCTTTGTGGCATGTTCCAGAGCACGCTGTTGTTTTGGAAATTGCTCCACACGCTCTGTTGC<br>AGGCCGTTTTGAAGAGAGGTTTGAAGCCATCCTGCACTATCATCCCACTGATGAAGAAGGACCACAGAGACAACT<br>TGGAGTTCTTCCTGGCTGGTATTGGTAGACTGCACTTGTCCGGTATTGACGCTAACCCAAACGCTTTGTTCCCACCA<br>GTTGAATTTCCAGCTCCTAGAGGAACTCCATTGATCTCCCCATTGATTAAGTGGGACCACTCTTTGGCTTGGGATGT<br>TCCTGCTGCTGAAGATTTCCCAAACGGATCTGGTTCTCCATCCGCTGCCATCTACAATATTGACACTTCTTCTGAGTC<br>CCCAGACCACTACTTGGTTGATCACACTTTGGACGGTCGTGTTTTGTTCCCTGCTACTGGTTACTTGTCCATCGTCTG<br>GAAAACTCTGGCTAGAGCACTTGGTTTGGGTGTTGAGCAATTGCCAGTTGTTTTCGAGGACGTTGTCTTGCACCAGG<br>CTACTATCTTGCCAAAGACCGGTACTGTTTCCTTGGAGGTTCGTTTGTTGGAGGCTTCCAGAGCTTTCGAAGTTTCCG<br>AGAACGGTAACTTGGTTGTCTCCGGTAAGGTTTACCAATGGGACGATCCAGATCCAAGACTGTTCGATCATCCAGA<br>GTCTCCAACTCCTAATCCAACTGAGCCACTGTTTTTGGCTCAGGCCGAAGTCTACAAAGAGCTGAGATTGAGAGGA<br>TACGACTACGGTCCACACTTCCAGGGTATTTTGGAGGCATCTTTGGAAGGTGATTCCGGTAGACTGTTGTGGAAGG<br>ACAACTGGGTTTCTTTCATGGACACCATGTTGCAGATGTCCATCTTGGGTTCTGCTAAGCACGGTCTGTACTTGCCA<br>ACTAGAGTTACTGCCATTCACATCGACCCAGCTACTCACAGACAGAAGTTGTACACCTTGCAGGATAAGGCTCAG

	GTTGCCGATGTTGTCGTTTCCAGATGGTTGAGAGTTACAGTTGCTGGTGGCGTTCACATTTCCGGATTGCATACTGA<br>ATCTGCCCCAAGAAGGCAGCAAGAACAACAGGTTCCAATCTTGGAGAAGTTCTGCTTCACTCCACACACCGAAGA<br>GGGTTGTTTGTCTGAAAGAGCTGCCTTGCAAGAGGAATTGCAGTTGTGTAAGGGTCTTGTTCAGGCCTTGCAGACTA<br>CTGTTACTCAGCAGGGTTTGAAGATGGTCGTTCCTGGTTTGGATGGTGCTCAGATTCCAAGAGATCCTTCTCAGCAA<br>GAGTTGCCAAGACTTTTGTCCGCTGCTTGCAGATTGCAATTGAACGGAAACTTGCAGTTGGAGTTGGCCCAAGTTCT<br>GGCTCAAGAAAGACCAAAGTTGCCTGAGGACCCTTTGTTGTCCGGTTTGCTTGATTCTCCAGCTTTGAAGGCTTGTT<br>TGGACACTGCCGTTGAAAACATGCCATCCTTGAAGATGAAGGTCGTTGAGGTTTTGGCAGGTCACGGTCACTTGTA<br>CTCAAGAATCCCTGGTTTGCTGTCCCCACATCCACTGTTGCAATTGTCCTACACTGCTACCGATAGACACCCACAGG<br>CTTTGGAAGCTGCTCAAGCTGAGTTGCAACAACATGACGTTGCTCAAGGTCAATGGACCCAGCTGATCCTGCTCC<br>ATCAGCTTTAGGTTCTGCTGACTTGTTGGTCTGTAACTGTGCTGTTGCTGCTCTTGGTGATCCAGCTTCTGCTTTGTCC<br>AACATGGTTGCCGCTTTGAGAGAAGGTGGTTTCTTGTTGCTGCACACCTTGTTGAGAGGTCACCCATTGGGAGATAT<br>TGTCGCTTTCTTGACTTCCACCGAGCCACAATACGGTCAAGGTATTTTGTCCCAGGACGCTTGGGAATCCTTGTTCT<br>CCAGAGTTTCTTTGAGACTGGTCGGTCTGAAGAAGTCTTTCTACGGTTCCACTCTGTTCTTGTGCAGACGTCCAACTC<br>CACAAGACTCCCCAATTTTCTTGCCAGTCGATGACACTTCCTTCAGATGGGTTGAGTCCTTGAAGGGTATCTTGGCT<br>GACGAAGATTCCTCCAGACCAGTTTGGTTGAAGGCTATCAACTGTGCTACCTCTGGTGTTGTCGGTCTGGTTAACTG<br>CTTGAGAAGAGAACCTGGTGGTAACAGATTGAGATGCGTCTTGCTGTCTAACCTGTCCTCTACTTCTCACGTTCCAG<br>AAGTTGATCCAGGTTCCGCTGAATTGCAAAAGGTGTTGCAAGGTGACCTGGTCATGAACGTCTACAGAGATGGTGC<br>TTGGGGTGCCTTCAGACACTTTTTGTTGGAAGAGGATAAGCCCGAGGAACCTACTGCTCACGCTTTTGTTTCCACCT<br>TGACTAGAGGTGACTTGTCCTCCATTAGATGGGTCTGCTCCTCATTGAGACATGCTCAACCTACTTGTCCTGGTGCT<br>CAACTGTGTACTGTTTACTACGCCTCCTTGAACTTCAGGGACATTATGTTGGCCACTGGTAAGTTGTCACCTGACGC<br>TATTCCAGGTAAGTGGACTTCTCAAGACTCCTTGCTTGGTATGGAATTTTCCGGTAGGGACGCTTCCGGTAAGAGAG<br>TTATGGGACTTGTTCCAGCCAAAGGTTTGGCTACTTCTGTTTTGCTGTCTCCCGATTTCTTGTGGGACGTTCCATCTA<br>ACTGGACTTTGGAAGAAGCTGCTTCCGTTCCAGTCGTTTACTCCACTGCTTACTATGCCTTGGTCGTTAGAGGTAGA<br>GTTAGACCAGGTGAGACTTTGCTGATTCACTCCGGTTCTGGTGGTGTAGGTCAAGCTGCTATTGCTATTGCCTTGTCC<br>TTGGGTTGTAGAGTGTTCACTACTGTTGGTTCCGCCGAGAAGAGAGCTTACTTGCAAGCTAGATTCCCTCAGCTGGA<br>CTCTACTTCCTTTGCTAACTCCAGAGACACCTCCTTCGAGCAACATGTTTTGTGGCACACAGGTGGTAAGGGTGTCG<br>ATTTGGTTTTGAACTCCTTGGCCGAGGAAAAGTTGCAGGCTTCCGTTAGATGTTTGGCCACTCACGGAAGATTCTTG<br>GAGATCGGTAAGTTCGACCTGTCTCAGAACCATCCACTTGGCATGGCCATTTTCTTGAAGAACGTTACCTTCCACGG<br>TGTCTTGCTGGACGCTTTCTTCAACGAATCTTCTGCTGATTGGAGAGAGGTCTGGGCTCTTGTTCAAGCTGGTATTCG<br>TGACGGTGTTGTCAGACCATTGAAGTGCACTGTTTTCCATGGTGCCCAAGTTGAGGACGCCTTCAGATATATGGCTC<br>AGGGTAAGCACATCGGTAAGGTCGTCGTTCAAGTTTTGGCCGAAGAACCTGAGGCTGTTCTTAAGGGTGCTAAGCC<br>AAAGTTGATGTCCGCCATCTCTAAGACTTTTTGCCCAGCTCACAAGTCCTACATTATCGCCGGTGGTTTAGGTGGTT<br>TTGGTTTGGAATTGGCTCAGTGGCTGATCCAGAGAGGTGTTCAGAAATTGGTTCTGACCTCCAGATCCGGTATCAG<br>AACTGGTTACCAAGCCAAGCAAGTGAGAAGATGGCGTAGACAGGGTGTTCAAGTTCAGGTTTCAACTTCCAACAT<br>CAGCTCCCTGGAAGGTGCTAGAGGATTGATTGCTGAAGCTGCACAATTGGGTCCAGTTGGTGGTGTTTTTAACTTGG<br>CCGTCGTTCTGAGAGATGGTCTGTTGGAAAACCAGACTCCTGAGTTTTTCCAGGACGTGTGCAAGCCTAAGTACTC<br>CGGTACTTTGAACTTGGACAGAGTTACCAGAGAGGCTTGTCCTGAGTTGGACTACTTCGTTGTTTTCTCCTCCGTTTC<br>CTGCGGTAGAGGTAATGCTGGTCAATCCAACTACGGTTTCGCTAACTCCGCCATGGAAAGAATCTGTGAGAAGCGT<br>AGACACGAGGGTTTGCCAGGATTGGCTGTTCAATGGGGTGCTATTGGTGACGTCGGAATCTTGGTTGAGACTATGT<br>CCACCAACGACACTATCGTTTCTGGTACTCTGCCTCAGAGAATGGCTTCCTGTTTGGAAGTTTTGGACCTGTTCTTGA<br>ACCAGCCACACATGGTTTTGTCCTCATTCGTTCTGGCTGAAAAGGCTGCTGCTTACAGAGACAGAGACTCTCAGAG<br>AGATTTGGTTGAGGCCGTTGCTCACATTCTGGGTATTAGAGACTTGGCTGCCGTTAACTTGGACTCCTCCTTAGCTG<br>ATTTGGGTCTTGACTCTCTGATGTCCGTTGAGGTCAGACAGACCTTGGAAAGAGAGCTGAACTTGGTCTTGTCCGTG<br>AGAGAAGTTAGACAGTTGACCCTGAGAAAGCTGCAAGAATTGTCCTCTAAGGCTGACGAGGCTTCTGAGTTGGCA<br>TGTCCTACTCCAAAAGAGGATGGTTTGGCCCAACAACAGACCCAGTTGAACTTGAGGTCCTTGTTGGTTAACCCTG<br>AGGGTCCAACTCTGATGAGATTGAACTCTGTTCAGTCCTCCGAGAGGCCTTTGTTTTTGGTTCACCCTATTGAGGGTT<br>CCACTACCGTGTTTCATTCTTTGGCCTCCAGACTGTCTATCCCAACTTACGGTCTGCAATGTACTAGAGCTGCTCCAT<br>TGGACTCCATTCACTCATTGGCCGCTTACTACATCGACTGCATTAGACAGGTTCAACCAGAGGGTCCATACAGAGT<br>TGCTGGATACTCTTACGGTGCCTGTGTTGCTTTCGAGATGTGTTCCCAATTGCAAGCTCAACAATCCCCAGCTCCAA<br>CTCACAACTCCTTGTTTTTGTTCGACGGTTCCCCTACCTACGTCTTGGCTTACACACAATCCTACAGAGCCAAGTTG<br>ACTCCTGGTTGTGAAGCTGAGGCTGAGACTGAGGCTATCTGTTTCTTCGTTCAGCAGTTCACCGACATGGAACACA<br>ACAGAGTTTTGGAAGCCCTGCTGCCATTGAAGGGACTTGAGGAAAGAGTCGCAGCAGCTGTTGACCTGATTATCA<br>AGTCCCACCAAGGTTTGGACCGTCAAGAGTTGTCTTTTGCCGCCAGATCCTTTTACTACAAGTTGAGAGCTGCTGAG<br>CAGTACACTCCAAAGGCTAAGTACCACGGTAACGTCATGCTTTTGAGAGCTAAGACTGGTGGTGCTTACGGTGAAG<br>ATTTGGGTGCTGACTACAACTTGTCCCAAGTTTGTGACGGTAAGGTGTCCGTCCACGTTATTGAAGGTGACCACAG<br>AACTTTGCTGGAAGGTTCTGGTCTTGAGTCCATCATCTCTATTATCCACTCCTCACTGGCCGAGCCAAGAGTTTCTGT<br>TAGAGAGGGTTAA
BaPPT1	ATGTTGGACAACAGAGAAGCCATGACCGTTGGTGTTGACTTGGTCCACATTCCAGGTTTCGCTGAGCAATTGTCTA<br>GACCAGGTTCCACTTTCGAGCAAGTGTTCTCTCCATTGGAAAGAAGGCACGCTCAGACTAGAAGAGATGCTGCTGC<br>TGACGCTACCAACTCTTCATTGGCTGGATCTAGAACTGAGCACTTGGCTGGTAGATGGGCTGCTAAAGAGGCTTTC<br>ATTAAGGCTTGGTCCCAGGCCATCTACGGTAAGCCACCAGTTATTGAACCAGACTTGGTTAACTTCGCCGAGATCG<br>AAGTTTTGCCAGACAGATGGGGTAGAGTTGCCTTGCAATTGAAGGGTGAAGTTGCCGCTAAGCTGCAAGAATCCAT<br>TGGTGACGTTGAGTTGGCCTTGTCTATTTCTCACGATGGTGACTACGCTACTGCCCAGTGTTTGTTGAGATACCAGA<br>GATAA
EcACPS	ATGGCAATATTAGGTTTAGGCACGGATATTGTGGAGATCGCTCGCATCGAAGCGGTGATCGCCCGATCCGGTGATC<br>GCCTGGCACGCCGCGTATTAAGCGATAACGAATGGGCTATCTGGAAAACGCACCACCAGCCGGTGCGTTTTCTGG

	CGAAGCGTTTTGCTGTGAAAGAAGCCGCAGCAAAAGCGTTTGGCACCGGGATCCGCAATGGTCTGGCGTTTAATCAATTTGAAGTATTCAATGATGAGCTCGGCAAACCACGGCTACGGCTATGGGGCGAGGCATTAAAACTGGCGGAAAAGCTGGGCGTTGCAAATATGCATGTAACGCTGGCAGATGAGCGGCACTATGCTTGTGCCACGGTAATTATTGAAAGTTAA
Cypb5	GCTGGTCAATCTGACAAGGACGTCAAGTACTACACCCTGGAAGAGATCCAAAAGCACAAGGACTCTAAGTCCACCTGGGTTATCTTGCACCACAAGGTTTACGACCTGACCAAGTTCTTGGAAGAACACCCTGGTGGTGAAGAGGTCTTGAGAGAACAAGCCGGTGGTGATGCTACTGAAAACTTCGAAGATGTTGGTCACTCCACTGACGCTAGAGAGTTGTCCAAGACTTACATCATCGGTGAGTTGCACCCAGACGACAGATCCAAGATTGCTAAGCCATCCGACACTCTGATCACTACCGTTGAATCTAACTCCTCCTGGTGGACTAACTGGGTTATTCCAGCTATTTCCGCTTTGGCCGTTGCCTTGATGTACAGGTTGTACATGGCTGAGGAC
Cypb5R	GCTGAAACCGAAGAAGAAGAGGATTCCGAGGCTTGGTTGAGATTGAAGCCAGTTGAACCATTGCCATCTCAGTGTTGTGGTTCTGGTTGTTCCCCATGCGTTTTCGACTTGTACTACAGAGACTTGGAGAGATGGGAGACTGCTAGAGCTAGAAACGACAGATCCTTGTTGTCCGGTAAGCAACCACCAGAGTCTCAATCTTGTTCCGCTAAGTTGTCCCCAGAGACTTTCTTGGCTTTCCACATCTCCACCATGGAAAAGGTTACCAAGGACACCTACCTGGTCAGATTCACTTTGCCAGGTAACTCCAGATTGGGTTTGCGTCCAGGTCAGCACTTGATCTTGAGAGGTGTTGTTGACGGTTTGGAGATCCAGAGAGCTTACACCCCAATCTCTCCAGTTACTGCTGAAGGTTACTTCGACGTCCTGATCAAGTGTTACAGAACCGGTTTGATGTCCCAGTACGTCGAGTCTTGGAGAACTGGTGATACTGCCTTTTGGAGAGGTCCTTTCGGTTCATTCCTGTACGAGCCAAAGAAATACGGTGAGTTGTTGATGTTGGCTGCCGGTACTGGTTTGGCTCCAATGGTTCCAATCTTGCAGTCCATTACTGATGACGAGGACGACGAGACTTTCGTTACCTTGGTTGGTTGCTTCAAGACCTTCGAGGGTATCTACCTTAAGACATTCTTCCAAGAGCAGGCCAGATTCTGGAACGTTCAGACCTTCTTCGTTCTGTCCCAAGAGGTTTCTCCAGAGCAATTGCCATGGTCTTACAGAGACAAGACCCACTTCGGTAGATTGGGTCAAGAATTGGTTGCTGAGTTGGTCGCCTGTTGCAGAAGAAAGCCTTTCACCTTGGTTTGTGGTTCCCCAGCTTTCAACGAGGACATGGCTAGATGTTTGTTGTCTGCTGGTTTGACCGAGGACTCCTACTTCTTGTTCTAA
**T2A1**	AGA GCT GAG GGT AGA GGT TCT TTG CTT ACT TGC GGT GAC GTT GAG GAA AAC CCA GGT CCA
**T2A2**	CGTGCCGAAGGACGTGGATCCCTTTTGACCTGCGGAGATGTCGAAGAGAATCCTGGACCT
**ACC1-S1151A fragment**	CGTGCCTGAAGCTATGAGAAACTCCTCCAGcAAtaGgTCtTCcATcTCtATGGATAGAGCAGTTgcCGTCTCCGATTTGACCTTCATGATCAACAAGAATgactcccagccacttcgtACAGGTATCATAATTCCCACAAACCACTTAGATGA

Table S2. Primer list for construction of knock-out cassettes, expression cassettes and CRISPR plasmids

Primer name	Primer sequence 5'-3'
**Primers for initial knock-outs**	
ole1-1-up_fwd	CTCGAGTTTTTCAGCAAGATATCAAACAAAGAGCCCAAGAC
ole1-1-up_rev	ATATACTTGCTGTGTGGCGTAGATGAAG
ole1-1_down_fwd	ACGCCACACAGCAAGTATATATATAAATCGTTAGGGAAATTTTATTAG
ole1-1_down_rev	AGGAGATCTTCTAGAAAGATATCGATGGTTCAGAGGGCAAG
ole1-2_up_fwd	CTCGAGTTTTTCAGCAAGATATCAGTACAAGAACTGCTAGTAGAAC
ole1-2_up_rev	CTGCTCGCTCGCCTGGAATCAAATGGTAG
ole1-2_down_fwd	GATTCCAGGCGAGCGAGCAGTAACACATTTAAATTAC
ole1-2_down_rev	AGGAGATCTTCTAGAAAGATATCTGGTCTCCCTACCATGTTC
pox1_down_fw	ACTGAAAATCACAACGGTGTATTGATTACGTAGTAATGC
pox1_down_rv	AGAATATTGTAGGAGATCTTCTAGAAAGATATCTTTCTTGCTTGGCCC
pox1_up_fw	TTCCGGATGGCTCGAGTTTTTCAGCAAGATATCAGGTGGTGAGTTGC
pox1_up_rv	CGTAATCAATACACCGTTGTGATTTTCAGTTGTTAGAC
faa1_down_fw	CAATCGGCTGCTCGCTTTCTTGAAGTTTTCTTTGTACG
faa1_down_rv	AGAATATTGTAGGAGATCTTCTAGAAAGATATCCCTACGACAATACTTCAG
faa1_up_fw	TTCCGGATGGCTCGAGTTTTTCAGCAAGATATCTTGACAGAATATCTGAGTATG
faa1_up_rv	GAAAACTTCAAGAAAGCGAGCAGCCGATTG
faa2_down_fw	ATTCAAGTCTAAAAAGAAGGCAACAAGGAAACG
faa2_down_rv	AGAATATTGTAGGAGATCTTCTAGAAAGATATCACCCGTTCGAACTG
faa2_up_fw	TTCCGGATGGCTCGAGTTTTTCAGCAAGATATCATGTGGAGTCGACTG
faa2_up_rv	TTCCTTGTTGCCTTCTTTTTAGACTTGAATTTATGACCT
fat1_down_fw	AGAAATGCTATTCAGTGTGAAGCAGATAGTCTTTTC
fat1_down_rv	AGAATATTGTAGGAGATCTTCTAGAAAGATATCAAATATCATGTCAATATGTTG
fat1_up_fw	TTCCGGATGGCTCGAGTTTTTCAGCAAGATATCATTATTACTCATGATGAATCAATTC
fat1_up_rv	ACTATCTGCTTCACACTGAATAGCATTTCTCAAAAGAC
**ACC1 engineering**	
ACC1TEF_down_fw_new	TTCGCCAACGTCGACCGTTTGTCGAACCCGG
ACC1TEF_prom_fw_new	TCGTGTGCAACACGAGGATCCATAACTGTCGCC
ACC1TEF_prom_rv_new	CGGGTTCGACAAACGGTCGACGTTGGCGAATAAC
ACC1TEF_up_hom_rv_new	GACAGTTATGGATCCTCGTGTTGCACACGAC

ACC1_promoter_TEF_donor_down_rv	AGAATATTGTAGGAGATCTTCTAGAAAGATATCCTCTGGGGTAGCC
ACC1_promoter_TEF_donor_up_fw	TTCCGGATGGCTCGAGTTTTTCAGCAAGATATCCGTAGAGGCCACC
ACC1phosmut_new_down_fw	TATCATAATTCCCACAAACCACTTAG
ACC1phosmut_new_up_rev	CTGGAGGAGTTTCTCATAGCTTC
ACC1_phos-mut_donor_down_rv	AGAATATTGTAGGAGATCTTCTAGAAAGATATCCTGAAACGTTGTTAATTAAAGCAC
Acc1_phos-mut_donor_up_fw	TTCCGGATGGCTCGAGTTTTTCAGCAAGATATCTGCACTTCATTCAAGGTTAC
**Knock-out cassettes for neutral lipid storage**	
LRO1_up_fwd	CTCGAGTTTTTCAGCAAGATATCGAACTCTAGCTGTTGTCCGC
LRO1_up_rev	GATGTAAACAAGGTGAAAGGCTGACGGC
LRO1_down_fwd	CCTTTCACCTTGTTTACATCTGTGAGTTGTAAAC
LRO1_down_rev	AGGAGATCTTCTAGAAAGATATCGGGTTCCTTTTGATCTGGTC
ARE2_up_fwd	CTCGAGTTTTTCAGCAAGATATCATTTTCAATAACTACATAAGCCTCTATG
ARE2_up_rev	CCTCCATTTATAAATAGGGATATACTTATTTACATGGG
ARE2_down_fwd	TCCCTATTTATAAATGGAGGTGAAAGTTTGATTATTC
ARE2_down_rev	AGGAGATCTTCTAGAAAGATATCTACATAATCATACATGAAAAATGAACTAC
DGA1_up_fwd	CTCGAGTTTTTCAGCAAGATATCCTTTGATCAGAGTCTCCCAC
DGA1_up_rev	GGCGATAAAAAGTTGAGCCAGTATCTTTTTTAATTG
DGA1_down_fwd	TGGCTCAACTTTTTATCGCCAGTTTGCG
DGA1_down_rev	AGGAGATCTTCTAGAAAGATATCCTAGGAAGATATAGTTCTGTTTTATTCC
**Rad52 overexpression cassette**	
R52_don_down_F	GAAGATTAAGTGAGAGAGATGCCATTGCTTATATAGGC
R52_don_GAP_F	GTCTAAAAACATATGTTTTTGTAGAAATGTCTTGGTGTC
R52_don_GAP_R	GTCATCGAAAGACATGGTACCTGTGTTTTGATAGTTGTTCAATTG
R52_don_gene_F	CAAAACACAGGTACCATGTCTTTCGATGACGCTG
R52_don_gene_R	CTGACATCCTCTTGATTAATTCGAAGCTGGAGAGTTTTC
R52_don_ter_F	CCAGCTTCGAATTAATCAAGAGGATGTCAGAATGC
R52_don_ter_R	AAGCAATGGCATCTCTCTCACTTAATCTTCTGTACTCTG
R52_don_up_R	CATTTCTACAAAAACATATGTTTTTAGACTTGAATTTATGACCTCTG
**expression cassette for pentose phosphate pathway genes**	
ZWF_do_ARG-TT_F	CTGCACAAGATGTAATATACTGAGTTTGTTAATGATACAATAAACTG
ZWF_do_ARG-TT_R	TTGAACTTTGAAATTGGTACCAATGCGAGGATG
ZWF_do_Down_F	CCTCGCATTGGTACCAATTTCAAAGTTCAACTTCCGC
ZWF_do_DOWN_R	AGAATATTGTAGGAGATCTTCTAGAAAGATATCATGCTCTCAAGGACTACC
ZWF_do_pHHX1_F	ATAGATTTGTACCATTTTTCTTTACCTGGATATAAATAAAAAAAAGGAAAC
ZWF_do_pHHX1_R	TTTCGTATCGGTCATGTTTTATCGATAGTAGTTGAGCAATAAAAAAAAG
ZWF_do_SOL_F	AAAGCTTCAAGCAATTCAGTATTTCGAAGTAGAAACGG
ZWF_do_SOL_R	TCCAGGTAAAGAAAAATGGTACAAATCTATTCCTATGAACG
ZWF_do_TEF-TT_F	CTACTAGCCTTAAGATAGACAGATTCATTGACTCTATGATC
ZWF_do_TEF-TT_R	ACTTCGAAATACTGAATTGCTTGAAGCTTTAATTTATTTTATTAAC
ZWF_do_UP_F	TTCCGGATGGCTCGAGTTTTTCAGCAAGATGATATCGAGAATGGTGGGATTTG
ZWF_do_UP_R	CAATGAATCTGTCTATCTTAAGGCTAGTAGTGATTGTTC
ZWF_do_ZWF_F	TACTATCGATAAAACATGACCGATACGAAAGCC
ZWF_do_ZWF_R	AACAAACTCAGTATATTACATCTTGTGCAGCACATC
**NADP+ dependent GDH expression cassette**	
GDH_do_AOX1TT_F	TCAAGAGGATGTCAGAATGC
GDH_do_aox1TT_R	AAGTCCGAAGAAATCGCACAAACGAACGTCTC
GDH_do_down_F	GACGTTCGTTTGTGCGATTTCTTCGGACTTTTGCTTAC
GDH_do_down_R	AGAATATTGTAGGAGATCTTCTAGAAAGATCCCGGGGGTACGTGCTGAAC
GDH_do_GDH_F	TTCAATCAATTGAACAACTATCAAAACAC
GDH_do_GDH_R	CAGGCAAATGGCATTCTG
GDH_do_pGAP_F	TCCGACTGTAACCCCGGATCCTTTTTTGTAGAAATGTCTTG
GDH_do_pGAP_R	TGTGTTTTGATAGTTGTTCAATTGATTG
GDH_do_UP_F	TTCCGGATGGCTCGAGTTTTTCAGCAAGATCCCGGGGGCCCGACG
GDH_do_up_R	TACAAAAAAGGATCCGGGGTTACAGTCGGAGTC
**ACL shuttle expression cassette**	
ACL_ACLp1_F	ACCACCAAATGGGTAAACC
ACL_ACLp1_R	CATTGATATACAAGATCTATCACAAACAC
ACL_ACLp2_F	CTCCTAACTAAAACTGTAAAGACTTCC
ACL_ACLp2_R	CTGTTGCTGCAATGGTTTACCC
ACL_CTP1_F	AACTATATGAACTAACTAATTAAGCAGAAGCAGGAC
ACL_CTP1_R	TTTGTAAGAGTAACAATGCCAGAGAAAAGGAAGG
ACL_dasTT_F	CTAGATCTTAGTGTTGACCCTTGTGACTGACAC
ACL_dasTT_R	ACGGGAAGTCTTTACAGTTTTAG
ACL_down_F	CCTCCTCTCTTGTGTAATGCTAGCCTAGTTGTCAGAG

ACL_down_R	AGAATATTGTAGGAGATCTTCTAGAAAGATCCCGGGGTAAAAACACATAAAACTTG
ACL_htbTT_F	GCTTTCTGATATGTAACATATTTATGAGTTG
ACL_htbTT_R	TTCGCGTCGATTTGGTGTGACAAGTTACATCGTTTTCTG
ACL_MDH_F	CTACAACAATCAAAGATGGTTAAAGTCACAGTTTGCG
ACL_MDH_R	AAAGCTTCAAGCAATTTAGTTGCCAGCAATGAAGG
ACL_ME_F	TTCAATCAATTGAACAACTATCAAAAC
ACL_ME_R	AACTCATAAATATGTTACATATCAGAAAGC
ACL_pGAP_F	GAGTGTCTCGTTGAATTTTTTGTAGAAATGTCTTGGTGTC
ACL_pGAP_R	TGTGTTTTGATAGTTGTTCAATTGATTG
ACL_pgk1TT_F	ATGTAACTTGTCACACCAAATCGACGCGAAAG
ACL_pgkTT_R	CTTCTGCTTAATTAGTTAGTTCATATAGTTTGAATTCTGATTTTGATG
ACL_pRP_F	CCTTTTCTCTGGCATTGTTACTCTTACAAAGAACAAGTTTTTTG
ACL_pRP_R	TGTGACTTTAACCATCTTTGATTGTTGTAGTTAACCTGG
ACL_pTPI_F	TGTGTTTGTGATAGATCTTGTATATCAATG
ACL_pTPI_R	CATTTCTACAAAAAATTCAACGAGACACTCTTCC
ACL_tefTT_F	ATTGCTGGCAACTAAATTGCTTGAAGCTTTAATTTATTTTATTAAC
ACL_tefTT_R	AACTAGGCTAGCATTACACAAGAGAGGAGGCAG
ACL_up_F	TTCCGGATGGCTCGAGTTTTCAGCAAGATCCCGGGTTCAAATTTTTAACCTCGTC
ACL_UP_R	TCAGTCACAAGGGTCAACACTAAGATCTAGAGAATCTGAC
**Elongases knock-out cassettes**	
Elo2_down_F	ACACCAACTATCAAGTTGTGCTGGCCTAAGGAC
Elo2_down_R	AGAATATTGTAGGAGATCTTCTAGAAAGATATCCGTTTGCTACTGTATATCC
Elo2_up_F	TTCCGGATGGCTCGAGTTTTCAGCAAGATATCATATGTTGAAGGTACTACAGC
Elo2_up_R	CTTAGGCCAGCACAACTTGATAGTTGGTGTACTAAAAGG
Elo3_down_F	CCCACCATCCAAGTAGTTATAAAATATAGAATAAAAAAAATCAAGTTCGAG
Elo3_down_R	AGAATATTGTAGGAGATCTTCTAGAAAGATATCACGGATAGGTGAGTGG
Elo3_up_F	GTTTTCAGCAAGATATCCAGTGAAGAATGCCAAG
Elo3_up_R	TCTATATTTTATAACTACTTGGATGGTGGGTCTACC
**DGA2-TGL3-TGL4 expression cassette**	
DGA_DGA_F	TCAAGACTTACAATTAAAATGCCTGAAAAGAAGAACAG
DGA_DGA_R	ATTAAAGCTTCAAGCAATTCACTCGACAATTCGTAGC
DGA_down_F	AATTGGAATGGAAAATTGGCTAGCGCGACTGAAAGCTTATTTTGTTTAC
DGA_down_R	AGCTGAGAATATTGTAGGAGATCTTCTAGAAAGATCCCGGGTCACTATATTTAGCTTGCG
DGA_GAP-TT_F	CCCAAGAATCATAAATAAATCGATTTGTATGTGAAATAGCTG
DGA_GAP-TT_R	AAATAAGCTTTCAGTCGCGCTAGCCAATTTTCCATTCCAATTTGATCG
DGA_HTA-TT_F	CTGCCTCCTCTCTTGTGTCTAACCTTGGTATATTTATAAGAATTCAC
DGA_HTA-TT_R	CAAAGGAAGATGAGGTAGGTTTCTGCTTAGTTCCCAGTTC
DGA_pCAT_F	TGCGTAATTACGGAAAATGCTAGCTAATCGAACTCCGAATGCG
DGA_pCAT_R	GTTCTTCTTTTCAGGCATTTTAATTGTAAGTCTTGACTAGAGC
DGA_pHHX2_F	TCTAGATGGTTGGGGCATTTTTACTACGATAGACACAAGAAG
DGA_pHHX2_R	TGCTCCAAATAAGTTCATATTTATTGATTATTTGTTTATGGGTGAGTC
DGA_TEF-TT_F	CTACGAATTGTCGAGTGAATTGCTTGAAGCTTTAATTTATTTTATTAAC
DGA_TEF-TT_R	TAAATATACCAAGGTTAGACACAAGAGAGGAGGC
DGA_TGL3_F	TGGGAACTAAGCAGAAACCTACCTCATCTTCCTTTGGTTG
DGA_TGL3_R_splice	GTATCATATTTTGTTGTGAATTATGTCCCTGCACCC
DGA_TGL3_F_splice	GGGTGCAGGGACATAATTCACAACAAAATATGATACTTCCAAAC
DGA_TGL3_R	GTGTCTATCGTAGTAAAAATGCCCCAACCATCTAG
DGA_TGL4_F	AACAAATAATCAATAAATATGAACTTATTTGGAGCAGTCAC
DGA_TGL4_R	TTTCACATACAAATCGATTTATTTATGATTCTTGGGACCCC
DGA_up_F	AGATCTTCCGGATGGCTCGAGTTTTCAGCAAGATCCCGGGAATGTCTGTGGGGAG
DGA_up_R	GCATTCGGAGTTCGATTAGCTAGCATTTTCCGTAATTACGCACAG
***S. cerevisiae* OLE1 expression cassette**	
Scole1_DASTT_F	ACTGGTAAGTTCTTTTAAACGGGAAGTCTTTACAGTTTTAG
Scole1_DASTT_R	AAACTAACTGTTATAGACGACCCTTGTGACTGACAC
Scole1_ole1_F	AACAACTATCAAAACACAATGCCAACTTCTGGAACTAC
Scole1_down_F	GTGTCAGTCACAAGGGTCGTCTATAACAGTTAGTTTTCAAAACATTC
Scole1_down_R	AGCTGAGAATATTGTAGGAGATCTTCTAGAAAGATCCCGGGAAACCAGATAAGTGCAC
Scole1_GAP_F	TTTGTCTTTGTTTGTCTCTTTTTTGTAGAAATGTCTTGGTGTC
Scole1_GAP_R	AGTTCCAGAAGTTGGCATTGTGTTTTGATAGTTGTTCAATTG
Scole1_ole1_R	ACTGTAAAGACTTCCCGTTTAAAAGAACTTACCAGTTTCGTAG

Scole1_up_F	AGATCTTCCGGATGGCTCGAGTTTTTCAGCAAGATCCCGGGTTGCGTCAAGGAGG
Scole1_up_R	AGACATTTCTACAAAAAAGAGACAAACAAAGACAAAGACAAG
**'MGA2 expression (from Chr4_NS2 locus)**	
MGA_down_F	CCTCCTCTCTTGTGTTCATCTCCGACTAGACACG
MGA_down_R	AGAATATTGTAGGAGATCTTCTAGAAAGATATCTGAAAAAAGTAATAAATCTAAGTTTCATG
MGA_MGA_F	CAATTGATTACGAAAATGAACGAAGCATCTTTGG
MGA_MGA_R	AAAGCTTCAAGCAATCTATAGCTCCATAACCTTCAAAAG
MGA_PGK_R	AGATGCTTCGTTCATTTTCGTAATCAATTGGGCTATG
MGA_pPGK_F	TCAAGTTCAGCAGCGAAGTTGGTACCCAGCCG
MGA_TEF1TT_F	GTTATGGAGCTATAGATTGCTTGAAGCTTTAATTTATTTTATTAACA
MGA_TEF1TT_R	TCTAGTCGGAGATGAACACAAGAGAGGAGGCAG
MGA_UP_F	TTCCGGATGGCTCGAGTTTTTCAGCAAGATATCCAAAACACAATACATCACG
MGA_UP_R	GCTGGGTACCAACTTCGCTGCTGAACTTGATTATG
**FAS constructs**	
TEFup UP fw	GGATGGCTCGAGTTTTTCAGCAAGATATTTAAATGTAGTACTTCATCTGCTTCGG
TEFup UP rev-AOX1TT	CCTCTTCAGAGTACAGAAGATTAAGTGAGAAGGCCAGACAGGATTGG
PpFAS1 up fw	CGGATGGCTCGAGTTTTTCAGCAAGATATTTAAATCTTCTAAGGGTATCTTTGGAAACAC
PpFAS1 up rev-AOX1TT	GAAGATTAAGTGAGAATCAAATATTCGCCCTTGAAGTAGTAG
AOX1TT fw	GAGATACCAGAGATAATCAAGAGGATGTCAGAATGCCATTTG
AOX1term rev	TCTCACTTAATCTTCTGTACTCTGAAGAGG
AOX1TT rev-PpFAS1up	GGGCGAATATTTGATTCTCACTTAATCTTCTGTACTCTGAAGAGG
BaPPT1 fw	CAAACTATATTAAAACTACAACAATGTTGGACAACAGAGAAGCC
BaPPT1 rev	TGACATCCTCTTGATTATCTCTGGTATCTCAACAAACACTGG
AOX1term-acpS rev	CAGGCAAATGGCATTCTGACATCCTCTTGATTAACTTTCAATAATTACCGTGGCACAAGC
pHTX-acpS fw	CCATCTCATACTCAAACTATATTAAAACTACAACAATGGCAATATTAGGTTTAGGCACGG
AOX1TT-ScFAS2 rev	CTCAGGCAAATGGCATTCTGACATCCTCTTGACTATTTCTTAGTAGAAACGGCGACCGC
pHTX1-ScFAS2 fw	CCATCTCATACTCAAACTATATTAAAACTACAACAATGAAGCCGGAAGTTGAGCAAG
pHTX1 fw	GTTGTCCAACATTGTTGTAGTTTTAATATAGTTTGAGTATGAGATGG
pHTX1 rev	TTTGATTTGTTTAGGTAACTTGAACTGGATG
ScFAS1 fw	CATCCAGTTCAAGTTACCTAAACAAATCAAAATGGACGCTTACTCCACAAGAC
ScFAS1 rev	ACTGATTAAGCAAATGTAATTAAATAAAACGATTAGGATTGTTCATACTTTTCCCAGTTG
hFAS_1 fw	CATCCAGTTCAAGTTACCTAAACAAATCAAAATGGAAGAGGTTGTTATCGCTG
hFAS_1 rev	AAAGCAGAAGCTGGATCACCAAGAGC
hFAS_2 fw	CTGCTCTTGGTGATCCAGCTTCTGC
hFAS_2 rev	AAACTGATTAAGCAAATGTAATTAAATAAAACGATTAACCCTCTCTAACAGAAACTCTTG
BaFAS_1 fw	CATCCAGTTCAAGTTACCTAAACAAATCAAAATGACCATCGGTATCTCCAACC
BaFAS_1 rev	ATACCAGGGATCATGATGAAGGATCTCATACC
BaFAS_2 fw	TATGAGATCCTTCATCATGATCCCTGGTATC
BaFAS_2 rev	CCCAAACTGATTAAGCAAATGTAATTAAATAAAACGATTATTGGTGGGAAGCAGTTGATC
TEFup DOWN fw	CTCGTGTCAGATTGCGTATCAAG
TEFup DOWN rev	AGAATATTGTAGGAGATCTTCTAGAAAGATATTTAAATTGGGACGAATGGGACAG
TEFup DOWN-BaFAS rev	CTTGATACGCAATCTGACACGAGTTATTGGTGGGAAGCAGTTGATCTCTTC
TEFup DOWN-hFAS rev	CTTGATACGCAATCTGACACGAGTTAACCCTCTCTAACAGAAACTCTTGGC
TEFup DOWN-ScFAS1 rev	CTTGATACGCAATCTGACACGAGTTAGGATTGTTCATACTTTTCCCAGTTGTC
PpFAS1 down fw	TCGTTTTATTTAATTACATTTGCTTAATCAGTTTGGG
PpFAS1 down rev	GCTGAGAATATTGTAGGAGATCTTCTAGAAAGATATTTAAATTCTGGTTGGTTCACGCTG
**Expression from His4 locus**	
His4_Down_F_TTArg4	CCTCGCATTGGTACCGAAGTTCCTATACTTTCTAGAGAATAGG
His4_down_R_pJET	AGAATATTGTAGGAGATCTTCTAGAAAGATAAATATGCCTGAATTAGGGACTTTG
His4_PGAP_F_His4up	TCTCGCTTGGATCCTTTTTTGTAGAAATGTCTTGGTGTC
His4_pGAP_R_MCS	CTGCAGCATATGGCGGCCGCTTAATTAAGTCGACTGTGTTTTGATAGTTGTTCAATTGATTG

His4_TTArg4_F_MCS	ACTTAATTAAGCGGCCGCCATATGCTGCAGTATACTGAGTTTGTTAATGATACAATAAAC
His4_TTArg4_R_Down	AAGTATAGGAACTTCGGTACCAATGCGAGGATGCTGCTG
His4_up_F_pJET	TTCCGGATGGCTCGAGTTTTTCAGCAAGATAAATGAGCTGAAGAAACTGATTC
HIS4_up_R_PGAP	ACATTTCTACAAAAAAGGATCCAAGCGAGAGAC
Fw_QC_pJet_PstIdel	GCCGCCCCTACAGCCGAAT
Rv__QC_pJet_PstIdel	ATTCGGCTGTAGGGGCGGC
His4_linear_F	AAATGAGCTGAAGAAACTGATTC
His4_linear_R	AAATATGCCTGAATTAGGGACTTTG
**Tefup::CeFAT expression cassette**	
TEFup-CeFAT_DOWN_R	AGAATATTGTAGGAGATCTTCTAGAAAGATATTTAAATTGGGACGAATGGGACAG
TEFup_CeFAT_F_P-PGK	ATGACCCAGATCAAGGTTGACG
TEFupCeFAT_UP_F_pJET	GGATGGCTCGAGTTTTTCAGCAAGATATTTAAATGTAGTACTTCATCTGCTTCGG
Tef1prom_bam-MCS-ARG4ter_rev	GCAATCTGACACGAGATGCGAGGATGCTGCTGGAGAC
pPGK-TEFup UP rev	GTGATCGGCTGGGTACCAACTTAGGCCAGACAGGATTGG
pPGK fw	AAGTTGGTACCCAGCCGATCAC
CeFAT-pPGK rev	CGTCAACCTTGATCTGGGTCATTTTCGTAATCAATTGGGCTATGCTAAGAG
TEF-down_F_TTArg4	GCAGCATCCTCGCATCTCGTGTCAGATTGCGTATCAAG
**ole1-1::CeFAT expression construct**	
Fw_insertGAP_CeFAT-OLE1-1	ATTTCCCTAACGATTTATATATATACTTGCTTTTTGTAGAAATGTCTTGGTGTC
Rv_insert_TT_CeFAT-OLE1-1	TTAGTCTTCTCACTTCATCTACGCCACACAGGTACCAATGCGAGGATG
Ole1-1_up_open_rev	TGTGTGGCGTAGATGAAGTG
Ole1-1_down_open-fw	GCAAGTATATATATAAATCGTTAGGG
**FLDup::MmSCD3 expression construct**	
FLD_UP_F	GTTTTTCTGAATCTGAAAAGCTTACCTTATGAG
FLD_UP_R	GACAGTTATGGATCCTTGGCGAGATCAATGGAAAGG
FLD_Tef1_F	CATTGATCTCGCCAAGGATCCATAACTGTCGCCTCTTTTATC
FLD_TEF1_R	TTTCGGTCGACGTTGGCGAATAACTAAAATGTATGTAGTG
FLD-MmSCD3 F	CACTACATACATTTTAGTTATTCGCCAACGTCGACCGAAACGATGCCAGGTCACTTGCTG
FLD-MmSCD3_R	GTTTATTGTATCATTAACAAACTCAGTATACTGCAGTTAACCGGACTTGTGAGAACCGTC
FLD_ARG4TT F	CTGCAGTATACTGAGTTTGTTAATGATACAATAAAC
FLD_ARG4TT_R	CCGTGCCAGAGATTCATGCGAGGATGCTGCTGGAGAC
FLD_down_fwd	GCAGCATCCTCGCATGAATCTCTGGCACGGTGCTAATG
FLD_down rev	CTGCACTCAGGAGTGTACAGAAG
**SCD3-T2A-MmCyb5-T2A-MmCyb5Red construct**	
T2A-SCD3 rev	ACGTCACCGCAAGTAAGCAAAGAACCTCTACCCTCAGCTCTACCGGACTTGTGAGAACCG
MmCyb5Red-ARG4term fw	GACCGAGGACTCCTACTTCTTGTTCTAATATACTGAGTTTGTTAATGATACAATAAACTG
T2A1 fw	AGAGCTGAGGGTAGAGGTTC
MmCyb5 rev	ACATCTCCGCAGGTCAAAAG
MmCyb5Red fw	AGGACGTGGATCCCTTTTG
MmCyb5Red rev	TTAGAACAAGAAGTAGGAGTCCTCG
**SCD3-T2A-PpCyb5-T2A-PpCyb5Red construct**	
T2A-PpCyb5 fw	TTCTTTGCTTACTTGCGGTGACGTTGAGGAAAACCCAGGTCCATCTGAAGAAGAAGAACTCAAGG
T2A-PpCyb5 rev	ACATCTCCGCAGGTCAAAAGGGATCCACGTCCTTCGGCACGCTGGTTGAAGAAATAGTATGCAAC
T2A-PpCyb5Red fw	TCCCTTTTGACCTGCGGAGATGTCGAAGAGAATCCTGGACCTGACAATAACGTTGTTGTATTCAC
PpCyb5Red rev	CTAAAAGACAAATACTTGATCAGCCAAC
PpCyb5Red-ARG4term fw	GTTGGCTGATCAAGTATTTGTCTTTTAGTATACTGAGTTTGTTAATGATACAATAAACTG
**PK-PTA construct**	
PK_UP_F	TCTTCCGGATGGCTCGAGTTTTTCAGCAAGATATCTACTATGATTGTAGATAGATAATGC
PK_UP_R	TTCAAACTTCTCACCTCCTTATTGTTTCTGATATAATTAGTTTGACGTG
PK_FDH1TT_F	TATATCAGAAACAATAAGGAGGTGAGAAGTTTGAAGG
PK_FDH1TT_R	GCAGGTGACAACGAATAATTGAAATGTATTTAATTTGATATTAAGTAAATGAATG
PK_BbPK_p2_R	AATTAAATACATTTCAATTATTCGTTGTCACCTGCAG
PK_BbPK_p2_F	ACAGTTGGAAGCTACAAGAACTC

PK_BbPK_p1_R	TATAGGCTCCCAGAGTTCTTG
PK_BbPK_p1_F	GTGTCTATCGTAGTAAAAATGACCAG
PK_pHHX2_F	ATTACAGGACTGGTCATTTTTACTACGATAGACACAAGAAG
PK_pHHX2_R	GTTCTCCATAAGTTTCATATTTATTGATTATTTGTTTATGGGTGAG
PK_CkPTA_R	CAGTGCCAATCGAACGC
PK_CkPTA_F	ACAAATAATCAATAAATATGAAACTTATGG
PK_FBP1TT_F	CTCAAGCTCAGGGATAATGCGTTCGATTGGCACTG
PK_FBP1TT_R	AGATTCAGACTCTAAACCCGCGGAACCTTACTTTTC
PK_DOWN_F	AAAAGTAAGGTTCCGCGGGTTTAGAGTCTGAATCTGAATATGAAG
PK_DOWN_R	AGCTGAGAATATTGTAGGAGATCTTCTAGAAAGATATCAATTTCAACTTAGGACTCACG
**additional primers for sole ACL expressed from ENO1 promoter**	
NewACL_ENO1_R	ATGGGCTTAGCGGACATTTTTAGATGTAGATTGTTATAATTGTGTG
NewACL_ENO1_F	GACAACTAGGCTAGCATTCTGGGCAAAATCACACAATTC
NewACL_down_F	TGTGTGATTTTGCCCAGAATGCTAGCCTAGTTGTCAGAG
NewACL_ACL_F	AACAATCTACATCTAAAAATGTCCGCTAAGCCCATTAG
**additional primers for 'MGA2 expression from 2nd locus (Ch1_NS10)**	
NewMGA_UP_R	TCGGCTGGGTACCAACTTGGGGTTACAGTCGGAGTC
NewMGA_MGAfrag_F	ACTCCGACTGTAACCCCAAGTTGGTACCCAGCCG
NewMGA_MGAfrag_R	CAAAAGTCCGAAGAAATCACACAAGAGAGGAGGCAG
NewMGA_DOWN_F	TGCCTCCTCTCTTGTGTGATTTCTTCGGACTTTTGCTTAC
RtIDH1 expression cassette	
IDH1_ctr1	AGGGATTCCACTACACCAGG
IDH1_ctr2	TGCGGATTCCTTCTTGGACG
IDH1_ctr3	CTTGTCCTTGATGTCAAGTCC
IDH1_DOWN_F	AGTGGTTTTGGAAACATTGTAATGTTATGTTTGATTGG
IDH1_DOWN_R	AGCTGAGAATATTGTAGGAGATCTTCTAGAAAGATCCCGGGTCCTCAATTCCCTGACC
IDH1_RtIDH1_F	ATCAAACATAACATTACAATGTTTCCAAAACCACTGG
IDH1_RtIDH1_R	GTAGGAGGGCTAGATCTTTATTGCAATCCTTTAATAACTGCATG
IDH1_UP_F	AGATCTTCCGGATGGCTCGAGTTTTTCAGCAAGATCCCGGGCCTCATAAATCTTGTCTTCC
IDH1_UP_R	ATTAAAGGATTGCAATAAAGATCTAGCCCTCCTACAATAG
**RtIDH2 expression cassette**	
IDH2_ctr1	GTGTTACACTTACCTCCTCC
IDH2_ctr2	TCGATTGTGTCAAGATCTGC
IDH2_ctr3	TCTCAACATCATGCATGAGG
IDH2_DOWN_F	GAAGTCCTAGTAAACATGGTGGAATAATCTGAAAG
IDH2_DOWN_R	AGCTGAGAATATTGTAGGAGATCTTCTAGAAAGATCCCGGGAAAATTGGCTGAAC
IDH2_RtIDH2_F	CTTTCAGATTATTCCACCATGTTTACTAGGACTTCTTTGAG
IDH2_RtIDH2_R	TTCATAACCGTTCACTATTATAATTTCTTCAGGATTTCGTCAG
IDH2_UP_F	GATCTTCCGGATGGCTCGAGTTTTTCAGCAAGATCCCGGGTGTTGAAAGAGAGTATCTAC
IDH2_UP_R	ATCCTGAAGAAATTATAATAGTGAACGGTTATGAAAATGAATG
**CRISPR/Cas9 primers**	
Rv_T4pHTX1	GGGCATCACAATCATGGAGC
Fw_T4pHTX1	CCTCGAGAAAGTCGATGGGG
Fw_Gibson_insert20N	ACGAAACGAGTAAGCTCGTCNNNNNNNNNNNNNNNNNNNNGTTTTAGAGCTAGAAATAGC
Gibson_Rv_insert_HH	GAGCTTACTCGTTTCGTCCTCACGGACTCATCAGNNNNNNTTTGATTTGTTTAGGTAACT

Note for CRISPR plasmid primers: To generate CRISPR plasmids with new target N20 the sequences listed in Supplementary Table 3… (without PAM sequence) were inserted in primer "Fw_Gibson_insert20N" (in place of NNNNNNNNNNNNNNNNNNNN in 5′-3′ orientation). The first six bases from the 5′ end of the respective N20 sequence (HH) were inserted in primer "Gibson_Rv_insert_HH" (in place of NNNNNN in 5′-3′ orientation). E.g., to target the sequence "AAGGTCTGCTCAGTCTGTTG TGG" the following primers were used: ACGAAACGAGTAAGCTCGTC<u>AAGGTCTGCTCAGTCTGTTG</u>GTTTTAGAGCTAGAAATAGC and GAGCTTACTCGTTTCGTCCTCACGGACTCATCAG<u>AAGGTC</u>TTTGATTTGTTTAGGTAACT to generate a new CRISPR plasmid. Underlined bases represent the respective N20 and HH sequences, respectively.

Table S3. CRISPR target sites used in this study

target site	target sequence N20 5′-3′	PAM
FAD12	GGTGTTTGTACCTGCCACAA	TGG
OLE1-1	GGATTCTGGTACTCCCACAT	GGG
OLE1-1	TCTCAGACCTCGCTGATGAC	TGG
OLE1-1	GGTGGTCTGTCAATTACAGC	TGG
OLE1-2	TTCCCGCAATAATTGGCTGG	TGG
POX1	GAAGACGACGCTGGTGCAAT	GGG
POX1	AATCAATTTGAGTTCCTCTG	AGG
FAA1	GTCGGATTGGCAATTTCGGA	TGG
FAA1	TGAAGGCCTTCAGGTCGGAT	TGG
FAA2	AACGCTGAGTATTCTTCCCT	TGG
FAA2	TCCAGCGACAAGCGCTAGAT	TGG
FAT1	GCCACTCATATCCAGTACGT	TGG
FAT1	TAGAGTCTGGTTGAATCCTC	AGG
TesA	AAGGTCTGCTCAGTCTGTTG	TGG
TesA	GAAGCAACACCAGCCAAGAT	GGG
ACC1-S1159A	TAACCGATCCTCTATTTCAA	TGG
ACC1-S1159A	GATTCTCAACCTTTGAGAAC	AGG
ACC1-promoter	AATTTGGTGTATTAACTGGT	AGG
ACC1-promoter	CGATTCAGAGACACGAGAAA	TGG
DGA1	GAGAGATTACTTAATGGCAT	TGG
LRO1	TGGGTTGAAGCTGACGGATA	CGG
LRO1	AGCAACAGCGATTCCTATGA	AGG
LRO1	GCCGATGTGCGATTCAAGTC	TGG
ARE2	TGGATCGCTTCCAGAGCCAA	CGG
ARE2	TCGACTCTCGCCACTGGGAT	CGG
ARE2	ATCTACTCGATACGATAAGA	AGG
Chr1_NS6	GACCCCGTAATAATCGACGT	CGG
Chr1_NS6	TTCCGACGTCGATTATTACG	GGG
Chr1_Ns10	TAGTTCTGTGACATAGTACC	*AGG*
Chr1_Ns10	AGAAATCTCGAAAACACCCC	TGG
Chr2_Ns3	TATAAGGCTCTTGTAGATGG	AGG
Chr2_Ns3	GTGTTCTGCTTGTCGATATA	AGG
Chr3_Ns7	AAAGCTAATTAGTCTCATAA	TGG
Chr3_Ns7	TACCGAAGTCTTCCATAACT	AGG
Chr4_Ns2	AATAATTGCAGGGCAGATGG	TGG
Chr4_Ns2	AAAAGAAATAATTCAATGCA	TGG
CHr4_Ns4	ATGAAAAAGATACGGGGCAG	AGG
CHr4_Ns4	GTTGTCAAATGAAAAAGATA	CGG
Chr4_Ns7	TCAAAAGCTTGATTTATAGC	TGG
Chr4_Ns7	TCTCGATGGACGGATAACAG	AGG
ELO2	GGCCATAGTTCGATGCCAAA	GGG
ELO2	AGCACCGTGGGTGGCTTTAG	AGG
ELO3	ATACCGTATTGAACATACGA	CGG
ELO3	GGCCATAAACGGATTCCAAA	AGG
ELO100	GGACTGAAAGGAGAATCTGG	AGG
ELO100	TACGAGAGGGCAAACACCAA	TGG
FAA2-outer (for RAD52)	GACGAGAGCTTGTTCAAACG	AGG
FAA2-outer (for RAD52)	ATAATTAAAGATTTGGAGGA	GGG
PXA1	GTAGCTTATGAGGTAAGATG	AGG
PXA1	GGTCCAACGGATGTTCAACT	GGG

FAD15	ACATGTTCTCGTCAGTGTGA	CGG
PpFAS1	CGACGACGAGCCGAGCTCCA	AGG
PpFAS1	CAGATCTACGCCATATTTGG	AGG
PpFAS1	CCAAGGTCTGGTCACCGCTG	TGG
PpFAS1	TGCCCCAAGCAAAGACTTGG	TGG
His4	TGCCAAGTACGGTGTGACGT	TGG
His4	AACGAGAGCAGACTACACCA	GGG
TEFup	GCAAGATGGTTAAAAGGTGA	AGG
TEFup	GAATGGGCAAGATGGTTAAA	AGG
FLDup	GCGGCAGTAATTGATATCGT	AGG

Table S4. Cell wet weights from strains cultivated in 96-deep well plates (see methods).

**strain *Pp#***	**WT**	**#28**	**#29**	**#32**	**#35**	**#37**	**#39**	**#51**	**#150**	**#140**
CWW [g/L]	53.8	31.5	27.1	18.6	18.8	18.1	20.6	20.0	16.5	28.3
**strain *Pp#***	**#135**	**#44**	**#45**	**#46**	**#47**	**#48**	**#53**	**#49**	**#50**	**#54**
CWW [g/L]	20.3	20.0	19.3	23.3	34.3	33.3	38.3	22.1	24.1	29.0
**strain *Pp#***	**#121**	**#117**	**#114**	**#142**	**#153**	**#145**	**#141**	**#112**	**#113**	**#67**
CWW [g/L]	26.5	26.1	29.1	31.0	27.1	24.3	22.8	24.3	24.1	25.5
**strain *Pp#***	**#91**	**#85**	**#89**	**#148**	**#138**	**#136**				
CWW [g/L]	25.0	14.3	12.5	13.5	25.0	25.0				

Table S5. Medium compositions tested for free fatty acid production with strain *Pp*#39. Glucose and nitrogen source concentrations as well as carbon to nitrogen (C/N) ratios are given. Carbon and Nitrogen contents for yeast extract and peptone were estimated based on carbon and nitrogen contents commonly found in yeast biomass and casein, respectively. Hence, the exact values may deviate.

**Medium**	**Glucose [g/L]**	**K_2HPO_4 [g/L]**	**KH_2PO_4 [g/L]**	**YNB w/o AS AA [g/L]**	**Nitrogen-source**	**Nitrogen-source concentration [g/L]**	**C/N ratio**
BMD1	30	3.5	13.6	3.8	Ammonium sulfate	10.0	6
BMD2	30	3.5	13.6	3.8	Ammonium sulfate	0.5	113
BMD3	30	3.5	13.6	3.8	Ammonium sulfate	1.0	57
BMD4	30	3.5	13.6	3.8	Ammonium sulfate	1.9	30
BMD5	30	3.5	13.6	3.8	Ammonium sulfate	3.5	16
BMD6	30	3.5	13.6	3.8	Yeast extract	2.0	51
BMD7	30	3.5	13.6	3.8	Yeast extract	4.0	25
BMD8	30	3.5	13.6	3.8	Yeast extract	6.0	17
BMD9	30	3.5	13.6	3.8	Ammonium chloride	0.5	92
BMD10	30	3.5	13.6	3.8	Ammonium chloride	1.0	46
BMD11	30	3.5	13.6	3.8	Ammonium chloride	1.9	24
BMD12	30	3.5	13.6	3.8	Ammonium chloride	3.5	13
BMD13	30	3.5	13.6	3.8	Bacto peptone	2.0	43
BMD14	30	3.5	13.6	3.8	Bacto peptone	4.0	23
BMD15	30	3.5	13.6	3.8	Bacto peptone	6.5	16

Supplementary methods

Supplementary method S1: Extended GC method for separation of C18:1, C18:2 & C18:3

FAMEs were prepared as described in the materials and methods section. FAMEs were quantified using a Shimadzu GC-2010 Plus device with a flame ionization detector FID-2010 plus (GC-FID), equipped with a Zebron ZB-Wax 7hm-g007-11 30m x 0.32 mm x 0.25 µm column and operated with helium as carrier gas. The flow rate was set to 15.4 mL/min (linear velocity 34.2 cm/sec). An injection volume of 1 µl with a split ratio of 5 was applied. The temperature program started with an initial hold at 50 °C for 1 min, followed by a ramp to 150 °C with a linear increase of 10 °C/min, another ramp to 250 °C with a linear increase of 4 °C/min and a final hold of 4 min. Injection and FID temperatures were set to 220 °C and 250 °C, respectively.

Supplementary method S2: Lipid extraction from *P. pastoris* strain and lipid analysis with thin layer chromatography

Selected strains were cultivated in 30 ml BDM11 medium (Table 1) in 300 mL baffled shake flasks, shaken at 130 rpm and 28 °C for 96 h. A suspension volume corresponding to 60 OD_{600} units was centrifuged at 16,000 x g for 1 min and washed once with deionized water. The resulting cell pellet was subjected to lipid extraction using 0.5 mL methanol

and 1 mL chloroform [105] in a glass tube. After an initial 10 min mixing step, 0.3 ml deionized water was added and the samples were mixed for another 10 min. Phase separation was promoted by centrifugation (5 min at 4500 g) and the lower chloroform phase was used for TLC analysis.

The TLC analysis was conducted as previously described with modifications [106]. In brief, lipid extracts were applied on a TLC plate (silica coated aluminum sheet: TLC Silica gel 60, Merck KGaA, 64271 Darmstadt, Germany) using glass capillaries (10 transfers with a 100 µl capillary). The TLC chamber was saturated with solvent 1 (petroleum, diethyl ether and acetic acid 70:30:2; per vol.) for 30 min and the TLC plate was inserted for separation. Separation was stopped when the solvent front reached the upper end of the TLC plate (only solvent 1 was used for separation). After drying, the plate was submerged in charring solution [107] and heated for 20 min at 100 °C to visualize lipid bands.

Note: The references are listed in the bibliography of the main text.

Additional Information

Additional information S1: expression cassette assembly

Expression cassettes targeted to *HIS4* locus

The up- and downstream flanking regions of the *HIS4* locus, *GAP* promoter and *ARG4* transcription terminator were PCR amplified from genomic DNA and assembled with the linearized pJET1.2 blunt end cloning vector via Gibson assembly. The overlaps between the *GAP* promoter and *ARG4* terminator fragments reconstituted a multiple cloning site containing *PstI* and *SalI* restriction sites. A *PstI* restriction site in the vector backbone was abolished by site directed mutagenesis (Quikchange PCR) using primers Fw_QC_pJet_PstIdel and Rv__QC_pJet_PstIdel. The resulting vector and heterologous genes *'TesA, MtFAT-A, AtFAT-B* and *CeFAT* were digested with *PstI* and *SalI* and were ligated to generate the complete expression cassettes. Primers His4_linear_F and His4_linear_R were used to linearize the expression cassettes prior to transformation.

TEFup::CeFAT construct

The *CeFAT* coding sequence and the *ARG4* transcription terminator were amplified from the *HIS4* targeting vector (section Expression cassettes targeted to *HIS4* locus) using primers TEFup_CeFAT_F_P-PGK and Tef1prom_bam-MCS-ARG4ter_rev, were then assembled with all other fragments.

ole1-1::CeFAT expression cassette

The vector bearing the *ole1-1* deletion cassette (pJET1.2-*ole1-1*up-*ole1-1*down) was linearized by PCR amplification with primers Ole1-1_up_open_rev and Ole1-1_down_open-fw. The *CeFAT* expression cassette (*GAP* promoter, *CeFAT* coding sequence, *ARG4* transcription terminator) was amplified from the *HIS4* targeting vector (section Expression cassettes targeted to *His4* locus) using primers Fw_insertGAP_CeFAT-OLE1-1and Rv_insert_TT_CeFAT-OLE1-1 and inserted into the linearized pJET1.2-*ole1-1*up-*ole1-1*down vector to generate a replacement cassette.

ACC1^{S1151A} donor cassette & CRISPR sites

CRISPR target sites were selected in close proximity to the phosphorylation site in the *ACC1* gene. Homology arms for insertion of the donor cassette were amplified from adjacent regions up- and downstream of the phosphorylation site, each with a length of approximately 1000 bp, using the primers Acc1_phos-mut_donor_up_fw, ACC1phosmut_new_up_rev, ACC1phosmut_new_down_fw and ACC1_phos-mut_donor_down_rv. Homology arms were connected via a synthetic DNA fragment that contained the altered phosphorylation site as well as short parts of the adjacent regions. CRISPR target sites in the synthetic DNA fragment were modified (exchange of codons to maintain

the amino acid sequence of Acc1p, but change the DNA sequence within the Cas9 recognition site) to avoid digestion of the donor cassette.

SCD3-T2A-MmCyb5-T2A-MmCyb5Red construct
The expression cassette vector pJET1.2_*FLDup_PTEF1_MmSCD3_TTARG4* was linearized by PCR amplification with primers T2A-SCD3_rev and MmCyb5Red-ARG4term_fw to generate a gap between the *MmSCD3* 3′ end and the *ARG4* transcription terminator. Two synthetic DNA fragments bearing T2A1 and Cytochrome b5 and T2A2 and Cytochrome b5 reductase from *Mus musculus*, both with compatible overhangs, were inserted in the linearized vector through Gibson assembly.

SCD3-T2A-PpCyb5-T2A-PpCyb5Red construct
The expression cassette vector pJET1.2_*FLDup_PTEF1_MmSCD3_TTARG4* was linearized by PCR amplification with primers T2A-SCD3_rev and PpCyb5Red-ARG4term_fw to generate a gap between the *MmSCD3* 3′ end and the *ARG4* transcription terminator. Cytochrome b5 and cytochrome b5 reductase were amplified from *P. pastoris* genomic DNA and T2A sequences were added by the used primers. Both PCR products were inserted into the linearized vector via Gibson assembly.
